# BioMedGraphica: an all-in-one platform for joint textual biomedical prior knowledge and numeric graph generation

**DOI:** 10.1093/bioinformatics/btag355

**Published:** 2026-06-05

**Authors:** Heming Zhang, Shunning Liang, Tim Xu, Wenyu Li, Di Huang, Yuhan Dong, Guangfu Li, Philip Miller, Peter Goedegebuure, Marco Sardiello, Jonathan Cooper, William Buchser, Patricia Dickson, Ryan C Fields, Carlos Cruchaga, Yixin Chen, Michael Province, Philip Payne, Fuhai Li

**Affiliations:** The Center for Translational Bioinformatics (CTBI), Institute for Informatics, Data Science and Biostatistics (I2DB), Washington University School of Medicine, St. Louis, MO 63110, United States; The Center for Translational Bioinformatics (CTBI), Institute for Informatics, Data Science and Biostatistics (I2DB), Washington University School of Medicine, St. Louis, MO 63110, United States; The Center for Translational Bioinformatics (CTBI), Institute for Informatics, Data Science and Biostatistics (I2DB), Washington University School of Medicine, St. Louis, MO 63110, United States; Department of Computer Science and Engineering, Washington University in St. Louis, St. Louis, MO 63130, United States; The Center for Translational Bioinformatics (CTBI), Institute for Informatics, Data Science and Biostatistics (I2DB), Washington University School of Medicine, St. Louis, MO 63110, United States; Department of Computer Science and Engineering, Washington University in St. Louis, St. Louis, MO 63130, United States; The Center for Translational Bioinformatics (CTBI), Institute for Informatics, Data Science and Biostatistics (I2DB), Washington University School of Medicine, St. Louis, MO 63110, United States; Department of Surgery, School of Medicine, University of Connecticut, Farmington, CT 06032, United States; The Center for Translational Bioinformatics (CTBI), Institute for Informatics, Data Science and Biostatistics (I2DB), Washington University School of Medicine, St. Louis, MO 63110, United States; Department of Surgery, Washington University School of Medicine, St. Louis, MO 63110, United States; Siteman Cancer Center, Washington University School of Medicine, St. Louis, MO 63110, United States; Department of Pediatrics, Washington University School of Medicine, St. Louis, MO 63110, United States; Department of Pediatrics, Washington University School of Medicine, St. Louis, MO 63110, United States; Department of Genetics, Washington University School of Medicine, St. Louis, MO 63110, United States; Department of Pediatrics, Washington University School of Medicine, St. Louis, MO 63110, United States; Department of Surgery, Washington University School of Medicine, St. Louis, MO 63110, United States; Siteman Cancer Center, Washington University School of Medicine, St. Louis, MO 63110, United States; Department of Psychiatry, Washington University School of Medicine, St. Louis, MO 63110, United States; NeuroGenomics and Informatics, Washington University School of Medicine, St. Louis, MO 63110, United States; Department of Computer Science and Engineering, Washington University in St. Louis, St. Louis, MO 63130, United States; NeuroGenomics and Informatics, Washington University School of Medicine, St. Louis, MO 63110, United States; Division of Statistical Genomics, Washington University School of Medicine, St. Louis, MO 63110, United States; The Center for Translational Bioinformatics (CTBI), Institute for Informatics, Data Science and Biostatistics (I2DB), Washington University School of Medicine, St. Louis, MO 63110, United States; The Center for Translational Bioinformatics (CTBI), Institute for Informatics, Data Science and Biostatistics (I2DB), Washington University School of Medicine, St. Louis, MO 63110, United States; Department of Computer Science and Engineering, Washington University in St. Louis, St. Louis, MO 63130, United States; Department of Pediatrics, Washington University School of Medicine, St. Louis, MO 63110, United States

## Abstract

**Motivation:**

Multiomics data analysis is essential for scientific discovery in precision medicine. However, translating analysis results of omics data analysis into novel scientific hypotheses remains a significant challenge. Human experts must manually review analysis results and generate new hypotheses based on extensive and interconnected biomedical prior knowledge, which is subjective and not scalable. While large language models can accelerate the discovery, their reasoning improves when grounded in structured, auditable, and comprehensive biomedical prior knowledge. However, biomedical knowledge is scattered across heterogeneous databases that use diverse and inconsistent nomenclature systems, making it difficult to integrate resources into a unified format for scalable analysis. This fragmentation limits the ability of artificial intelligence systems to fully leverage biomedical data for scientific discovery.

**Results:**

We developed BioMedGraphica, a novel all-in-one platform that harmonizes fragmented biomedical resources by integrating 11 entity types and 30 relation types from 43 databases into a unified textual prior knowledge graph containing 2 306 921 entities and 27 232 091 relations. In addition, we present a novel textual-numeric graph (TNG) data structure concept, where textual information captures prior biological knowledge (e.g. transcription start sites, functions, mechanisms), numeric values represent quantitative biomedical features, and the integrated relations can help uncover mechanisms. By bridging prior knowledge with user-specific data, TNG is a novel and ideal data structure for developing novel graph analysis models.

**Availability and implementation:**

The code is available at: https://github.com/FuhaiLiAiLab/BioMedGraphica and BioMedGraphica knowledge graph database can be downloaded from huggingface dataset: https://huggingface.co/datasets/FuhaiLiAiLab/BioMedGraphica

## 1 Introduction

In recent years, the exponential growth of omic datasets (Barretina *et al.* 2012, Yang *et al.* 2013, Bennett *et al.* 2018, Deelen *et al.* 2019, Ghandi *et al.* 2019, CZI Cell Science Program *et al*. 2025, Heimberg *et al.* 2025, Rood *et al.* 2025, Zhang et al. 2025b) has created unprecedented opportunities to advance biomedical research and precision medicine, improve clinical decision-making, and accelerate drug discovery. While the convergence of artificial intelligence (AI) models with massive omic datasets is revolutionizing the paradigm of scientific discovery in precision medicine, this transformation is still in its infancy (Cui *et al.* 2024, Rood *et al*. 2025). For precision medicine applications, omics data analysis often begin by identifying a set of differentially expressed targets and enriched signaling pathways or biological functions, which is followed by human review with the support of online search of extensive and interconnected prior knowledge to generate expert-specific scientific hypotheses to be further evaluated. However, this process is subjective and not scalable. While large language models (LLMs) and agentic AI models (Gottweis *et al.* 2026, Huang *et al.* 2025a, 2025b, Wang *et al.* 2025, Sapkota *et al.* 2026) are transforming scientific discovery through their ability to interpret and reason with human-readable textual information, their reasoning improves when grounded in structured and auditable evidence (Wei *et al.* 2022, Jiang *et al.* 2023, Tan *et al.* 2025). However, no existing AI model is specifically designed to systematically integrate and analyze numeric omics data, human-readable prior biomedical knowledge, and the biomedical topological context of measured entities in the omics data for novel target discovery and hypothesis generation. To facilitate the development of novel AI models for joint analysis of numeric omics, textual and topological data, in this study, we aim to build an all-in-one platform, named BioMedGraphica, covering the textual information of all biomedical entities, and develop a graphical user interface (GUI) for automatically converting the numeric omics data into textually annotated numeric graphs.

It remains an open and challenging task to integrate and harmonize textual data of biomedical entities. The major challenge is that the landscape of biomedical knowledge, especially the detailed textual descriptions, remains highly fragmented, with essential information dispersed across a multitude of publications, databases, and proprietary datasets. This fragmentation presents significant challenges as different sources often employ inconsistent nomenclature and terminology, hindering effective data integration (Hulsen *et al.* 2019). The vast scope of biomedical data, from genes and proteins to clinical phenotypes and diseases, complicates the development of unified solutions, particularly in terms of entity matching and data harmonization. Although some studies have been reported to integrate dispersed biomedical resources across domains, none of them, at the same time, covers the complete set of biomedical entities, incorporates the detailed textual prior knowledge, and supports the integration of textual knowledge with omics data in the format of TNGs (see the comparison in Table 1). For example, OmniPath (Türei *et al.* 2026) is pathway-centric and assembles expert-curated signaling and regulatory information (i.e. molecular interactions, enzyme–PTM [posttranslational modification] links, protein complexes) with intercellular transmitter–receiver roles and is centered on human with mouse/rat via homology translation. However, OmniPath does not provide detailed textual information and multilevel information integration of promoters, genes, transcripts, proteins. Moreover, it does not cover beyond signaling (e.g. metabolites, microbiota, exposures, phenotypes) and does not provide standardized AI-ready exports paired with quantitative multi-omics features. Bioteque (Fernández-Torras *et al.* 2022) distributes precomputed, machine-learning-ready KG embeddings that facilitate modeling but does not perform comprehensive cross-resource/database alignment, offers limited breadth for metabolites, microbiota, and exposures, and provides embeddings rather than an auditable, harmonized KG with AI-ready data. It also does not provide textual information for entities. PharMeBINet (Königs *et al.* 2022) emphasizes pharmacological links in Neo4j, especially drug–drug and drug–ADR relations, but prioritizes pharmacovigilance over end-to-end molecular-to-clinical harmonization, lacks fine-grained entity typing, and does not pair harmonized knowledge with multiomics features at scale. Ontology-driven RNA-interaction KG (Cavalleri *et al.* 2024) deliver depth on RNA-mediated mechanisms but lack cross-domain entity coverage, and provide limited identifier reconciliation. Disease-specific multimodal resources such as an MASLD (Kendall *et al.* 2023) gene-to-outcome database achieve indication depth but are not designed for cross-domain harmonization, fine-grained typing, or standardized graph exports for AI workflows. Hetionet (Himmelstein *et al.* 2017) aggregates multi-domain entities for translational analyses but relies on heterogeneous identifiers with constrained harmonization, limits fine-grained entity resolution, lacks explicit nuclear signaling, and does not provide graph-AI-ready, multiomics-coupled outputs. PrimeKG (Chandak *et al.* 2023) integrates multi-domain entities and relations, but it does not provide textual knowledge, explicitly distinguish genes, transcripts, and proteins, or model nuclear signaling with standardized multiomics-coupled exports. SPOKE (Morris *et al.* 2023) connects 41 specialized databases across molecular to clinical layers. However, both PrimeKG and SPOKE only partially resolve heterogeneous identifiers and lack standardized, auditable, graph-AI-ready exports that incorporate quantitative multiomics features.

**Table 1:** Comparisons with current biomedical knowledge graph databases and BioMedGraphica.

Databases	Completeness of biomedical entities	Textual description/prior knowledge of entities	Mapping textual knowledge to multi-omic data
OmniPath	×	√	×
Bioteque	×	×	×
PharMeBINet	×	×	×
RNA-KG	×	√	×
MASLD	×	×	×
HetioNet	√	×	×
PrimeKG	√	√	×
SPOKE	√	×	×
BioMedGraphica	√	√	√

Compared with existing knowledge graph resources, the unique and major contributions of BioMedGraphica are summarized in Table 1: (i) completeness of biomedical entities, defined here as end-to-end coverage across the bench-to-bedside spectrum (molecular, cellular, pathway, phenotypic/clinical) with clear entity typing and provenance-tracked relations, (ii) rich textual description and prior knowledge at the entity level, and (iii) computable mapping of textual knowledge to multiomics matrices and other numerical biomedical data, forming a text–numeric graph aligned with the underlying topological structure. BioMedGraphica addresses these dimensions as a unified platform for biomedical data alignment and integration, enabling literature-derived knowledge to be seamlessly linked with numerical multiomics and topological signaling networks. Moreover, BioMedGraphica is the first resource to implement an explicit nucleus-level signaling model that distinguishes genes, transcripts, and proteins, providing better resolution.

To address these challenges, *BioMedGraphica* was developed as an advanced platform that transforms the integration and utilization of biomedical data. By integrating data from 43 high-quality biomedical databases, we unify 11 key biomedical entity types—ranging from molecular and cellular biology (i.e. promoters, genes, transcripts, proteins, signaling pathways, metabolites and microbiota) to clinical practice and pharmacology (i.e. exposures, phenotypes, diseases and drugs)—and 30 relations/edge types into a cohesive knowledge graph, resulting in 2 306 921 entities and 27 232 091 relations. By harmonizing across multiple knowledge bases, this study provides one of the most comprehensive biomedical knowledge graphs available today, enabling large-scale exploration of biological and clinical relationships. Meanwhile, for real world biomedical application scenarios, we removed the isolated entities and reconstructed this database with 834 809 entities and 27 232 091 relations, which we refer to as *BioMedGraphica-Conn*. Built on the harmonized nomenclature system, a core innovation of BioMedGraphica is its entity-matching framework, which combines hard matching, based on standardized identifiers across resources, with soft matching, powered by language models such as BioBERT (Lee *et al.* 2020), to generate embeddings that rank potential matches across heterogeneous datasets. This approach enhances entity recognition by providing more accurate and flexible integration compared to traditional rule-based methods, enabling robust, scalable alignment that reduces manual curation while maintaining precision. These matching capabilities are implemented in a user-friendly web interface with an intuitive GUI, accessible at https://app.biomedgraphica.org/. This interface allows researchers, clinicians, and data scientists to input heterogeneous biomedical datasets and receive integrated, structured outputs in an AI-ready textual-numeric graph (TNG) format. The TNG format integrates textual prior biological knowledge, numeric omic values, and knowledge-graph relations, thereby supporting the development of AI models for integrative and interpretable omics data analysis. Conceptually, TNG can be interpreted as a structured feature-augmentation mechanism for graph learning, extending prior efforts that enhance graph AI expressivity by enriching node representations with additional informative signals. From a theoretical perspective, TNG aligns with the feature-augmentation paradigm developed to overcome the expressive limitations of 1-WL-based architectures, where augmented node-level features are introduced to strengthen discriminative capacity. Therefore, within BioMedGraphica, the TNG format integrates three components: textual information representing biomedical prior knowledge and known biological functions, numeric values encoding quantitative features of biomedical entities across multiple levels from molecular measurements to clinical phenotypes, and the corresponding relations in the knowledge graph. By bridging prior knowledge with user-specific data, TNG provides a robust foundation for developing novel graph AI models. Prior work, such as GraphSeqLM (Zhang *et al.* 2025a), has demonstrated that integrating textual features (textual-attributed sequence data, i.e. DNA/RNA/protein sequences) with omic signaling graphs improves prediction accuracy, and BioMedGraphica extends this by including comprehensive text-attributed prior knowledge from diverse data resources. Thus, the AI-ready TNG data can potentially augment LLM by supplying graph-structured mechanistic context, thereby strengthening reasoning (Zhang *et al.* 2026) and supporting the development of next-generation agentic AI systems (Wei *et al.* 2022, Jiang *et al.* 2023, Park *et al.* 2023, Tan *et al.* 2025). Designed with low coupling in entity space and a streamlined pipeline, the platform supports continuous updates and integration of new resources, ensuring its relevance as biomedical knowledge expands.

In summary, our unique contributions of this study are: (i) a harmonized, cross-domain biomedical KG unifying 43 curated databases into 11 entity types and 30 relation types (2 306 921 entities and 27 232 091 relations), plus an application-ready connected release, *BioMedGraphica-Conn*, with 834 809 entities and the same 27 232 091 relations, (ii) a rigorously audited nomenclature and a hybrid entity-matching framework that combines hard identifier alignment with soft, LM-based embedding matching to rank cross-source correspondences, reducing manual curation while preserving precision, (iii) standardized AI-ready exports, including a TNG format that couples prior textual knowledge with quantitative multiomics and graph topology to support graph foundation models, interpretable analyses, and LLM reasoning, (iv) a production web interface that ingests heterogeneous inputs and returns integrated, structured outputs for large-scale modeling, backed by a low-coupling, extensible pipeline that supports continual updates, and (v) an architecture that augments LLMs with graph-structured mechanistic context for agentic AI, positioning BioMedGraphica as an *all-in-one* platform for translational science and a cornerstone for foundation models that improve prediction performance and interpretability while enabling scalable, evidence-based discovery. Together, these advances establish BioMedGraphica as an all-in-one engine for foundation models and agentic AI in biomedicine, paving the way for stronger predictive accuracy, clearer mechanistic insight, and reproducible, large-scale, evidence-driven discovery.

## 2 Materials and methods

### 2.1 Overview of data resources used in BioMedGraphica

**Figure 1:**
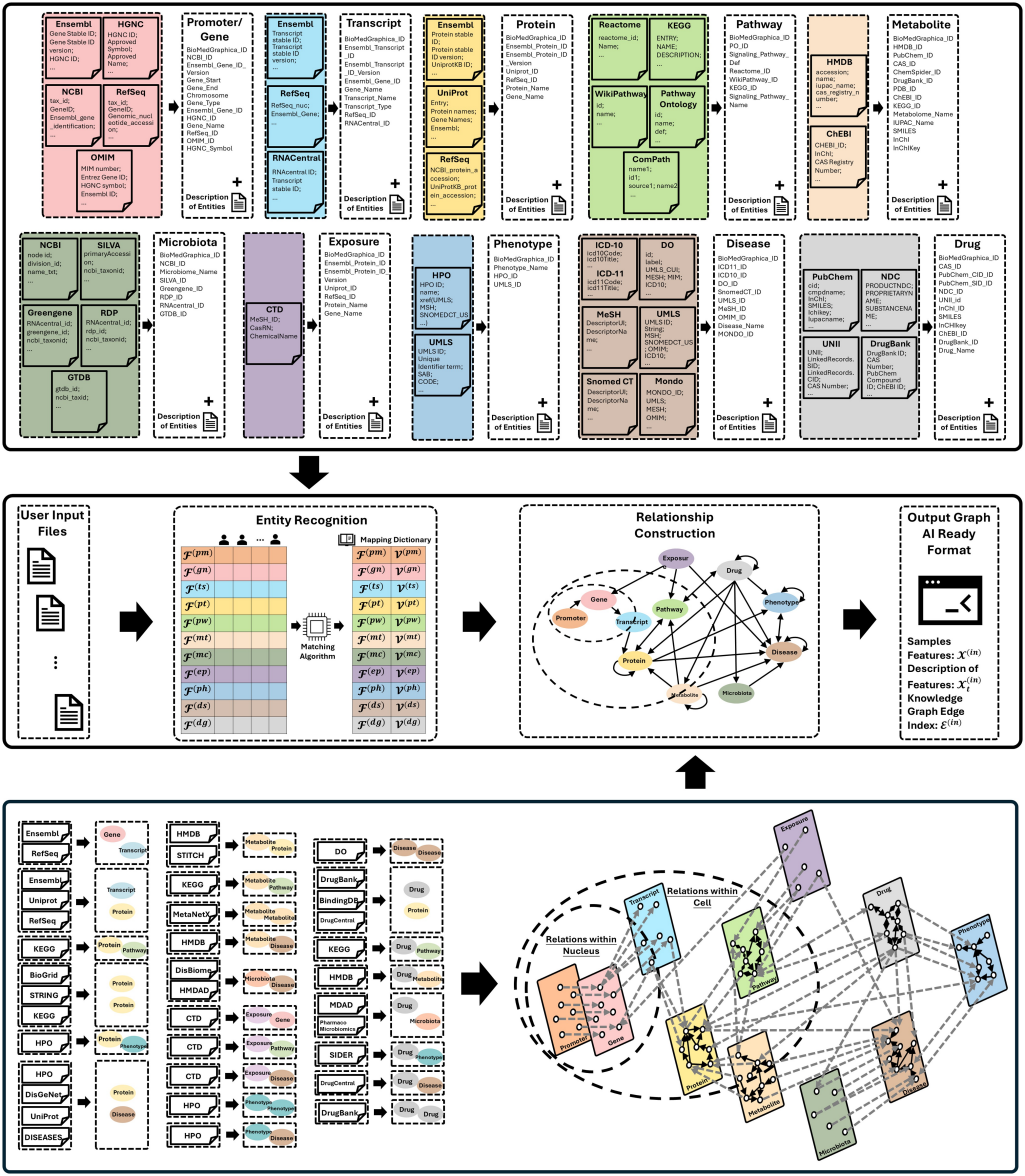
Overview of *BioMedGraphica*. The upper panel shows integration of the entities from various databases. The lower panel demonstrates the relation harmonization process and construction of a knowledge graph. The middle panel displays the general procedures of BioMedGraphica, with entity recognition and relationship construction based on user-specific input files, outputting the graph AI ready format files.

To enable robust biomedical entity recognition and relation extraction, BioMedGraphica integrates a diverse and well-curated collection of biomedical data resources. These resources span both entity databases, which catalog structured identifiers and metadata for various biological and chemical entities, and relation databases, which capture known associations and interactions across biological systems. Together, these datasets form the foundation for harmonizing multiscale biomedical knowledge, supporting the accurate construction of structured graphs for downstream applications in biomedical discovery and precision medicine (see Fig. 1). The following subsections describe the data collection strategies, integration methods, and scope of both entity and relation datasets utilized in BioMedGraphica.

#### 2.1.1 Entity database introduction and collection

**Figure 2:**
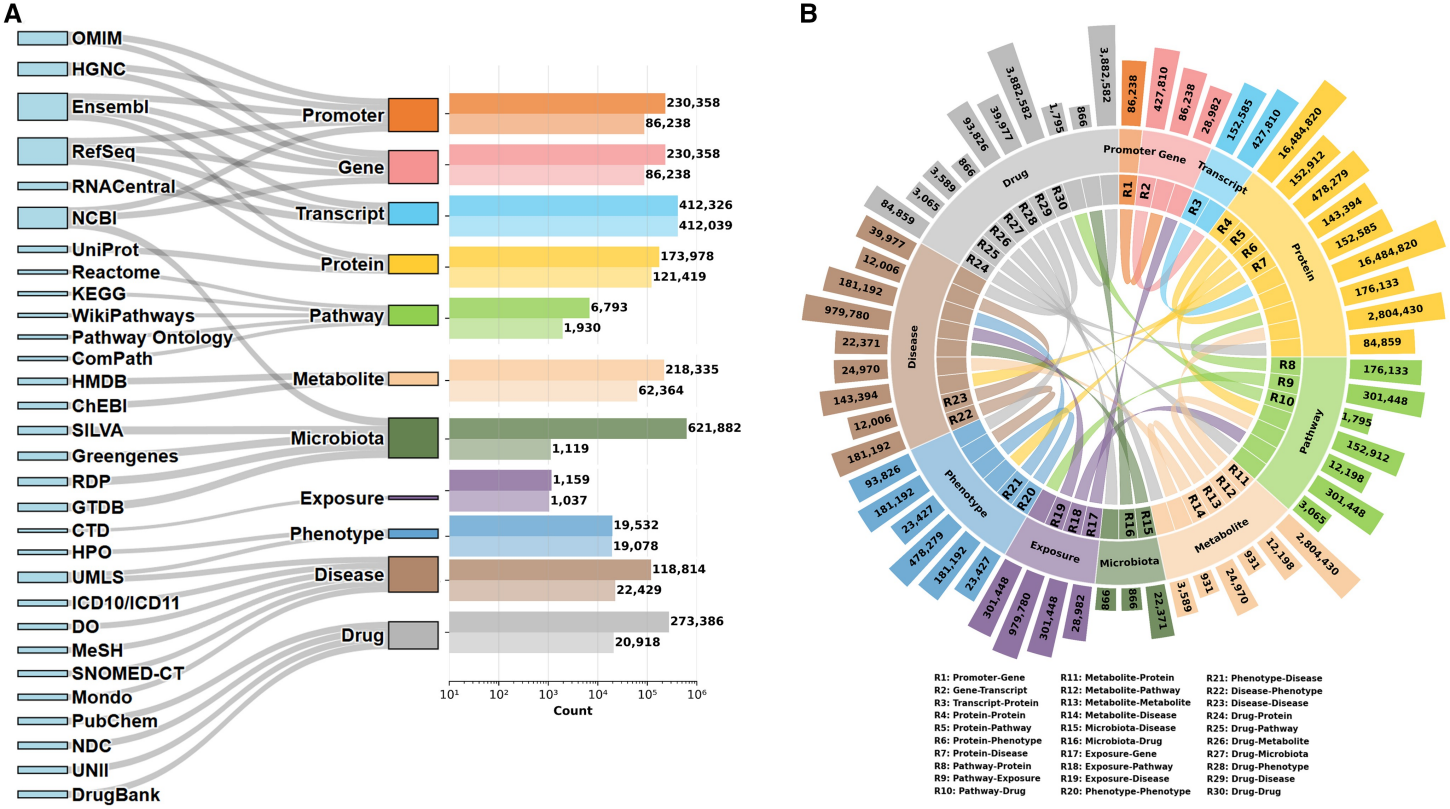
Overview of integrated biomedical entities and their relations in *BioMedGraphica*. (A) Data sources and entity distributions. The left panel shows the data sources (e.g. OMIM, HGNC, Ensembl, UniProt, KEGG, SILVA, DrugBank, etc.) used to define and harmonize 11 biomedical entity types: Promoter, Gene, Transcript, Protein, Pathway, Metabolite, Microbiota, Exposure, Phenotype, Disease, and Drug. The right panel presents the logarithmic-scaled bar plot showing the total number of unique entities in full BioMedGraphica (upper bar) and BioMedGraphica-Conn (lower opacity bar). (B) Circular chord diagram of pairwise relationships between biomedical entities. Each segment represents a specific entity type, and outer arcs quantify the total number of cross-entity edges for each type. The inner chords indicate the direction and volume of entity-to-entity relationships (e.g. Gene–Transcript, Protein–Pathway, Microbiota–Disease). Each relationship type is labeled (e.g. R1: Promoter–Gene, R2: Gene–Transcript, R10: Pathway–Drug, R23: Disease–Drug and total edge counts for selected relationships are annotated).

A wide range of reputable biomedical databases were utilized to gather and integrate various types of data related to genes, transcripts, proteins, and other biomedical entities (see Fig. 2). This comprehensive integration ensured data consistency and accuracy, creating a unified framework essential for research. As shown in Table 2, the total number of entries in the original data file from each respective database is listed. For the chemical entities of biological interest (ChEBI) database, we utilized two primary datasets: one provided the mapping between ChEBI IDs and their corresponding InChI, while the other contained the mapping between ChEBI IDs and another database. For the unique ingredient identifier (UNII) database, our source data was obtained from two sources: one from PubChem, and the other provided by the FDA. For SILVA, we selected the LSU and SSU datasets. In Table 2, we not only present the total number of entries after merging the two files but also indicate the total number of rows for each dataset in parentheses. Similarly, for genome taxonomy database (GTDB), we selected the data for both archaea and bacteria. The total number of entries after merging is indicated, with the individual row counts for each dataset provided in parentheses. Below is an expanded description of the databases used and the extracted data (see Table 2 and Table 1, available as supplementary data at *Bioinformatics* online, for details).

**Table 2:** Overview of entity databases.

Database names	Full names	Entity types	Total number of rows
Ensembl (Howe *et al.* 2021)	Ensembl	Gene	86 406
		Transcript	451 959
		Protein	157 628
OMIM (Amberger *et al.* 2009)	Online Mendelian Inheritance in Man	Gene	29 021
HGNC (Povey *et al.* 2001)	HUGO Gene Nomenclature Committee	Gene	43 916
NCBI (Schoch *et al.* 2020)	National Center for Biotechnology Information	Gene	193 340
		Microbiota	2 631 459
RefSeq (O’Leary *et al.* 2016)	NCBI—Reference Sequence Database	Gene	845 230
		Transcript	19 404
		Protein	376 561
RNACentral (Sweeney *et al.* 2019)	RNACentral	Transcript	66 789
UniProt (Wu *et al.* 2006)	Universal Protein Resource	Protein	20 417
Reactome (Fabregat *et al.* 2018)	Reactome	Pathway	2751
KEGG (Kanehisa and Goto 2000)	Kyoto Encyclopedia of Genes and Genomes	Pathway	365
WikiPathways (Kelder *et al.* 2012)	WikiPathways	Pathway	1534
Pathway Ontology (Petri *et al.* 2014)	Pathway Ontology	Pathway	2677
ComPath (Domingo-Fernández *et al.* 2018)	Comparative Pathology Platform of the University of Bern	Pathway	1592
HMDB (Wishart *et al.* 2022)	Human Metabolome Database	Metabolite	217 920
ChEBI (Degtyarenko *et al.* 2008)	Chemical Entities of Biological Interest	Metabolite	5757
			392 733
SILVA (Quast *et al.* 2013)	SILVA	Microbiota	2 214 227(227 318; 2 224 690)
Greengenes (DeSantis *et al.* 2006)	Greengenes	Microbiota	1 144 866
RDP (Cole *et al.* 2014)	Ribosomal Database Project	Microbiota	10 302
GTDB (Parks *et al.* 2018)	Genome Taxonomy Database	Microbiota	596 859(12 477; 584 382)
CTD (Davis *et al.* 2021)	The Comparative Toxicogenomics Database	Exposure	3539/224 304
HPO (Köhler *et al.* 2021)	Human Phenotype Ontology	Phenotype	19 533
UMLS (Bodenreider 2004)	Unified Medical Language System	Phenotype	14 036 386
		Disease	16 704 679
ICD10/ICD11 (World Health Organization 2004, 2018)	International Classification of Diseases	Disease	12 597/36 044
DO (Schriml *et al.* 2012)	Disease Ontology	Disease	38 212
MeSH (Lipscomb 2000)	Medical Subject Headings	Disease	5056
SNOMED-CT (Donnelly 2006)	Systematized Nomenclature of Medicine Clinical Terms	Disease	1 679 595
Mondo (Vasilevsky *et al.* 2020)	Mondo	Disease	134 286
PubChem (Wang *et al.* 2009)	Public Chemical Databases	Drug	123 357
NDC (Tribble 2025)	National Drug Code	Drug	107 980
UNII (Weisgerber 1997)	Unique Ingredient Identifier	Drug	159 376
			152 870
DrugBank (Knox *et al.* 2024)	DrugBank	Drug	17 430

#### 2.1.2 Relation database introduction and collection

This study integrates not only entity datasets but also a comprehensive range of relational datasets, facilitating the exploration of various biological and chemical interactions (see Fig. 2). These relational datasets capture complex relationships among genes, transcripts, proteins, drugs, diseases, phenotypes, pathways, metabolites, and microbiota, supporting advanced analyses in precision health (see overall data details in Table 3 and Table 2 in Section A, available as supplementary data at *Bioinformatics* online, for data collection details).

**Table 3:** General information about relation databases.

Database names	Full names	From	To	Edge types	Number of rows
Ensembl (Howe *et al.* 2021)	Ensembl	Gene	Transcript	Gene–Transcript	412 034
		Transcript	Protein	Transcript–Protein	412 034
RefSeq (O’Leary *et al.* 2016)	Reference Sequence Database	Gene	Transcript	Gene–Transcript	33 421
		Transcript	Protein	Transcript–Protein	30 207
UniProt (Wu *et al.* 2006)	Universal Protein Database	Transcript	Protein	Transcript–Protein	20 417
		Protein	Disease	Protein–Disease	20 417
BioGrid (Stark *et al.* 2006, Oughtred *et al.* 2019)	Biological General Repository for Interaction Datasets	Protein	Protein	Protein–Protein	942 241
STRING (Szklarczyk *et al.* 2015, 2019)	Search Tool for the Retrieval of Interacting Genes/Proteins	Protein	Protein	Protein–Protein	13 715 404
KEGG (Kanehisa and Goto 2000)	Kyoto Encyclopedia of Genes and Genomes	Protein	Protein	Protein–Protein	52 973
		Protein	Pathway	Protein–Pathway	21 051
		Metabolite	Pathway	Metabolite–Pathway	19 125
		Pathway	Protein	Pathway–Protein	24 475
		Pathway	Drug	Pathway–Drug	2334
		Drug	Pathway	Drug–Pathway	3922
HPO (Köhler *et al.* 2021)	Human Phenotype Ontology	Protein	Phenotype	Gene–Phenotype	316 718
		Protein	Disease	Gene–Disease	15 593
		Phenotype	Phenotype	Phenotype–Phenotype	19 533
		Phenotype	Disease	Phenotype–Disease	271 776
		Disease	Phenotype	Disease–Phenotype	271 776
DisGeNet (Piñero *et al.* 2016)	DisGeNet	Protein	Disease	Protein–Disease	91 484
DISEASES (Pletscher-Frankild *et al.* 2015)	DISEASES	Protein	Disease	Protein–Disease	96 763
HMDB (Wishart *et al.* 2022)	Human Metabolome Database	Metabolite	Protein	Metabolite–Protein	863 759
		Metabolite	Disease	Metabolite–Disease	27 670
		Drug	Metabolite	Drug–Metabolite	3258
STITCH	STITCH (‘search tool for interactions of chemicals’)	Metabolite	Protein	Metabolite–Protein	15 473 939
MetaNetX (Moretti *et al.* 2021)	MetaNetX	Metabolite	Metabolite	Metabolite–Metabolite	11 723
DisBiome (Janssens *et al.* 2018)	DisBiome	Microbiota	Disease	Microbiota–Disease	10 866
HMDAD	Human Microbe-Disease Association Database	Microbiota	Disease	Microbiota–Disease	483
MDAD (Sun *et al.* 2018)	Microbe-Drug Association Database	Microbiota	Drug	Microbiota–Drug	5055
		Drug	Microbiota	Drug–Microbiota	5055
PharmacoMicrobiomics (Doestzada *et al.* 2018)	PharmacoMicrobiomics	Microbiota	Drug	Microbiota–Drug	69
		Drug	Microbiota	Drug–Microbiota	69
CTD (Davis *et al.* 2021)	The Comparative Toxicogenomics Database	Pathway	Exposure	Pathway–Exposure	1 624 470
		Exposure	Gene	Exposure–Gene	2 892 ,325
		Exposure	Pathway	Exposure–Pathway	1 624 470
		Exposure	Disease	Exposure–Disease	9 329 083
DO (Schriml *et al.* 2012)	Disease Ontology	Disease	Disease	Disease–Disease	14 339
DrugBank (Knox *et al.* 2024)	DrugBank	Drug	Protein	Drug–Protein	26 245
		Drug	Drug	Drug–Drug	2 855 848
BindingDB (Gilson *et al.* 2016)	Binding Database	Drug	Protein	Drug–Protein	1 610 889
DrugCentral (Ursu *et al.* 2017)	DrugCentral	Drug	Protein	Drug–Protein	14 301
		Drug	Disease	Drug–Disease	42 307
SIDER (Kuhn *et al.* 2010)	Side Effect Resource	Drug	Phenotype	Drug–Phenotype	309 849

The last column represents the total number of rows in the original dataset from each database.

### 2.2 Harmonizing resources

As shown in Fig. 1, the integrated biomedical knowledge graph system, *BioMedGraphica*, has been proposed. By aggregating datasets from diverse sources, the system integrates 11 types of biological entities derived from 30 databases into a unified knowledge graph. Promoter entities are incorporated by mapping them to their corresponding gene entities, reflecting the regulatory influence of promoters on gene expression as assumed in the *BioMedGraphica* framework. Furthermore, relationships among these entities are established by harmonizing information from 22 relational databases, resulting in 30 distinct edge types. Detailed procedures for data merging and harmonization are described in the following sections.

#### 2.2.1 Entity integration

**Figure 3:**
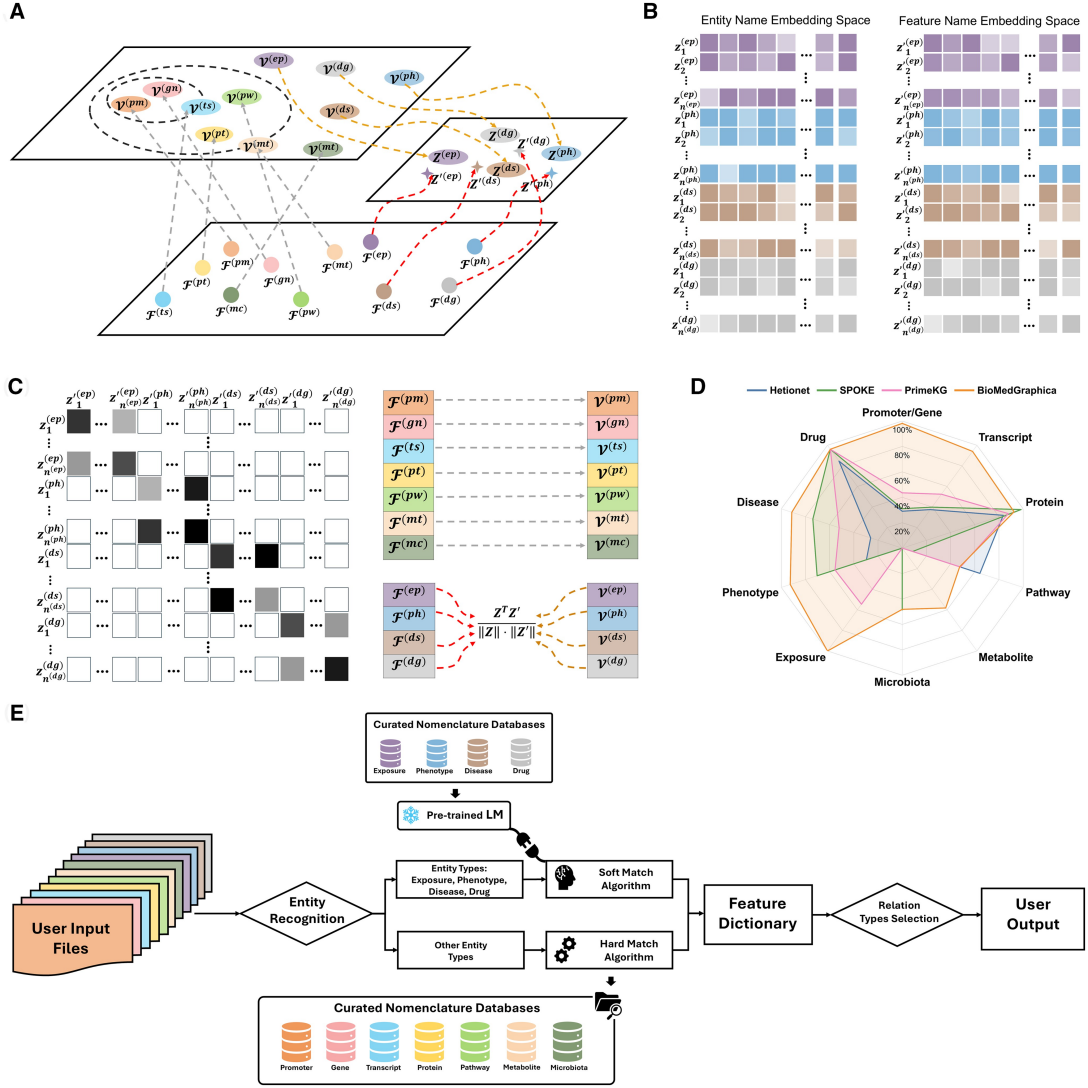
Pipeline of software *BioMedGraphica*. (A) Entity matching algorithms demonstration. Two strategies are used: a hard match that retrieves entities via standardized identifiers from curated nomenclature and a soft match that embeds both entity names and user-provided feature names into a shared representation space with a pretrained LM, selecting the highest-similarity candidate. (B) Schematic of embedding spaces for entity names and feature names, illustrating their vectorized representations by pretrained LM. (C) Cosine similarities yield a top k candidate set, and user confirmation finalizes a one-to-one mapping for each feature to generate mapping dictionary D. (D) Performance of entity matching across entity types on different platforms. (E) BioMedGraphica pipeline begins with user input files (with sample integration). For standardized-ID entities—promoter, gene, transcript, protein, pathway, metabolite, microbiota—BioMedGraphica performs hard matching against curated nomenclature. For free-text entities—exposure, phenotype, disease, drug—it applies soft matching with a pretrained LM for semantic alignment. Matched items are consolidated into a feature dictionary with contextual attributes; relation selection and autocompletion then produce structured outputs (TNG subsets). The workflow combines curated-ID precision with LM-based semantics to generate AI-ready biomedical graphs enriched with textual annotation.

**Figure 4:**
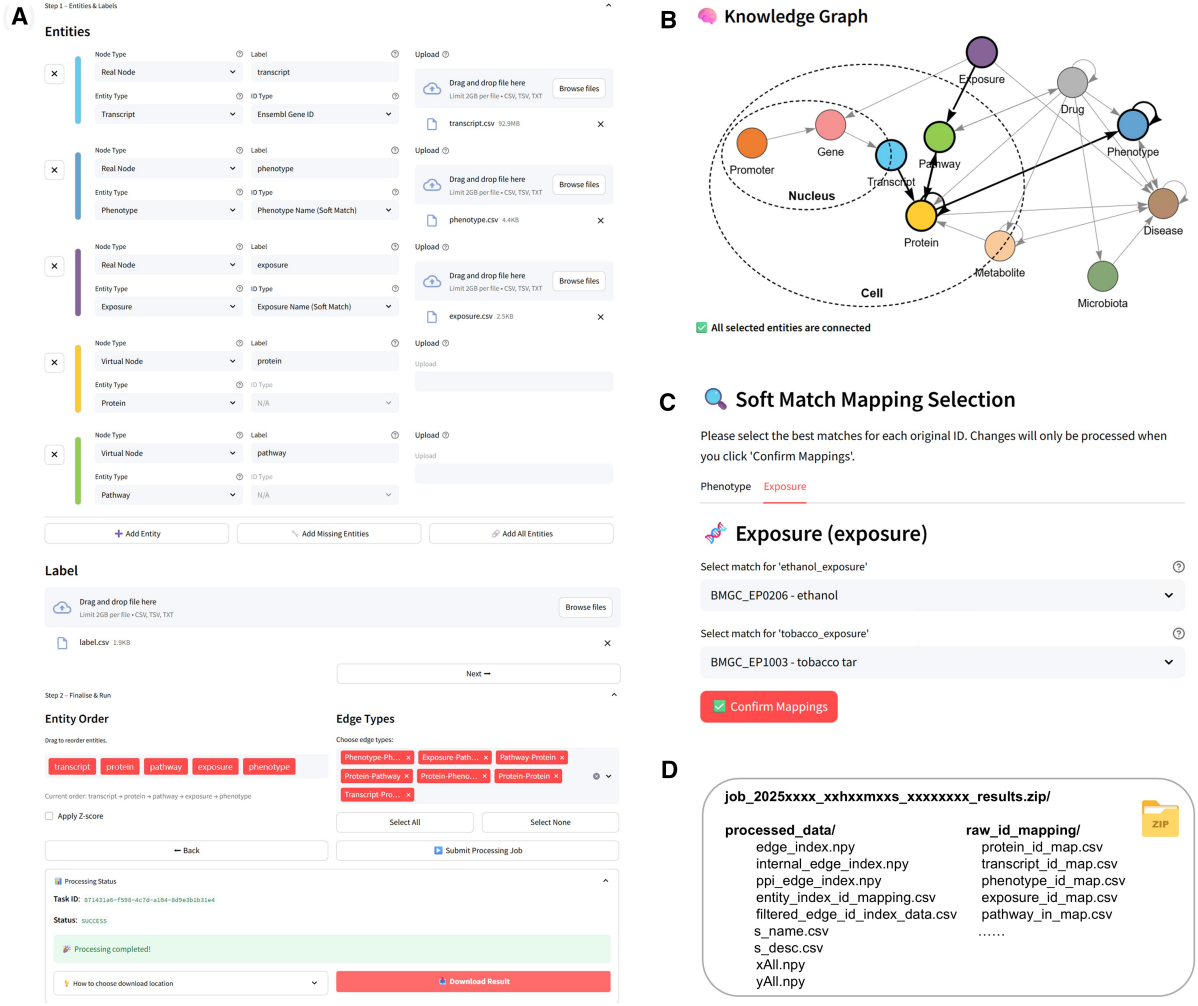
*BioMedGraphica* GUI and usage demonstration of the Emory_Vascular dataset. (A) Overview of the web-based user interface for uploading the four input biomedical files required for TNG generation. (B) The view of BioMedGraphica knowledge graph will highlight the selected entity types and relations based on the entity types integrated in this step. This panel also evaluates graph connectivity and marks missing entity types, which can be automatically completed via virtual entities if required. (C) The soft matching results interface, where candidate matches BioMedGraphica IDs are displayed, requiring user confirmation before proceeding. (D) Structure of the compressed output directory generated upon workflow completion, containing graph-ready feature matrices and entity-to-ID mapping files. For further details and instructions, please see the video demo from link: https://github.com/FuhaiLiAiLab/BioMedGraphica/blob/main/README.md.

To construct a unified biomedical knowledge base, comprehensive entity integration was performed across multiple biological domains. Given that discrepancies in entity names are a pervasive challenge in biomedical data integration, BioMedGraphica adopts an identifier-centric strategy that relies exclusively on authoritative cross-reference mappings provided by source databases, rather than on manual name harmonization or heuristic merging. This design aligns with prior large-scale biomedical knowledge graph platforms, including the Monarch Initiative (Putman *et al.* 2024), Petagraph (Stear *et al.* 2024), and CROssBAR (Doğan *et al.* 2021), which similarly emphasize identifier-driven integration anchored in curated cross-references. Integration is conducted through shared-field matching and incremental merging steps, with identifier selection determined locally by the resources being combined and by the resolution of available linking fields. For gene entities, datasets from Ensembl, HGNC, and NCBI were first merged based on Ensembl IDs, followed by incorporation of RefSeq and OMIM data using NCBI Gene IDs as the primary unifying identifier. Transcript entities were integrated by adopting the Ensembl Transcript Stable ID as the standard reference, with descriptions retrieved through the Ensembl BioMart API. Protein entities were merged by aligning Ensembl and UniProt records via Protein Stable ID Versions, followed by the incorporation of RefSeq mappings, with annotations obtained from UniProt. Pathway entities were integrated using Pathway Ontology as the foundational framework, supplemented by KEGG, Reactome, and WikiPathway datasets through equivalent mappings facilitated by ComPath. For metabolite entities, ChEBI IDs were initially used for alignment, with HMDB IDs ultimately established as the minimal granularity unit. Microbiota data were harmonized using NCBI Taxon IDs to ensure consistency across datasets. Exposure entities were unified based on CAS numbers, leveraging their broad availability across relevant databases. Phenotype integration was initiated from HPO terms, with systematic cleaning of labels to remove generic descriptors and consolidation based on refined labels linked to HPO identifiers. Disease entities were integrated through a multistep mapping strategy involving UMLS, MeSH, SNOMED-CT, ICD-10, ICD-11, Disease Ontology, and Mondo, with UMLS IDs designated as the minimal unit of granularity. Finally, drug entities were merged by aligning NDC and UNII datasets using substance names, followed by incorporation of PubChem, CAS, ChEBI, and DrugBank information through mapped identifiers, establishing CAS numbers as the primary reference. Throughout the integration process, database merging was anchored by bolded columns in the supplementary tables, ensuring the uniqueness of key identifiers, with detailed workflows and results documented in Figs 1–10 and Tables 3–12, available as supplementary data at *Bioinformatics* online.

#### 2.2.2 Relation integration

The construction of edges utilized data from 22 distinct databases, mapping raw database IDs to their corresponding BioMedGraphica IDs to form relationships. A notable challenge arose from one-to-many mappings, where a single database ID, such as 614 807 (OMIM ID), corresponds to multiple BioMedGraphica IDs (BMG_DS065861 and BMG_DS080589), due to the one-to-many relationships between OMIM databases and other databases. Aside from this, all relationships were directional and presented in a From-To format. To address bidirectional relationships, two distinct methodologies were employed. The first involved reversing the direction of the relationship. For instance, while protein–protein interactions are intrinsically bidirectional, the original dataset lacked explicit directionality. To resolve this, a reversed copy of the data was generated, merged with the original dataset, and duplicates were subsequently eliminated. The second approach entailed establishing new relationships where reversal was inappropriate. For example, in disease–phenotype associations, reversing the data alone was insufficient; instead, a complementary phenotype-to-disease relationship was created to accurately represent the connection. The edge structure was meticulously designed to conform to a one-to-one mapping framework, ensuring that each instance of one database ID mapping to multiple BioMedGraphica IDs results in the generation of distinct edges. This strategy significantly amplified the total number of edges, exceeding a straightforward summation of interdatabase relationships due to the one-to-many nature of the mappings (see Table 4 for details).

**Table 4:** Harmonized relations information.

Interaction type	Database	Initial edge number	Matching		Total
			Unique	Total	
Gene–Transcript	Ensembl	412 034	412 034	427 393	427 810
	RefSeq	33 421	6625	7001	
Transcript–Protein	Ensembl	412 034	123 845	123 858	152 585
	Uniprot	50 765	50 765	50 765	
	RefSeq	30 207	6868	35 575	
Protein–Protein	BioGrid	1 690 122	1 689 574	1 689 574	16 484 820
	STRING	13 715 404	13 285 010	13 193 859	
	KEGG	52 973	51 971	1 967 514	
Protein–Pathway	KEGG	21 051	20 867	152 912	152 912
Protein–Phenotype	HPO	259 118	255 171	478 279	478 279
Protein–Disease	UniProt	4821	4784	5573	143 394
	DISEASES	69 896	11 108	12 387	
	HPO	7482	7184	15 444	
	DisGeNet	91 484	70 968	129 750	
Pathway–Protein	KEGG	24 475	24 368	176 133	176 133
Pathway–Drug	KEGG	2334	1331	1795	1795
Pathway–Exposure	CTD	1 624 470	305 427	301 448	301 448
Metabolite–Protein	HMDB	863 759	849 980	849 993	2 804 430
	STITCH	74 576 305	1 973 337	1 975 413	
Metabolite–Pathway	KEGG	19 622	7208	12 198	12 198
Metabolite–Metabolite	MetaNetX	23 711	886	931	931
Metabolite–Disease	HMDB	24 755	24 669	24 970	24 970
Microbiota–Disease	DisBiome	8438	3521	4362	22 371
	HMDAD	450	21 663	18 014	
Microbiota–Drug	MDAD	5055	2078	816	866
	PharmacoMicrobiomics	69	68	67	
Exposure–Gene	CTD	42 261	28 982	323 906	323 ,906
Exposure–Pathway	CTD	1 624 470	305 427	301 448	301 448
Exposure–Disease	CTD	9 329 083	679 195	979 780	979 780
Phenotype–Phenotype	HPO	23 461	23 427	23 427	23 427
Phenotype–Disease	HPO	155 989	155 063	181 192	181 192
Disease–Phenotype	HPO	155 989	155 063	181 192	181 192
Disease–Disease	DO	11 836	9747	12 006	12 006
Drug–Protein	DrugBank	25 670	20 865	23 100	84 859
	BindingDB	1 161 440	58 858	58 858	
	DrugCentral	14 301	13 427	15 107	
Drug–-Pathway	KEGG	3922	2380	6235	6235
Drug–Metabolite	HMDB	3258	3171	3065	3065
Drug–Microbiota	MDAD	5055	2078	805	866
	PharmacoMicrobiomics	69	68	67	
Drug–Phenotype	SIDER	152 759	91 692	93 826	93 826
Drug–Disease	DrugCentral	50 011	35 940	39 977	39 977
Drug–Drug	DrugBank	2 855 848	2 845 794	3 882 582	3 882 582

## 3 Results

### 3.1 BioMedGraphica: an integrative textual biomedical prior knowledge graph

For entity integration, the promoter entity was added to the entity by copying gene entity based on the assumption that each gene was influenced by a corresponding promoter. Therefore, the database for *BioMedGraphica* includes 11 entity types and 30 edge types, comprising 2 306 921 entities and 27 232 091 relations, composing the knowledge graph G=(V, E) and 834 809 entities and 27 087 971 relations, composing the connected knowledge graph $G_{c}=(V_{c},\;E_{c})$ (see Tables 5 and 6 for number of each entity and edge type). Beyond structural knowledge integration, each entity is further enriched with comprehensive textual annotations, denoted as T and $T_{c}$ for the full graph G and its connected component $G_{c}$, respectively. These include nomenclature records harmonized across multiple biomedical resources, capturing alternative identifiers, synonyms, and cross-references that resolve inconsistencies across databases. In addition, descriptive metadata provides functional insights, mechanistic roles, and biological contexts, such as molecular activities, pathway involvement, disease associations, and therapeutic relevance (details are documented in Figs 1–10, available as supplementary data at *Bioinformatics* online). By combining nomenclature harmonization with descriptive annotations, the textual knowledge graph not only ensures consistent entity recognition but also provides a semantically rich layer of biomedical knowledge that facilitates graph–text fusion, interpretability, and downstream AI applications.

**Table 5:** Summarized entity information.

Entity type	Math annotation	BMG^a^ count	BMG percentage (%)	BMGC^a^ count	BMGC percentage (%)	BMGC in BMG (%)
Promoter	$V^{\left(pm\right)}$	230 358	9.9855	86 238	10.3303	37.4365
Gene	$V^{\left(gn\right)}$	230 358	9.9855	86 238	10.3303	37.4365
Transcript	$V^{\left(ts\right)}$	412 326	17.8734	412 039	49.3573	99.9304
Protein	$V^{\left(pt\right)}$	173 978	7.5416	121 419	14.5445	69.7899
Pathway	$V^{\left(pw\right)}$	6793	0.2945	1930	0.2312	28.4116
Metabolite	$V^{\left(mt\right)}$	218 335	9.4643	62 364	7.4705	28.5634
Microbiota	$V^{\left(mc\right)}$	621 882	26.9572	1119	0.1340	0.1799
Exposure	$V^{\left(ep\right)}$	1159	0.0502	1037	0.1242	89.4737
Phenotype	$V^{\left(ph\right)}$	19 532	0.8467	19 078	2.2853	97.6756
Disease	$V^{\left(ds\right)}$	118 814	5.1503	22 429	2.6867	18.8774
Drug	$V^{\left(dg\right)}$	273 386	11.8507	20 918	2.5057	7.6515
Total	V	2 306 921	100	834 809	100	36.1872

a BMG shorts for *BioMedGraphica*, and BMGC shorts for *BioMedGraphica-Conn*, which stands for the connected knowledge graph by removing the isolated nodes in *BioMedGraphica*.

**Table 6:** Summarized information of relation types.

Relation type	Math annotation	Count	Percentage
Promoter–Gene^a^	$E^{\left(pm-gn\right)}$	230 358/86 238	0.8459/0.3184
Gene–Transcript	$E^{\left(gn-ts\right)}$	427 810	1.5710
Transcript–Protein	$E^{\left(ts-pt\right)}$	152 585	0.5603
Protein–Protein	$E^{\left(pt-pt\right)}$	16 484 820	60.5345
Protein–Pathway	$E^{\left(pt-pw\right)}$	152 912	0.5615
Protein–Phenotype	$E^{\left(pt-ph\right)}$	478 279	1.7563
Protein–Disease	$E^{\left(pt-ds\right)}$	143 394	0.5266
Pathway–Protein	$E^{\left(pw-pt\right)}$	176 133	0.6468
Pathway–Exposure	$E^{\left(pw-ep\right)}$	301 448	1.1070
Pathway–Drug	$E^{\left(pw-dg\right)}$	1795	0.0066
Metabolite–Protein	$E^{\left(mt-pt\right)}$	2 804 430	10.2982
Metabolite–Pathway	$E^{\left(mt-pw\right)}$	12 198	0.0447
Metabolite–Metabolite	$E^{\left(mt-mt\right)}$	931	0.0034
Metabolite–Disease	$E^{\left(mt-ds\right)}$	24 970	0.0916
Microbiota–Disease	$E^{\left(mc-ds\right)}$	22 371	0.0821
Microbiota–Drug	$E^{\left(mc-dg\right)}$	866	0.0032
Exposure–Gene	$E^{\left(ep-gn\right)}$	28 982	0.1064
Exposure–Pathway	$E^{\left(ep-pw\right)}$	301 448	1.1070
Exposure–Disease	$E^{\left(ep-ds\right)}$	979 780	3.5979
Phenotype–Phenotype	$E^{\left(ph-ph\right)}$	23 427	0.0860
Phenotype–Disease	$E^{\left(ph-ds\right)}$	181 192	0.6654
Disease–Phenotype	$E^{\left(ds-ph\right)}$	181 192	0.6654
Disease–Disease	$E^{\left(ds-ds\right)}$	12 006	0.0441
Drug–Protein	$E^{\left(dg-pt\right)}$	84 859	0.3116
Drug–Pathway	$E^{\left(dg-pw\right)}$	3065	0.0113
Drug–Metabolite	$E^{\left(dg-mt\right)}$	3589	0.0132
Drug–Microbiota	$E^{\left(dg-mc\right)}$	866	0.0032
Drug–Phenotype	$E^{\left(dg-ph\right)}$	93 826	0.3445
Drug–Disease	$E^{\left(dg-ds\right)}$	39 977	0.1468
Drug–Drug	$E^{\left(dg-dg\right)}$	3 882 582	14.2574
Total	E	27 232 091/27 087 971	100

a For Promoter–Gene relation, the column count demonstrates the number of relations in BMG and BMGC respectively, due to that Promoter–Gene relations are virtual relation generated automatically.

### 3.2 Tool for TNG data generation

For entity integration, the promoter entity was added to the entity by copying gene entity based on the assumption BioMedGraphica is a unified software platform designed to integrate heterogeneous biomedical datasets with a structured knowledge graph and affiliated textual annotations, enabling coherent representation for graph foundation models. By bridging data ranging from omic data to clinical data with curated graph-based knowledge, the tool provides a scalable framework for constructing TNG subsets tailored to user inputs. As shown in Fig. 3, user can input the files into the software, which are denoted as $X=\{X^{\left(pm\right)},X^{\left(gn\right)},X^{\left(ts\right)},X^{\left(pt\right)},\;X^{\left(pw\right)},\;X^{\left(mt\right)},X^{\left(mc\right)},\;X^{\left(ep\right)},X^{\left(ph\right)},\;X^{\left(ds\right)},\;X^{\left(dg\right)}\}$, where $X^{\left(e\right)}\in R^{n^{\left(e\right)}\times |F^{\left(e\right)}|}$ and e denotes one of the 11 entity types mentioned above, $n_{e}$ stands for the number of samples, $F^{\left(e\right)}$represents features set of the entity type e. Once the files are imported, sample sizes across entity types are aligned to $n^{\left(\mathrm{in}\right)}$, defined as the intersection of all inputs. This alignment yields a unified matrix $X^{\left(\mathrm{in}\right)}\in R^{n^{\left(\mathrm{in}\right)}\times |F^{\left(\mathrm{in}\right)}|}$, and $|F^{\left(\mathrm{in}\right)}|=\sum_{e}{|F}^{\left(e\right)}|$, which can be viewed as a consolidated representation of the user-provided data. To ensure semantic coherence and enable effective inference, BioMedGraphica employs the BioMedGraphica-Conn, $G_{c}$, as a solid knowledge graph foundation, which removes isolated nodes and retains only the largest connected subgraph. This strategy is consistent with graph theory principles that emphasize the importance of connectivity for traversal and reasoning and aligns with prior work showing that connected knowledge graphs enhance AI-driven interpretability and mechanistic discovery (Guo *et al.* 2024, Rajabi and Etminani 2024, Ma *et al.* 2025). Hence, by matching the features $F^{\left(\mathrm{in}\right)}$ with entities V existing in knowledge graph$\;G_{c}$, the entities will be formed with $V^{\left(\mathrm{in}\right)}$ and mapping function $M:F^{\left(\mathrm{in}\right)}\to V^{\left(\mathrm{in}\right)}$, which is curated in python dictionary format.

In addition, users may incorporate virtual entities that function as essential intermediates in biological processes (e.g. transcripts in the gene–transcript–protein chain), thereby producing a refined entity set $V^{\left(\mathrm{sub}\right)}$. Users may also specify the relation types to include, yielding a subgraph $G^{\left(\mathrm{sub}\right)}=(V^{\left(\mathrm{sub}\right)},E^{\left(\mathrm{sub}\right)})$. Together with the matched and auto-completed (virtual) entities, a descriptive feature matrix $T^{\left(\mathrm{sub}\right)}$ ($T^{\left(\mathrm{sub}\right)}\in R$), is generated, representing the associated textual annotations. This process results in an aligned feature space of size${|V}^{\left(\mathrm{sub}\right)}|$, forming a new feature matrix $X^{\left(\mathrm{sub}\right)}\in R^{n^{\left(\mathrm{in}\right)}\times {|V}^{\left(\mathrm{sub}\right)}|}$. Finally, the complete TNG user-specific subset is produced as $D^{\left(\mathrm{sub}\right)}=\{X^{\left(\mathrm{sub}\right)},\;G^{\left(\mathrm{sub}\right)},\;T^{\left(\mathrm{sub}\right)}\}$. The subsequent section elaborates on the procedures for entity matching and relation construction.

#### 3.2.1 Entity recognition

The entities from the input files will be recognized by either hard matching algorithm or soft matching algorithm. For most entity types including promoter, gene, transcript, protein, pathway, metabolite and microbiota, uniformed IDs are used as matching symbols. These entities can therefore be applied with the hard matching algorithm by aligning them with IDs collected from various resources included in the BioMedGraphica. However, for the entity types with flexibility to name them with self-definition, including exposure, phenotype, disease and drug, they should be applied with the soft matching algorithm.

When matching the features input by users to the existing entities in the BioMedGraphica knowledge base, the specially designed algorithm using a pretrained BioBERT model was leveraged for disease, phenotype, drug and exposure entities, which allows for the comparison of disease, phenotype, drug and exposure terms based on their semantic similarity for building the mapping dictionary D. Textual embeddings were obtained from the final hidden layer using mean pooling over nonpadding tokens followed by L2 normalization, and similarity was computed via cosine similarity implemented with FAISS-based inner-product search (detailed model configurations and implementation settings are described in the Section C.1, available as supplementary data at *Bioinformatics* online). Then, the similarity score will be calculated between a given query feature name, $f_{\mathrm{name}}\;$(f is the corresponded entity), and precomputed entity embeddings by scoring function S with


(1) S(f)=LM(fname)T LM(Vname)‖LM(fname)‖⋅‖LM(Vname)‖,#

where $f_{\mathrm{name}}$ ($f_{\mathrm{name}}\in F_{\mathrm{name}}^{\left(\mathrm{in}\right)}$) is the queried feature name from the unified user input file, $F_{\mathrm{name}}^{\left(\mathrm{in}\right)}$ ($F_{\mathrm{name}}^{\left(\mathrm{in}\right)}\in R^{\left|F^{\left(\mathrm{in}\right)}\right|}$) and$\;V_{\mathrm{name}}$ ($V_{\mathrm{name}}\in R^{\left|V\right|}$) is the corresponding entity names of $F^{\left(\mathrm{in}\right)}$ and V, and pretrained BioBERT language model is denoted as LM. In detail, the model will process exposure, phenotype, drug and disease entities in *BioMedGraphica* by


(2) z=LM(vname),

where $v_{\mathrm{name}}$ ($v_{\mathrm{name}}\in V_{\mathrm{name}}$) is entity name and z ($z\in R^{d}$) denotes the transformed embedding space for $v_{\mathrm{name}}$. Similarly, the queried feature name will be embedded by


(3) z′=LM(fname),

where z' ($z'\in R^{d}$) denotes the transformed embedding space for $f_{\mathrm{name}}$. Afterward, the top k most similar entities will be extracted by


(4) Vk(f)=I[argmaxk[S(f)]],

where ${\mathrm{argmax}}_{k}$ can identify top k most similar entity names $V_{k}^{\left(f\right)}$ ($V^{\left(f\right)}\in R^{k}$) and I(⋅) is the one-to-one mapping function which will map the entity names to entities in *BioMedGraphica*. In these top k most similar entity, the user will define only one entity, $V^{\left(f\right)}$, to be matched for the queried feature name $f_{\mathrm{name}}$. For other entity types, the hard match method was leveraged to search for exact entity name for the queried feature name $f_{\mathrm{name}}$ with $V^{\left(f\right)}$. With this, the dictionary function M will be generated.

To evaluate the matching algorithm performance on our platform, we conducted a comparison across all entity types and benchmarked BioMedGraphica against Hetionet, SPOKE, and PrimeKG. As shown in Fig. 3D and Table 13, available as supplementary data at *Bioinformatics* online, BioMedGraphica achieves consistently strong performance across both hard- and soft-matching scenarios on most of entity matching tasks. For standardized identifier-based entities such as promoter/gene and transcript, the hard-matching procedure attains high alignment accuracy (e.g. 98.14% and 94.06%, respectively). For semantically complex entities handled by soft matching, including exposure, phenotype, disease and drug, the BioBERT-based approach also demonstrates robust performance. Detailed results and dataset descriptions are provided in Tables 14–24, available as supplementary data at *Bioinformatics* online.

#### 3.2.2 Relation/knowledge graph construction

By extracting the corresponding entities $V^{\left(\mathrm{in}\right)}$ of the input features $F^{\left(\mathrm{in}\right)}$ from the connected knowledge graph $G_{c}$, users can select the edge types annotated in Table 5 to construct the $E^{\left(\mathrm{in}\right)}$. To ensure the connectivity of the constructed subgraph, we designed a shortest-path-based connectivity assessment and autocompletion strategy. Specifically, if certain downstream nodes are missing, they are labeled as candidate entities for supplementation and corresponding virtual node suggestions are generated. The core connection, defined as $E^{\left(\mathrm{core}\right)}=\{E^{\left(pm-gn\right)},\;E^{\left(gn-ts\right)},\;E^{\left(ts-pt\right)}\}$, which serves as the backbone for connectivity analysis and the set of nodes is denoted as $V^{\left(\mathrm{core}\right)}=\{V^{\left(pm\right)},\;V^{\left(gn\right)},\;V^{\left(ts\right)},\;V^{\left(pt\right)}\}.$

To alleviate computational complexity, entity and relation types are generalized into coarse-grained categories within the BioMedGraphica abstract knowledge graph (Fig. 1). This abstraction yields an abstract graph $G_{\mathrm{abs}}=(V_{\mathrm{abs}},E_{\mathrm{abs}})$, consisting of 11 nodes and 30 edges, each corresponding to the entity and relation types of the underlying concrete knowledge graph. Based on the abstract knowledge graph $G_{\mathrm{abs}}$, we constructed an undirected knowledge graph $G_{\mathrm{abs}}^{\prime }=(V_{\mathrm{abs}},E_{\mathrm{abs}}^{\prime })$ for algorithm development. Within this framework, the abstract core connection set is denoted as ${E^{\prime }}_{\mathrm{abs}}^{\left(\mathrm{core}\right)}$ with its corresponding node set $V_{\mathrm{abs}}^{\left(\mathrm{core}\right)}$, while the abstract input connection set and its associated nodes are represented as ${E^{\prime }}_{\mathrm{abs}}^{\left(\mathrm{in}\right)}$ and $V_{\mathrm{abs}}^{\left(\mathrm{in}\right)}$, respectively, defining the input-specific graph as ${G^{\prime }}_{\mathrm{abs}}^{\left(\mathrm{in}\right)}$.

*Connectivity criterion and path evaluation.* To explicitly evaluate connectivity, the user-selected abstract entity types are examined on the undirected abstract schema, where each relation is treated as one unweighted hop. For each required node pair $\left(v_{\mathrm{start}},v_{\mathrm{end}}\right)$, the hop-based shortest distance is computed using Breadth-First Search (BFS), and all shortest paths $\Pi (v_{\mathrm{start}},v_{\mathrm{end}})$ are enumerated under a hop limit $h_{\max}$. For each candidate path $\pi_{p}\in \Pi (v_{\mathrm{start}},v_{\mathrm{end}})$, the missing-node set is defined as the set of nodes on π that appear on the path but are absent from the current input and are not already included in the core autocompletion set. If at least one shortest path contains no missing nodes, the node pair is considered connected. Otherwise, the algorithm retains the shortest paths that require the fewest missing nodes, and the union of their missing nodes is collected as the candidate set for supplementation.

*Core-chain completion strategy.* When the input intersects the ordered core chain, $V^{\left(\mathrm{core}\right)}$, the system first identifies the selected core entity type that is closest to the end of the chain (protein). After the core chain has been completed, noncore entity types are preferentially connected to this chain. This strategy reduces the number of pairwise shortest path computations required during the connectivity evaluation. The graph is considered connected only when the aggregated missing node set becomes empty. If missing nodes remain, additional candidate entity types are iteratively suggested until the connectivity requirement is satisfied. The detailed procedure is summarized in Algorithm 1.

Algorithm 1 Connectivity verification of abstract knowledge graph, ${G^{\prime }}_{\mathrm{abs}}^{\left(\mathrm{in}\right)}$

Input Entity set $V_{\mathrm{abs}}^{\left(\mathrm{in}\right)}$, edge set $E_{\mathrm{abs}}^{\left(\mathrm{core}\right)}$, max hop $h_{\max}$

Output Connectivity status (True/False), missing nodes set $V_{\Delta }$

Step 1:  Construct the undirected graph $G_{\mathrm{abs}}^{\prime }$ from $G_{\mathrm{abs}}$

Step 2.1:  If $V_{\mathrm{abs}}^{\left(\mathrm{in}\right)}\cap \{v_{\mathrm{abs}}^{\left(\mathrm{pm}\right)},\;v_{\mathrm{abs}}^{\left(\mathrm{gn}\right)},\;v_{\mathrm{abs}}^{\left(\mathrm{ts}\right)}\}\neq \;\emptyset :$

      Set a core connection chain subset ${E^{\prime }}_{\mathrm{sub}}^{\left(\mathrm{core}\right)}\subseteq \;{E^{\prime }}_{\mathrm{abs}}^{\left(\mathrm{core}\right)}$up to protein $v^{\left(\mathrm{pt}\right)}$, and its node set $V_{\mathrm{sub}}^{\left(\mathrm{core}\right)}\subseteq \;V_{\mathrm{abs}}^{\left(\mathrm{core}\right)}$

Step 2.2:  For each non-core selected entity $v_{i}^{\left(\epsilon \right)}\in V_{\mathrm{abs}}^{\left(\mathrm{in}\right)}\backslash V_{\mathrm{abs}}^{\left(\mathrm{core}\right)}$:

      If $\exists v_{j}^{\left(\sigma \right)}\in V_{\mathrm{abs}}^{\left(\mathrm{core}\right)}\;$such that $E^{\left(v_{i}^{\left(\epsilon \right)}\;-v_{j}^{\left(\sigma \right)}\right)}\in E_{\mathrm{abs}}^{\prime }:$

       Define path $\pi^{\left(\sigma \right)}=\;{E^{\prime }}_{\mathrm{sub}}^{\left(\mathrm{core}\right)}\;\cup E^{\left(v_{i}^{\left(\epsilon \right)}\;-v_{j}^{\left(\sigma \right)}\right)}$

       Mark missing nodes as $V_{\Delta }(\pi^{\left(\sigma \right)})=\;(V_{\mathrm{sub}}^{\left(\mathrm{core}\right)}\backslash V_{\mathrm{abs}}^{\left(\mathrm{in}\right)})\;\cup v_{j}^{\left(\sigma \right)}$

Step 3.1:   For each unordered pair $\left(v_{\mathrm{start}},v_{\mathrm{end}}\right)$ not directly attached to the core chain, or meet the condition that no core node is included

       Retrieve shortest paths $\Pi (v_{\mathrm{start}},v_{\mathrm{end}})$ in $G_{\mathrm{abs}}^{\prime }$ each with hop limit $h_{\max}$

Step 3.2:    For each path $\pi_{p}\in \Pi (v_{\mathrm{start}},v_{\mathrm{end}})$:

       Compute the missing nodes set $V_{\Delta }(\pi_{p})=\{\;v_{q}\in \;\pi_{p}\;|\;v_{q}\notin V_{\mathrm{abs}}^{\left(\mathrm{in}\right)}\;\}$

Step 3.3:    If ${\exists \pi }_{p}$ with $|V_{\Delta }(\pi_{p})|=0$, mark the pair as connected and continue

Step 3.4:   Else, choose $\Pi^{*}=\arg\underset{\pi_{p}}{\min}\left|V_{\Delta }(\pi_{p})\right|$ and record its nodes as the $V_{\Delta }(\Pi^{*})$

Step 4.1:   Set missing nodes $V_{\Delta }=\;V_{\Delta }(\pi^{\left(\sigma \right)})\;\cup \;V_{\Delta }(\Pi^{*})$

Step 4.2:   Set connectivity status as True, if missing nodes $V_{\Delta }=\emptyset$

Step 4.3:   Return connectivity status, missing nodes $V_{\Delta }$

Step 5:    If user accepts any supplementation nodes $V_{\Delta }^{+}\subseteq \;V_{\Delta }(\Pi^{*})$:

       Update $V^{\left(\mathrm{in}\right)}\leftarrow V^{\left(\mathrm{in}\right)}\cup V_{\Delta }^{+}$

       Re-run algorithm from Step 2

#### 3.2.3 Entities/relations matching accuracy

The evaluation of entity and relation matching demonstrates a clear dichotomy between deterministic hard matching and probabilistic soft matching. Hard matching yields near-perfect accuracy for entities standardized under controlled vocabularies (e.g. promoter, gene, transcript, protein, etc.), confirming the robustness of BioMedGraphica’s curated nomenclature integration. In contrast, soft matching powered by pretrained language models significantly enhances recognition for less standardized entities (e.g. phenotype, disease, exposure, drug), which enables automated semantic alignment and substantially improves efficiency over manual mapping, serving as a practical complement to hard matching for broader entity coverage. Nonetheless, current LLMs exhibit nontrivial error rates in multiple scenarios, including critical misassignments such as mapping *NC_000019.10* (RefSeq ID) to an incorrect Ensembl ID (*ENSG00000272512*). More concerning is their instability in relation extraction: for example, ChatGPT-5 erroneously linked the phenotype *Leukocytosis* to a misidentified drug with CAS number *106-60-5*, introducing fabricated relations inconsistent with biomedical ground truth. These results underscore both the strengths and limitations of generic language models, highlighting the need for BioMedGraphica’s hybrid strategy, which combines deterministic hard matches for structured entities with carefully constrained LM-driven soft matches, thereby ensuring reproducibility, accuracy, and trustworthiness in knowledge graph construction and downstream AI tasks.

#### 3.2.4 GUI design

The GUI was developed to enhance accessibility and usability, enabling researchers without extensive programming expertise to efficiently construct TNG subsets through an intuitive, guided workflow. By lowering the technical barrier, the interface facilitates broader adoption of BioMedGraphica across interdisciplinary biomedical communities (see Fig. 4 for an overview of BioMedGraphica GUI). To achieve this, the GUI implements a stepwise design that systematically guides users from data input to final output, ensuring both transparency and reproducibility in the processing pipeline. In detail, the GUI was developed to streamline the workflow of data input, recognition, filtering, and output generation. The interface begins with a user input module that supports both file upload and manual entry. Upon submission, the system performs automated data recognition and displays the inferred format in a preview pane for user validation. Users are then provided with options to refine the recognition type via dropdown menus or radio buttons (e.g. Entity Type A, Entity Type B), thereby enabling precise specification when necessary. Once confirmed, the workflow transitions to the entity-matching stage, where input records are aligned with the BioMedGraphica ID system. Only validated entities are retained for subsequent procedures. Users may further refine their datasets by selecting relational entities from curated databases (e.g. Relation Database 1, Relation Database 2), with the system reminding users to include essential intermediate entities and relations to support automated completion. After filters are applied, the system generates the processed TNG, which can be downloaded or visualized in a structured format. Overall, the GUI ensures usability and accessibility by guiding users through each stage of processing with intuitive controls, real-time validation, and clear instructions. The application is publicly accessible at https://app.biomedgraphica.org.

## 4 Data access and usage demonstration

### 4.1 BioMedGraphica knowledge graph data access

We provide two levels of data accessibility to support diverse user needs. First, the raw datasets are made available with download links, accompanied by detailed processing instructions in Sections A and B, available as supplementary data at *Bioinformatics* online, and open-source code hosted in our GitHub. These resources allow researchers to fully reproduce the data harmonization pipeline and customize entity and relation extraction according to their own requirements. Second, for users seeking ready-to-use resources, we also provide the processed and integrated BioMedGraphica knowledge graph through the Huggingface dataset, enabling seamless adoption in downstream computational pipelines. To facilitate adoption across communities with varying technical expertise, we also released step-by-step tutorials in the above GitHub repository that guide users through the process of transforming raw data into harmonized entities and relations, ensuring semantic consistency across heterogeneous datasets. Upon completing the tutorial procedures, the resulting BioMedGraphica database functions as a comprehensive knowledge base that can directly interface with the BioMedGraphica software for knowledge graph construction and analysis. In parallel, a dedicated software tutorial is also provided, demonstrating how to initiate the platform, configure workflows, and generate tailored TNG subsets. Together, these resources ensure that BioMedGraphica is not only transparent and reproducible but also accessible and scalable, thereby empowering researchers across biomedical, computational, and translational domains to leverage the system effectively.

### 4.2 BioMedGraphica knowledge graph data access

#### 4.2.1 Data preparation: entities and labels

Prior to using the platform, users are required to prepare and standardize input files, consisting of feature files and a sample label file. For proper integration, all sample IDs across files should be harmonized to the same identifier system and listed in the first column of each file, thereby avoiding mismatches during downstream processing. Feature columns are expected to use either standardized database identifiers (e.g. Ensembl stable Gene IDs, HGNC symbols) or well-defined textual names (e.g. drug names, HPO terms), with any abbreviations expanded to facilitate reliable soft matching (see GitHub repository for detailed formatting guidelines). During upload through the interface shown in Fig. 4A and Fig. 12A and C, available as supplementary data at *Bioinformatics* online, the platform performs real-time analysis of entity connectivity to ensure that the constructed knowledge graph remains fully connected. In cases where connectivity gaps are detected, the system automatically identifies missing components (see Fig. 4B and Fig. 12B, available as supplementary data at *Bioinformatics* online) and reminds users to supplement them as virtual entities, based on connectivity checks against the core signaling graph, $E^{\left(\mathrm{core}\right)}$, described in Section 3.2.2.

#### 4.2.2 Configuration finalization and data integration

Once all entities have been specified, the workflow proceeds to finalization, during which the system automatically determines an entity ordering that reflects the canonical progression of biological signaling processes (see Fig. 12D, available as supplementary data at *Bioinformatics* online). This ordering, derived from the feature names defined in the previous step, offers an intuitive representation of the underlying signaling hierarchy and facilitates biologically coherent graph construction. To accommodate specific experimental contexts or analytical preferences, users are given the option to manually refine this ordering through an interactive reordering panel. At this stage, additional configuration options are available, including the ability to enable *z*-score normalization of feature values and to specify which relation or edge types should be retained for downstream graph construction, thereby offering flexibility in tailoring the knowledge graph to the intended application.

After configuration is finalized, the system executes the data integration pipeline. This involves aligning common sample identifiers across all input files to ensure consistency, performing hard identifier matching for standardized nomenclature, and applying embedding-based soft matching for entities expressed in natural-language terms. The soft matching process is implemented with user-in-the-loop confirmation, balancing automation with human oversight to improve reliability. The result of this pipeline is a set of AI-ready outputs, including graph-structured feature matrices and entity-to-identifier mapping files. These outputs are systematically packaged and made available for download through the platform interface, enabling immediate use in graph-based AI models and downstream biomedical analyses.

### 4.3 Case study: generating TNG using BioMedGraphica

To demonstrate the practical utility and technical robustness of BioMedGraphica, we present a case study using a real multiomic dataset of Alzheimer’s disease (AD). Approximately 6.5 million people are living with AD in USA, and the estimated health-care cost is about $321 billion, which will increase to $1 trillion by 2050 (Alzheimer's Association 2022). There is no effective treatment for AD (Alzheimer's Association 2018, Cummings *et al.* 2018), which is partially due to the unknown signaling pathways (Mizuno *et al.* 2012, Godoy *et al.* 2014, Ofengeim *et al.* 2017, Verheijen and Sleegers 2018, Xu et al. 2018, Lazic *et al.* 2019, Li *et al.* 2022a, 2022b) that lead to neurodegeneration, though more than 50 genes/loci have been associated with AD (Sims *et al.* 2017, Verheijen and Sleegers 2018, Kunkle *et al.* 2019). TNGs can help address this gap by integrating multiomic features with prior biological knowledge to facilitate the discovery of disease-relevant molecular interactions and pathways. Specifically, in this case study, we used the Emory_Vascular dataset (Synapse ID: syn18909507) from the M2OVE-AD program, which contains transcriptomic measurements together with clinically derived variables and diagnostic information from a real-world prodromal Alzheimer’s disease cohort, making it well suited for demonstrating how BioMedGraphica integrates these data into a unified TNG representation. This representation is intended to support downstream predictive modeling tasks, such as distinguishing mild cognitive impairment/prodromal AD subjects from normal controls. To construct this case study in BioMedGraphica, users first preprocess the feature matrices such that the first column contains sample identifiers and the remaining columns contain feature values. The sample label file similarly uses the first column for sample IDs and the second column for class labels, with categorical labels encoded numerically if needed. All input files are provided in CSV, TXT, or TSV format, and a consistent sample ID scheme is required across all files. To enable automatic recognition of entity types, it is recommended that filenames of processed feature files include the corresponding entity type keywords (e.g. promoter, transcript, protein). No specific naming is required for the sample label file. After uploading the entity files, the system automatically assigns entity types and labels, which users may adjust if necessary. Users then specify the identifier system (e.g. Ensembl gene ID, HGNC symbol) or textual names (e.g. drug names, HPO terms) associated with each file. The overall workflow from input data processing to graph-ready output in this case study is illustrated in Fig. 11A, available as supplementary data at *Bioinformatics* online, while the corresponding BioMedGraphica web interface and user operations are shown in Fig. 4 and Fig. 12, available as supplementary data at *Bioinformatics* online.

During Step 1, users upload the required input files through the interface illustrated in Fig. 4A. A more detailed description of the Step 1 interface, including the functions of its individual fields and controls, is provided in Fig. 12A and C, available as supplementary data at *Bioinformatics* online. At this stage, the system evaluates knowledge graph connectivity using the shortest-path-based procedure described in Section 3.2.2, highlights any missing entity types, and allows supplementation either automatically through the *Add Missing Node* function or manually through user-defined virtual nodes. The connectivity status is updated in real time.

In this case study, the real input entity types were Transcript, Exposure, and Phenotype. Based on the abstract knowledge graph, BioMedGraphica first assessed whether these selected entity types formed a connected input-specific subgraph. Because Transcript belongs to the core biological chain, the system automatically introduced Protein as a virtual node to complete the minimal core segment required for connectivity. Subsequently, the connection between Protein and Exposure admitted two equally short abstract candidates, namely Protein–Pathway–Exposure and Protein–Disease–Exposure. Among these two options, Pathway was selected as the supplementary node because it provides a mechanism-oriented intermediate layer linking molecular alterations to external factors, whereas Disease is more appropriately treated as a downstream outcome concept. This choice maintains the constructed graph at a mechanistic level rather than introducing disease status as an intermediate connector. The corresponding missing node scenario for this case study is illustrated in Fig. 12B, available as supplementary data at *Bioinformatics* online.

Accordingly, after BioMedGraphica processing, the input-specific graph expanded from the original real input types (Transcript, Exposure, and Phenotype) to a connected schema that additionally includes the supplemented virtual entity types of Protein and Pathway. This supplementation also enabled biologically interpretable cross type connections in the generated TNG. For example, the transcript node BMGC_TS002362 (ENSG00000130203, APOE) was connected to the exposure node BMGC_EP0041 (Tobacco) through the virtual protein node BMGC_PT013443 (Apolipoprotein E) and the virtual pathway node BMGC_PW0011 (AD and miRNA effects pathway). This example illustrates how BioMedGraphica links molecular features and environmental exposures through intermediate biological entities and pathways, thereby recovering a biologically interpretable association that has been examined in the AD literature (Rusanen *et al.* 2010, Durazzo *et al.* 2014). A representative example of such biologically meaningful connectivity is shown in Fig. 11B, available as supplementary data at *Bioinformatics* online. Once the label file has been uploaded and validated, users may proceed to Step 2.

In Step 2, BioMedGraphica automatically determines the entity file order and selects the relevant edge types based on the biological hierarchy and the predefined relation schema. Users may review and optionally adjust these settings, including whether *z*-score normalization should be applied to the feature matrices. The configuration interface for Step 2 is shown in Fig. 12D, available as supplementary data at *Bioinformatics* online. After final confirmation, the job is submitted to the server, and the system generates a compressed archive containing graph-ready feature matrices and entity-to-ID mapping files. This step completes the data integration process.

## 5 Summary and conclusion

Omic data analysis plays a crucial role in precision medicine for identifying novel disease-related targets and pathways. However, translating numeric and statistical omic analysis results into new scientific discoveries remains a major challenge, as human experts must manually review predicted targets, assess their statistical significance, and search extensive, interconnected prior knowledge to generate hypotheses—a process that is subjective and not scalable. Large laboratories with rich resources can more easily test and validate discoveries, but smaller laboratories often struggle to generate the testable hypotheses due to limited infrastructure and knowledge networks. This creates barriers to equitable discovery and slows the broader impact of omic data. Recently, LLMs have begun to transform scientific discovery through their ability to interpret and reason with human-readable biomedical knowledge at scale. By integrating LLMs with multiomic data analysis, it becomes possible to automate hypothesis generation, improve scalability, and accelerate precision medicine research.

Our unique contributions of this study are three-fold. First, we introduce a novel data format, TNG, integrating biomedical prior knowledge with quantitative data. In this format, entity names, descriptions, and biological functions are represented as textual information, while multiomic profiles and other biomedical measurements are encoded as numeric values. To the best of our knowledge, this is the first systematic framework to propose TNG as a representation for integrating textual biomedical knowledge with biomedical data. Second, to facilitate the construction of TNGs, we developed *BioMedGraphica*, an all-in-one platform that integrates 11 entity types and 30 relation types from 43 biomedical databases into a harmonized knowledge graph containing over 2.3 million entities and 27 million relations. Beyond its scope and harmonization, BioMedGraphica also provides a GUI that enables researchers to generate customized TNG datasets, bridging fragmented biomedical data with curated prior knowledge through an accessible and reproducible workflow. Third, the resulting TNGs are directly applicable to graph foundation models and LLM augmentation, providing both predictive power and mechanistic interpretability. Together, these advances establish BioMedGraphica as not only one of the most comprehensive biomedical knowledge graph resources to date but also as a cornerstone for the development of next-generation graph foundation models in biomedicine, paving the way for scalable, interpretable, and evidence-based discoveries that empower both large and small laboratories in precision medicine.

## Supplementary Material

btag355_Supplementary_Data

## Data Availability

The data underlying this article are available in the BioMedGraphica Hugging Face dataset repository at https://huggingface.co/datasets/FuhaiLiAiLab/BioMedGraphica. The source code, processing scripts, and tutorials are available at https://github.com/FuhaiLiAiLab/BioMedGraphica. The integrated BioMedGraphica knowledge graph was derived from publicly available biomedical resources listed in the article and Supplementary Materials. The original third-party datasets can be accessed from their respective source databases and are subject to the terms and conditions of those databases.
