# Supplementary material for: BioMedGraphica: an all-in-one platform for joint textual biomedical prior knowledge and numeric graph generation
Source: Bioinformatics. 2026 Jun 5;42(6):btag355. doi: 10.1093/bioinformatics/btag355 (PMC13281933; doi:10.1093/bioinformatics/btag355)
Supplement: btag355_Supplementary_Data [file btag355_supplementary_data.docx]

**Supplementary Materials**

**Section A. Details of Data Resources**

**A.1. Data Resources for Entities**

**Ensembl**(Howe et al. 2021) Ensembl is a widely used resource for genome annotation and provides access to a wide variety of genomic data across numerous species, with a strong focus on vertebrate genomes. The Ensembl project integrates gene, transcript, and protein data, offering detailed genomic features. This database was accessed through the BioMart API, which allows flexible retrieval of large datasets based on specific criteria. For gene entities, Gene Stable IDs were selected, including versioned identifiers that ensure traceability across different releases. Important genomic features such as gene start and end positions, biotypes, and chromosomal coordinates were extracted. To maintain consistency in gene nomenclature, the mapping relationships between Ensembl and the HUGO Gene Nomenclature Committee (HGNC) were preserved. This consistency is crucial for ensuring that gene annotations align across various databases. Additionally, transcript entities were obtained using Transcript Stable IDs and corresponding Gene IDs, while protein entities were extracted with Protein Stable IDs. Mapping relationships between Ensembl, UniProt, and RefSeq were also preserved to ensure accurate and cross-compatible dataset integration.

**OMIM**(Amberger et al. 2009) **(Online Mendelian Inheritance in Man)** OMIM is an essential resource for understanding the genetic basis of human diseases and provides detailed information on gene-phenotype relationships. The database integrates clinical features with genetic data, offering insights into the hereditary nature of various conditions. Data from OMIM was retrieved to capture gene-related records, particularly focusing on mapping relationships between OMIM IDs and NCBI gene IDs. This facilitated the standardization of gene-related data across different resources. Furthermore, HGNC symbols were retained to align OMIM gene identifiers with other databases used in this study. Chromosomal information was also supplemented, which aids in genomic localization and contextual understanding of the data. Ensuring the uniqueness of gene records was a priority, and merging was performed based on gene IDs to guarantee that each entry in the dataset remained unique and free from redundancy.

**HGNC**(Povey et al. 2001) **(HUGO Gene Nomenclature Committee)** The HGNC is the authoritative resource for assigning unique symbols and names to human genes. As gene nomenclature can vary across different databases, the HGNC serves as a standard for the human genome, providing approved gene symbols and names. Data was accessed via BioMart to extract HGNC-approved gene information, including attributes such as HGNC ID, gene symbol, gene name, and chromosomal location. The inclusion of HGNC data ensures that gene-related information in the dataset is standardized and consistent with official naming conventions. Mapping relationships between HGNC, Ensembl, and NCBI IDs were retained to facilitate cross-referencing across these major databases. To ensure accuracy and prevent redundancy, the uniqueness of Ensembl IDs was verified during the merging process.

**NCBI**(Schoch et al. 2020) **(National Center for Biotechnology Information) - Gene** The NCBI Gene database provides extensive information on genes and their functions, supporting a wide range of research in genetics, genomics, and bioinformatics. For this study, human gene data was extracted, retaining key attributes such as NCBI gene IDs, gene symbols, descriptive gene names, and chromosomal positions. The NCBI Gene database is an important resource for identifying gene sequences, gene structure, and gene functions, making it essential for the construction of a comprehensive gene dataset. Mapping relationships between NCBI gene IDs and Ensembl IDs were preserved to ensure consistency across datasets, facilitating the integration of data from different sources. For microbiome-related data, entries from the NCBI Taxonomy Database were also included. This database provides authoritative taxonomic classifications, focusing on bacterial taxa, and corresponding NCBI Taxon IDs were retained to ensure accurate classification and integration with other microbiome datasets.

**NCBI - RefSeq**(O’Leary et al. 2016) **(Reference Sequence Database)** RefSeq is a curated collection of publicly available nucleotide sequences and their corresponding protein translations, which provides a critical reference standard for the annotation of genes, transcripts, and proteins. RefSeq data was retrieved for both gene and transcript entities in this study, focusing on entries with the status of either "REVIEWED" or "MODEL" to ensure high data quality. Essential attributes such as gene ID, RefSeq ID, and chromosomal information were retained to provide accurate gene annotations. Additionally, the MANE project, which provides a set of transcript alignments between RefSeq and Ensembl, was utilized to ensure that transcript mapping between these databases was consistent and high-quality. Protein entities were also integrated, with mapping relationships between RefSeq, UniProt, and Ensembl retained to ensure cross-database compatibility. Uniqueness of the Ensembl IDs was verified throughout the data processing stages to ensure data integrity.

**RNAcentral**(Sweeney et al. 2019) RNAcentral is a comprehensive resource for non-coding RNA sequences, integrating data from over 40 specialist databases. RNAcentral provides access to a wide variety of RNA sequence information, including microRNAs, tRNAs, and other functional RNA molecules that play critical roles in gene regulation. Human-specific RNAcentral IDs and corresponding Ensembl IDs were retrieved for this study, ensuring that non-coding RNA entities could be accurately integrated with gene and protein data from other databases. The uniqueness of each Ensembl ID was verified to ensure the integrity of the dataset and to avoid duplications during the integration process.

**UniProt**(Wu et al. 2006) **(Universal Protein Resource)** UniProt is a globally recognized repository of protein sequences and functional information. It provides detailed annotations on protein sequences, structure, function, and interactions. Data from UniProt was accessed via its API, and UniProt IDs, along with protein names and their corresponding Ensembl IDs, were retrieved. This enabled the integration of protein-specific data with the broader dataset, ensuring that protein information was accurately cross-referenced with gene and transcript data from Ensembl. The uniqueness of each Ensembl ID was verified during the data integration process to ensure consistency and to prevent errors in protein-related data.

**Reactome**(Fabregat et al. 2018) Reactome is a curated knowledgebase of biological pathways, and it is a key resource for understanding the molecular mechanisms underlying cellular processes. Human-specific pathway data was extracted from Reactome for this study, enabling the integration of pathway-related information with gene and protein data. The inclusion of Reactome pathways facilitates research into functional genomics and systems biology, where pathway analysis is critical for understanding complex biological processes.

**KEGG**(Kanehisa and Goto 2000) **(Kyoto Encyclopedia of Genes and Genomes)** KEGG is a comprehensive resource for understanding high-level functions and utilities of biological systems, such as cells, organisms, and ecosystems. Human pathway data was retrieved using the bioservices package, with a focus on integrating KEGG pathways with other biological pathways from Reactome and WikiPathways. The inclusion of KEGG enables the dataset to support metabolic and signaling pathway analysis, providing valuable insights into cellular functions and disease mechanisms.

**WikiPathways**(Kelder et al. 2012) WikiPathways is an open, collaborative platform for the curation of biological pathways. Data from WikiPathways was converted to CSV format, and human-specific pathways were filtered for inclusion in this study. Mapping relationships between WikiPathways, KEGG, and Reactome were maintained to ensure consistent integration of pathway-related data. The inclusion of WikiPathways supports research into a wide variety of biological pathways, complementing the curated data from Reactome and KEGG.

**Pathway Ontology**(Petri et al. 2014) Pathway Ontology provides a standardized framework for the classification of biological pathways and their relationships. Preprocessing of the OBO-formatted file enabled the extraction of PO IDs, and mapping relationships with KEGG and Reactome were preserved. This integration allows for comprehensive pathway analysis, ensuring that biological pathways from multiple sources can be consistently linked.

**ComPath**(Domingo-Fernández et al. 2018) ComPath is a database that integrates pathway mapping relationships across KEGG, Reactome, and WikiPathways. All equivalent mappings were selected for this study, ensuring that pathway data from different sources could be cross-referenced. This comprehensive approach to pathway integration enables in-depth biological pathway analysis and facilitates the exploration of molecular mechanisms underlying diseases.

**HMDB**(Wishart et al. 2022) **(Human Metabolome Database)** HMDB is the most comprehensive, freely accessible database of small molecule metabolites found in the human body. It provides extensive mapping relationships related to metabolomics data. For this study, HMDB data was parsed from XML files, retaining key attributes such as CAS number, SMILES, InChI, and mapping relationships with other databases. The inclusion of HMDB supports research into human metabolism, drug interactions, and disease mechanisms, enabling detailed metabolomics analysis.

**ChEBI**(Degtyarenko et al. 2008) **(Chemical Entities of Biological Interest)** ChEBI is a database focused on 'small' chemical compounds and is used extensively for research in chemistry and biology. ChEBI provides manually annotated information about the structure, formula, and biological roles of chemical entities. In this study, ChEBI data was selected for drug entities, particularly those with a 3-star rating to ensure the highest data quality. Important attributes such as ChEBI ID, InChI, and the mapping relationship to CAS Registry Numbers were retained. In addition to drug entities, metabolome data from ChEBI was included, focusing on human metabolites. Mapping relationships with other databases, such as the Human Metabolome Database (HMDB), were preserved to enable cross-referencing of metabolite information.

**SILVA**(Quast et al. 2012) SILVA is a high-quality, curated database of ribosomal RNA (rRNA) sequences, widely used for taxonomic classification of microbial communities. Data from both the small subunit (SSU) and large subunit (LSU) ribosomal RNA sequences were included, along with corresponding NCBI Taxon IDs. The SILVA database provides valuable insights into the composition of microbiomes, supporting research into microbial diversity and ecology.

**Greengenes**(DeSantis et al. 2006) Greengenes is a database of 16S ribosomal RNA gene sequences used for the identification of microbial species. Data was sourced from RNAcentral, and RNAcentral IDs, Greengenes IDs, and NCBI Taxon IDs were retained to ensure consistent taxonomic classification of microbiome-related data. The inclusion of Greengenes allows for the accurate classification of bacterial species, supporting research into microbiomes and their impact on human health.

**RDP**(Cole et al. 2014) **(Ribosomal Database Project)** The Ribosomal Database Project (RDP) provides quality-controlled ribosomal RNA gene sequence data. Similar to Greengenes, RDP data was sourced via RNAcentral, and mapping relationships between RNAcentral IDs, RDP IDs, and NCBI Taxon IDs were preserved. This allows for the consistent classification of microbial entities, supporting microbiome research and analysis.

**GTDB**(Parks et al. 2018) **(Genome Taxonomy Database)** GTDB is a comprehensive resource for the classification of Archaea and Bacteria. Data from GTDB was retrieved for both archaeal and bacterial entities, with GTDB IDs and NCBI Taxon IDs retained to ensure accurate taxonomic classification. By verifying the uniqueness of NCBI Taxon IDs, the dataset provides reliable support for microbiome research, enabling the exploration of microbial diversity across various environments.

**CTD**(Davis et al. 2021) **(The Comparative Toxicogenomics Database)** CTD is a pivotal resource for integrating chemical, gene, disease, and exposure data, facilitating the study of toxicogenomics and environmental health. CTD serves as an entity-centric database where chemicals, genes, and diseases are interconnected through curated interaction data. For chemical entities, CTD uses standardized identifiers such as Chemical Abstracts Service (CAS) numbers to ensure consistent representation and integration with other chemical databases like PubChem.

**HPO**(Köhler et al. 2021) **(Human Phenotype Ontology)** The Human Phenotype Ontology (HPO) provides a standardized vocabulary for phenotypic abnormalities encountered in human disease. Data was imported from the HPO database, version 2024-8-13, and relevant phenotype labels were extracted. After filtering and cleaning unwanted descriptive expressions, mapping relationships between HPO IDs were retained, ensuring that phenotypic data could be integrated with other disease and genomic datasets. This integration facilitates research into genotype-phenotype correlations, a key area in genetic and clinical research.

**ICD**(Organization 2004, 2018) **(International Classification of Diseases)** The International Classification of Diseases (ICD), maintained by the World Health Organization, is the global standard for the coding and classification of diseases. Both ICD-10 and ICD-11 codes were included to ensure that the dataset could be used in various research and clinical contexts. Mapping relationships between ICD versions were retained, allowing for compatibility across different healthcare systems and facilitating research on disease epidemiology and outcomes.

**Disease Ontology**(Schriml et al. 2012) **(DO)** The Disease Ontology (DO) provides a standardized ontology for the classification of human diseases. DO includes cross-references to other medical ontologies, such as UMLS, MeSH, and ICD-10, which were retained in this study to ensure consistent disease classification across databases. The inclusion of DO enabled the dataset to capture detailed and structured information on diseases, supporting research in medical informatics and bioinformatics.

**MeSH**(Lipscomb 2000) **(Medical Subject Headings)** MeSH is a comprehensive controlled vocabulary for the purpose of indexing journal articles and books in the life sciences. It is widely used in medical and biomedical research for categorizing diseases, drugs, and other entities. In this study, MeSH terms were retrieved from the MeSH XML files, focusing on records under the Diseases category. Mapping relationships with UMLS, ICD-10, and other disease ontologies were preserved to ensure consistency in terminology across datasets. This facilitated the integration of disease data and enabled the dataset to support detailed disease-related analyses.

**UMLS**(Bodenreider 2004) **(Unified Medical Language System)** The Unified Medical Language System (UMLS), developed by the National Library of Medicine (NLM), integrates multiple biomedical terminologies into a single framework. The Disease or Syndrome category of UMLS was selected for this study, with an emphasis on "Preferred" terms defined in English. Mapping relationships between UMLS, MeSH, SNOMED-CT, and ICD-10 were maintained to ensure accurate classification and cross-referencing of disease entities. The inclusion of UMLS data ensures that disease-related data can be consistently linked across multiple terminological systems, facilitating research in clinical informatics and biomedical research.

**SNOMED-CT**(Donnelly 2006) **(Systematized Nomenclature of Medicine Clinical Terms)** SNOMED-CT is an international clinical terminology that is used to code the entire scope of human medical practice, including diseases, symptoms, diagnoses, and treatments. Data from the Snapshot version of SNOMED-CT was used to extract active entries from the "Disorder" category, preserving mapping relationships with ICD-10. This allowed for the integration of clinical disease information with other ontologies, enhancing the utility of the dataset for both clinical and research applications.

**Mondo**(Vasilevsky et al. 2020) The Mondo Disease Ontology integrates multiple disease ontologies and databases, offering comprehensive cross-references to UMLS, MeSH, and other classification systems. Data from Mondo was included in this study, with a focus on preserving mapping relationships between Mondo IDs, UMLS, and MeSH. This integration enabled the consistent classification of disease entities, ensuring that disease-related data from different sources could be accurately linked.

**PubChem**(Wang et al. 2009) PubChem is a large database of chemical molecules and their biological activities, maintained by the National Center for Biotechnology Information (NCBI). It is widely used for retrieving chemical information related to small molecules, including drugs, metabolites, and other compounds. For this study, data from the Drug and Medication Information and Pharmacology and Biochemistry categories within the PubChem compound catalog was extracted. Key chemical descriptors, such as InChI, SMILES, InChIKey, and IUPAC names, were selected to provide detailed chemical structure information. The PubChem CID (Compound Identifier) was used as a unique identifier to facilitate consistent cross-referencing of chemical compounds across datasets.

**NDC**(Tribble 2024) **(National Drug Code)** The National Drug Code (NDC) is a unique identifier for medications in the United States, maintained by the U.S. Food and Drug Administration (FDA). It is an essential resource for drug-related data integration. Data from the NDC was selected for inclusion, focusing on the NDC code and substance names, which correspond to the UNII (Unique Ingredient Identifier) code’s preferred term. By ensuring the uniqueness of each substance name, accurate integration with UNII data was facilitated, allowing for comprehensive drug-related analyses.

**UNII**(Weisgerber 1997) **(Unique Ingredient Identifier)** The Unique Ingredient Identifier (UNII) system, maintained by the FDA, assigns unique identifiers to chemical substances, including active ingredients in drugs. UNII data was sourced from both the PubChem website and the FDA, with mapping relationships between UNII codes, PubChem CIDs, and CAS numbers being preserved. Additionally, structural descriptors such as SMILES and InChIKeys were included, providing a detailed representation of the chemical substances. This ensures that UNII data can be integrated seamlessly with other chemical and pharmacological databases.

**DrugBank**(Knox et al. 2024) DrugBank is a unique bioinformatics and cheminformatics resource that combines detailed drug data with comprehensive drug-target information. Data from DrugBank was included in this study to retain mapping relationships between DrugBank IDs and other chemical identifiers, such as PubChem CID, SID (Substance ID), and CAS numbers. DrugBank's extensive annotation of drug targets and mechanisms of action made it a valuable resource for cross-referencing drugs with their molecular and clinical effects, enabling more in-depth pharmacological studies.

**A.2 Data Resources for Relations**

**Ensembl**(Howe et al. 2021) Ensembl is a comprehensive genome browser and database that provides a wealth of information on gene sequences, annotations, and relationships across multiple species. It supports the analysis of gene-transcript interactions by linking genes to their corresponding transcripts. Ensembl also provides transcript-protein interaction, providing detailed annotations of how transcripts give rise to protein products. The dataset is essential for understanding gene structure, function, and the consequences of gene expression.

**NCBI - RefSeq**(O’Leary et al. 2016) **(Reference Sequence Database)** RefSeq is a well-curated collection of gene, transcript, and protein sequences, offering high-quality data for gene-transcript and transcript-protein relationships. It provides standardized and curated sequences that ensure consistency in gene annotations. RefSeq is crucial for researchers needing reliable reference sequences for various biological analyses, particularly in understanding the relationships between transcripts and their encoded proteins.

**UniProt**(Wu et al. 2006) **(Universal Protein Resource)** UniProt is a leading repository of protein sequence and functional information. It plays a dual role by linking transcripts to their corresponding protein products. In addition to capturing transcript-protein interactions, UniProt also includes annotations of protein-disease relationships, making it essential for understanding how protein dysfunctions can lead to disease.

**BioGrid**(Oughtred et al. 2019) BioGrid is a key resource for protein-protein interaction data, curated from both high-throughput and small-scale experimental studies. This database is essential for exploring how proteins interact within cellular networks, facilitating the study of complex biological processes such as signaling pathways, metabolic networks, and structural assemblies. BioGrid data on protein-protein interactions supports a wide range of applications, from basic research to drug discovery.

**STRING**(Szklarczyk et al. 2019) STRING is a database of known and predicted protein-protein interactions, integrating data from various sources such as experimental studies, computational predictions, and publicly available text collections. It is essential for understanding the functional interactions between proteins and mapping protein interaction networks. STRING helps to identify potential interactions that play critical roles in biological processes and disease states, making it a valuable tool for systems biology research.

**KEGG**(Kanehisa and Goto 2000) **(Kyoto Encyclopedia of Genes and Genomes)** KEGG is a comprehensive database that integrates genomic, chemical, and systemic functional information, offering valuable insights into various biological interactions. It is essential for studying protein-protein interactions, illustrating how proteins cooperate in cellular processes, as well as gene-pathway interactions, showing how genes function within specific biological pathways. Furthermore, KEGG explores drug-pathway interactions, revealing how drugs influence these pathways, and facilitates the study of pathway-gene and pathway-drug interactions, providing a clear understanding of how pathways are regulated by genes and targeted by drugs.

**HPO**(Köhler et al. 2021) **(Human Phenotype Ontology)** HPO provides a standardized vocabulary of phenotypic abnormalities associated with human diseases. It is invaluable for connecting genes to phenotypes (gene-phenotype interaction), linking diseases to their phenotypic presentations (disease-phenotype interaction), and mapping genes to diseases (gene-disease interaction). HPO also facilitates the study of phenotype-phenotype relationships, enabling researchers to compare phenotypic similarities and differences across genetic conditions.

**DisGeNet**(Piñero et al. 2016) DisGeNet is a comprehensive platform that integrates data on gene-disease associations from multiple sources, including expert-curated databases, scientific literature, and publicly available repositories. It plays a critical role in identifying gene-disease interactions, helping to elucidate the genetic basis of various diseases. DisGeNet supports research into disease mechanisms by providing insights into the complex genetic networks that underlie disease phenotypes.

**DISEASES**(Pletscher-Frankild et al. 2015) The DISEASES database provides information on protein-disease associations, integrating data from literature mining and manually curated sources. It links proteins to the diseases they are associated with, offering a detailed view of how protein dysfunctions contribute to disease phenotypes. DISEASES is especially useful for identifying molecular mechanisms underlying diseases and for exploring potential therapeutic targets.

**HMDB**(Wishart et al. 2022) **(Human Metabolome Database)** HMDB is an extensive resource that provides detailed information on human metabolites, including drugs, drug metabolites, and endogenous small molecules. It captures a wide range of interactions, including drug-metabolome, metabolome-disease, and metabolome-protein relationships. HMDB supports research in metabolomics, systems biology, and pharmacology, providing data on metabolic pathways, metabolite-protein interactions, and the role of metabolites in health and disease.

**MetaNetX**(Moretti et al. 2021) It is a comprehensive resource developed by the SIB Swiss Institute of Bioinformatics to facilitate the standardization, integration, and analysis of genome-scale metabolic networks (GSMNs) and biochemical pathways. MetaNetX allows users to construct, modify, and analyze metabolic models through tools for flux balance analysis (FBA), reaction knockout simulations, and network comparison. By integrating data from diverse sources and providing a standardized framework, MetaNetX is a valuable tool for researchers in systems biology and bioinformatics, enabling a deeper understanding of complex metabolic processes.

**DisBiome**(Janssens et al. 2018) DisBiome is a database that focuses on the relationships between microbiomes and diseases. It captures microbiome-disease interactions, providing insights into how microbial taxa are associated with health and disease. DisBiome supports research into the role of the human microbiome in various disease conditions, facilitating the exploration of microbial communities as potential biomarkers or therapeutic targets.

**MDAD**(Sun et al. 2018) **(Microbe-Drug Association Database)** MDAD is a comprehensive resource that compiles clinically and experimentally validated associations between microbes and drugs. It contains 5,055 entries, encompassing 1,388 drugs and 180 microbes, sourced from multiple drug databases and scientific publications. Each record in MDAD includes detailed annotations, such as molecular forms of drugs, links to DrugBank, microbe target information from UniProt, and original reference citations. This database serves as a valuable tool for researchers aiming to understand microbe-drug interactions, facilitating advancements in drug discovery, disease therapy, and personalized medicine.

**PharmacoMicrobiomics**(Doestzada et al. 2018) It is a field that examines the interactions between the human microbiome and drugs, focusing on how microbial communities influence drug metabolism, efficacy, and toxicity. This bidirectional relationship involves microbes activating, inactivating, or transforming drugs into metabolites with altered effects, while drugs, in turn, can reshape the composition and function of the microbiome. These interactions have profound implications for personalized medicine, as variations in the microbiome can affect individual drug responses, side effects, and therapeutic outcomes. By understanding these dynamics, PharmacoMicrobiomics aims to optimize drug therapies, reduce adverse effects, and pave the way for microbiome-targeted medical interventions.

**CTD (The Comparative Toxicogenomics Database)**(Davis et al. 2021) CTD is a publicly available, manually curated resource that provides insights into the complex relationships between chemicals, genes, and diseases, with a specific emphasis on environmental exposures. CTD integrates data on chemical-gene interactions, chemical-disease associations, and gene-disease relationships, offering researchers a unique platform to explore the molecular mechanisms underlying toxicological effects and exposure-related health outcomes. By including exposure-related information, CTD helps bridge the gap between environmental science and molecular biology, enabling studies on how environmental factors influence gene function and contribute to disease etiology. This resource is particularly valuable for advancing research in toxicogenomics, precision medicine, and environmental health.

**DO (Disease Ontology)**(Schriml et al. 2012) DO is a standardized biomedical ontology that provides a structured vocabulary and hierarchical classification for human diseases, enabling consistent annotation and integration of disease-related data across research and clinical domains. Each disease entry is assigned a unique identifier and is cross-referenced with external resources such as OMIM, ICD, SNOMED CT, and MeSH, ensuring interoperability and facilitating data harmonization. By linking diseases to their etiology, molecular mechanisms, and clinical manifestations, DO supports applications in translational medicine, computational biology, and precision medicine. Its integration with genomic and phenotypic datasets makes it a critical tool for advancing disease research, biomarker discovery, and therapeutic development.

**DrugBank**(Knox et al. 2024) DrugBank is a comprehensive resource that integrates detailed information on drugs and their targets. It captures multiple types of interactions, including protein-drug, drug-drug relationships. DrugBank provides data on drug mechanisms, drug interactions, and the diseases they are used to treat, making it an essential tool for pharmacological research and drug development. It also supports studies on how drugs interact with biological systems at the molecular level.

**BindingDB**(Gilson et al. 2016) BindingDB is a public repository of measured binding affinities between proteins (mainly drug targets) and small, drug-like molecules. It supports research into protein-drug interactions by providing experimental data on the binding affinities of drugs to their target proteins. BindingDB is a valuable resource for drug discovery and pharmacology, helping researchers identify potential drug candidates and understand the molecular mechanisms of drug action.

**DrugCentral**(Ursu et al. 2016) DrugCentral is a centralized portal for drug information, offering data on drug-protein, drug-disease, interactions. It integrates information on drug indications, targets, and mechanisms of action, supporting the study of therapeutic interventions and pharmacodynamics. DrugCentral is an important resource for researchers exploring drug repurposing, drug development, and clinical applications.

**SIDER**(Kuhn et al. 2010) **(Side Effect Resource)** SIDER provides comprehensive data on the adverse effects of drugs, linking pharmaceutical compounds to their phenotypic side effects. This resource is essential for studying drug-phenotype interactions, helping researchers understand the unintended consequences of drug use. SIDER supports pharmacovigilance efforts and aids in optimizing drug safety profiles by highlighting potential risks associated with pharmaceutical compounds.

**Table S1**. Download Links and Access Control for Entity Databases

| **Database** | | **Access** | **Download Link** |
| --- | --- | --- | --- |
| Ensembl | Gene | public access | BioMart API |
|  | Transcript | public access | BioMart API |
|  | Protein | public access | BioMart API |
| OMIM | Gene | public access | <https://omim.org/static/omim/data/mim2gene.txt> |
| HGNC | Gene | public access | <https://www.genenames.org/cgi-bin/download/custom?col=gd_hgnc_id&col=gd_app_sym&col=gd_app_name&col=gd_pub_eg_id&col=gd_pub_ensembl_id&status=Approved&hgnc_dbtag=on&order_by=gd_hgnc_id&format=text&submit=submit> |
| NCBI | Gene | public access | <https://ftp.ncbi.nlm.nih.gov/gene/DATA/gene2ensembl.gz>  <https://ftp.ncbi.nlm.nih.gov/gene/DATA/gene_info.gz> |
|  | Microbiota | public access | <https://ftp.ncbi.nih.gov/pub/taxonomy/taxdmp.zip> |
| RefSeq | Gene | public access | <https://ftp.ncbi.nlm.nih.gov/gene/DATA/gene2refseq.gz> |
|  | Transcript | public access | <https://ftp.ncbi.nlm.nih.gov/refseq/MANE/MANE_human/current/MANE.GRCh38.v1.3.summary.txt.gz> |
|  | Protein | public access | <https://ftp.ncbi.nlm.nih.gov/gene/DATA/gene_refseq_uniprotkb_collab.gz>  <https://ftp.ncbi.nlm.nih.gov/gene/DATA/gene2ensembl.gz> |
| RNACentral | Transcript | public access | <https://ftp.ebi.ac.uk/pub/databases/RNAcentral/current_release/id_mapping/database_mappings/ensembl.tsv> |
| UniProt | Protein | public access | API |
| Reactome | Pathway | public access | <https://reactome.org/download/current/ReactomePathways.txt> |
| KEGG | Pathway | public access | Fetching data via R and Python |
| WikiPathways | Pathway | public access | Fetching data via Python |
| Pathway Ontology | Pathway | public access | <https://download.rgd.mcw.edu/ontology/pathway/pathway.obo> |
| ComPath | Pathway | public access | <https://compath.scai.fraunhofer.de/export_mappings> |
| HMDB | Metabolite | public access | <https://hmdb.ca/downloads> |
| ChEBI | Metabolite | public access | <https://www.ebi.ac.uk/chebi/chebiOntology.do?chebiId=77746> |
|  | Drug | public access | <https://ftp.ebi.ac.uk/pub/databases/chebi/Flat_file_tab_delimited/chebiId_inchi_3star.tsv> |
|  |  |  | <https://ftp.ebi.ac.uk/pub/databases/chebi/Flat_file_tab_delimited/database_accession_3star.tsv> |
| SILVA | Microbiota | public access | <https://www.arb-silva.de/fileadmin/silva_databases/current/Exports/taxonomy/ncbi/taxmap_embl-ebi_ena_lsu_ref_138.2.txt.gz> |
|  |  |  | <https://www.arb-silva.de/fileadmin/silva_databases/current/Exports/taxonomy/ncbi/taxmap_embl-ebi_ena_ssu_ref_138.2.txt.gz> |
| Greengenes | Microbiota | public access | <https://ftp.ebi.ac.uk/pub/databases/RNAcentral/current_release/id_mapping/database_mappings/greengenes.tsv> |
| RDP | Microbiota | public access | <https://ftp.ebi.ac.uk/pub/databases/RNAcentral/current_release/id_mapping/database_mappings/rdp.tsv> |
| GTDB | Microbiota | public access | <https://data.ace.uq.edu.au/public/gtdb/data/releases/latest/ar53_metadata.tsv.gz>  <https://data.ace.uq.edu.au/public/gtdb/data/releases/latest/bac120_metadata.tsv.gz> |
| CTD | Exposure | public access | <https://ctdbase.org/reports/CTD_chemicals.csv.gz> |
| HPO | Phenotype | public access | <https://hpo.jax.org/data/ontology> |
| UMLS | Phenotype | Registration required | <https://download.nlm.nih.gov/umls/kss/2024AA/umls-2024AA-full.zip?_gl=1*14ig82q*_ga*MTA5NTI1Nzc2My4xNzEwOTU5NjM5*_ga_7147EPK006*MTcyMzU3NDM0NC41My4xLjE3MjM1NzUyNzYuMC4wLjA.*_ga_P1FPTH9PL4*MTcyMzU3NDM0NC41My4xLjE3MjM1NzUyNzYuMC4wLjA> |
|  | Disease | Registration required |  |
| ICD10 / ICD11 | Disease | public access | <https://icdcdn.who.int/static/releasefiles/2024-01/SimpleTabulation-ICD-11-MMS-en.zip>  <https://icdcdn.who.int/static/releasefiles/2024-01/mapping.zip> |
| Disease Ontology | Disease | public access | <https://github.com/DiseaseOntology/HumanDiseaseOntology/blob/main/DOreports/allXREFinDO.tsv> |
| MeSH | Disease | public access | <https://nlmpubs.nlm.nih.gov/projects/mesh/MESH_FILES/xmlmesh/desc2024.xml> |
| SNOMED-CT | Disease | Registration required | <https://download.nlm.nih.gov/umls/kss/IHTSDO2024/IHTSDO20240801/SnomedCT_InternationalRF2_PRODUCTION_20240801T120000Z.zip?_gl=1*xret7k*_ga*MTA5NTI1Nzc2My4xNzEwOTU5NjM5*_ga_7147EPK006*MTcyMzU4ODA3OC41NC4xLjE3MjM1ODgyNDYuMC4wLjA.*_ga_P1FPTH9PL4*MTcyMzU4ODA3OS41NC4xLjE3MjM1ODgyNDYuMC4wLjA> |
| Mondo | Disease | public access | <https://github.com/monarch-initiative/mondo/blob/master/reports/xrefs.tsv>  <https://github.com/monarch-initiative/mondo/releases/latest/download/mondo.obo> |
| PubChem | Drug | public access | <https://pubchem.ncbi.nlm.nih.gov/#query=RgHgEbsv3pPpudygXtiVibNCzyJqQo7z9NaVv-Hh77v3rs&alias=PubChem%20Compound%20TOC:%20Drug%20and%20Medication%20Information> |
|  |  |  | <https://pubchem.ncbi.nlm.nih.gov/#query=fDvaK_Z8k8Ck7hv3mY9S3nQVH3UjzJXS7_eOnvTmnJ_0_6A&alias=PubChem%20Compound%20TOC:%20Pharmacology%20and%20Biochemistry> |
| NDC | Drug | public access | <https://www.accessdata.fda.gov/cder/ndctext.zip> |
| UNII | Drug | public access | <https://precision.fda.gov/uniisearch/archive/latest/UNII_Data.zip>  Fetching data via Python |
| DrugBank | Drug | public access | <https://go.drugbank.com/releases/5-1-12/downloads/all-drug-links> |

**Table S2**. Download Links and Access Control for Relation Databases

| **Database** | | **Access** | **Download Link** |
| --- | --- | --- | --- |
| Ensembl | Gene-Transcript | public access | BioMart API |
|  | Transcript-Protein |  | BioMart API |
| RefSeq | Gene-Transcript | public access | <https://ftp.ncbi.nlm.nih.gov/refseq/H_sapiens/RefSeqGene/LRG_RefSeqGene> |
|  | Transcript-Protein |  | <https://ftp.ncbi.nlm.nih.gov/refseq/H_sapiens/RefSeqGene/LRG_RefSeqGene> |
| UniProt | Transcript-Protein | public access | API |
|  | Protein-Disease |  | <https://rest.uniprot.org/uniprotkb/stream?compressed=true&fields=accession%2Ccc_disease&format=tsv&query=%28*%29+AND+%28model_organism%3A9606%29> |
| BioGrid | Protein-Protein | public access | <https://downloads.thebiogrid.org/File/BioGRID/Release-Archive/BIOGRID-4.4.237/BIOGRID-ALL-4.4.237.mitab.zip> |
| STRING | Protein-Protein | public access | <https://stringdb-downloads.org/download/protein.links.full.v12.0/9606.protein.links.full.v12.0.txt.gz> |
| KEGG | Protein-Protein | public access | Fetching data via R |
|  | Protein-Pathway |  |  |
|  | Drug-Pathway |  |  |
|  | Pathway- Protein |  |  |
|  | Pathway-Drug |  |  |
| HPO | Protein-Phenotype | public access | <https://hpo.jax.org/data/annotations> |
|  | Protein-Disease |  | <https://hpo.jax.org/data/annotations> |
|  | Phenotype-Phenotype |  | <https://hpo.jax.org/data/ontology> |
|  | Phenotype-Disease |  | <https://hpo.jax.org/data/annotations> |
|  | Disease-Phenotype |  | <https://hpo.jax.org/data/annotations> |
| DisGeNet | Protein-Disease | Registration required | API |
| DISEASES | Protein-Disease | Registration required | <https://download.jensenlab.org/human_disease_benchmark.tsv> |
| MetaNetX | Metabolite- Metabolite | public access | <https://www.metanetx.org/cgi-bin/mnxget/mnxref/chem_xref.tsv> |
|  |  |  | <https://www.metanetx.org/cgi-bin/mnxget/mnxref/chem_isom.tsv> |
| DisBiome | Microbiota-Disease | public access | <https://disbiome.ugent.be/export> |
| MDAD | Microbiota-Drug | public access | <https://github.com/Sun-Yazhou/MDAD/blob/master/MDAD.zip> |
|  | Drug-Microbiota |  |  |
| PharmacoMicrobiomics | Microbiota-Drug | public access | <http://pharmacomicrobiomics.com/view/relation/> |
|  | Drug-Microbiota |  |  |
| HMDB | Metabolite-Protein | public access | <https://hmdb.ca/downloads> |
|  | Metabolite-Disease |  |  |
|  | Drug-Metabolite |  |  |
| CTD | Exposure-Gene | public access | <https://ctdbase.org/reports/CTD_chem_gene_ixns.csv.gz> |
|  | Exposure-Pathway |  | <https://ctdbase.org/reports/CTD_chem_pathways_enriched.csv.gz> |
|  | Exposure-Disease |  | <https://ctdbase.org/reports/CTD_chemicals_diseases.csv.gz> |
| Disease Ontology | Disease-Disease | public access | <https://raw.githubusercontent.com/DiseaseOntology/HumanDiseaseOntology/refs/heads/main/src/ontology/HumanDO.obo> |
| DrugBank | Drug-Protein | Registration required | <https://go.drugbank.com/releases/5-1-12/downloads/target-all-polypeptide-ids> |
|  | Drug-Drug |  | <https://go.drugbank.com/releases/5-1-12/downloads/all-full-database> |
| BindingDB | Drug-Protein | public access | <https://www.bindingdb.org/bind/downloads/BindingDB_All_202409_tsv.zip> |
| DrugCentral | Drug-Protein | public access | <https://drugcentral.org/ActiveDownload> |
|  | Drug-Disease |  |  |
| SIDER | Drug-Phenotype | public access | <http://sideeffects.embl.de/media/download/meddra_all_se.tsv.gz> |

**Section B. Details of Entity and Relation Integration**

**B.1 Entity Integration**

**Gene Entity Merging** The Ensembl database was utilized as the primary basis for data integration. Initially, data from Ensembl, HGNC, and NCBI were merged based on matching Ensembl IDs. Subsequently, data from RefSeq and OMIM were incorporated, with NCBI IDs serving as the common identifier. The NCBI ID was chosen as the minimal unit for unifying the data, and a final integration of gene information was conducted according to NCBI IDs (refer to Table S3 and Figure S1 in the supplementary section for details). The columns highlighted in bold within the table denote those used for merging across databases, with the IDs in these columns being unique. Additionally, a textual description of each gene entity was appended. Using the free Perl script geneDocSum.pl provided by NCBI (download link: https://ftp.ncbi.nih.gov/gene/tools/geneDocSum.pl), all human records marked as current (alive) and containing summaries were retrieved. By mapping NCBI Gene IDs to corresponding entries in BioMedGraphica_Gene, the descriptions associated with BioMedGraphica_Gene IDs (BMG_GN) were obtained.

**Table S3**. Gene Entity Information

| Database Names | | Raw Data | | After Data Cleaning | | Total Number of BioMedGraphica ID / BioMedGraphica Connected ID |
| --- | --- | --- | --- | --- | --- | --- |
|  |  | Total Number of Rows | Unique | Total Number of Rows | Unique |  |
| Ensembl | **Ensembl Gene ID** | 86,406 | 86,402 | 86,402 | 86,402 | 230,358 / 86,238 |
|  | Ensembl Gene ID version | 86,406 | 86,402 | 86,402 | 86,402 |  |
|  | HGNC ID | 46,596 | 41,062 | 41,060 | 41,060* |  |
|  | Total Number of Rows | 86,406 | | 86,402 | |  |
| HGNC | HGNC ID | 43,916 | 43,916 | 43,915 | 43,915* |  |
|  | **Ensembl Gene ID** | 41,278 | 41,277 | 41,277 | 41,277 |  |
|  | NCBI Gene ID | 43,788 | 43,788 | 43,787 | 43,787* |  |
|  | Total Number of Rows | 43,916 | | 43,915 | |  |
| NCBI Gene | **NCBI Gene ID** | 38,465 | 38,434 | 38,189 | 38,189* |  |
|  | Ensembl Gene ID | 38,465 | 38,189 | 38,189 | 38,158 |  |
|  | Total Number of Rows | 38,465 | | 38,189 | |  |
| RefSeq | RefSeq ID | 845,230 | 151,250 | 160,846 | 135,156* |  |
|  | **NCBI Gene ID** | 845,230 | 191,104 | 160,846 | 160,846 |  |
|  | Total Number of Rows | 845,230 | | 160,846 | |  |
| OMIM | OMIM ID | 17,463 | 17,463 | 17,360 | 17,360* |  |
|  | **NCBI Gene ID** | 17,372 | 17,360 | 17,360 | 17,360 |  |
|  | Total Number of Rows | 17,463 | | 17,360 | |  |

*represent this column contains multiple IDs in one row


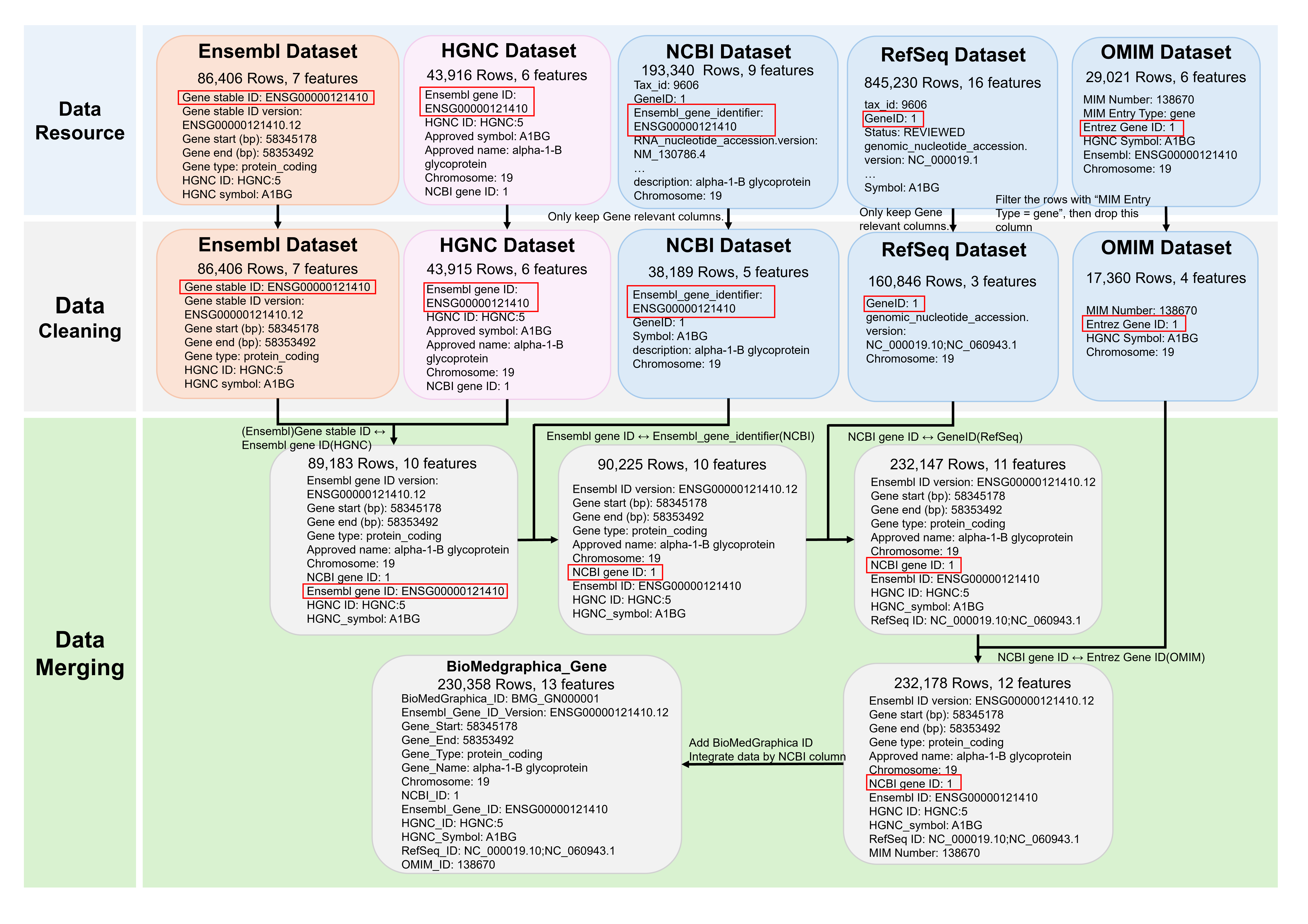


**Figure S1**. Details of Gene Entity Merging Process

**Figure S1** provides a detailed overview of the integration process for BioMedGraphica Gene, using A1BG as an example. The “Data Resource” section depicts the original datasets sourced from various databases for gene entity integration. The “Data Cleaning” section presents the cleaned data format prepared for integration. Columns highlighted in red boxes indicate the key matching fields used during the merging process. In the “Data Merging” section, the gray boxes showcase the data format at each step of database integration. The overall gene entity integration employs an outer join approach: Ensembl and HGNC databases are merged first, followed by integration with the NCBI Gene database. Subsequently, RefSeq and OMIM are incorporated sequentially. The final unification is based on the NCBI Gene ID, ensuring that all entries with the same ID are consolidated.

**Transcript Entity Merging** The integration of the three databases utilized the Ensembl ID as the standard reference. For transcript entities, the Ensembl Transcript Stable ID was adopted as the smallest unit of data granularity. The integration process is illustrated in **Figure S2** of the supplementary section, and the merged results are detailed in **Table S4**. Bolded entries in the table identify the columns used for database merging, where the IDs in these columns are unique. Transcript descriptions were extracted from the Ensembl database using the BioMart API. By mapping the Transcript Stable ID to corresponding transcripts in BioMedGraphica, transcript descriptions were successfully assigned to the majority of BioMedGraphica transcripts.

**Table S4**. Transcript Entity Information

| **Database** | | **Raw Data** | | **After Data Cleaning** | | Total Number of BioMedGraphica ID / BioMedGraphica Connected ID |
| --- | --- | --- | --- | --- | --- | --- |
|  |  | **Total Number of Rows** | **Unique** | **Total Number of Rows** | **Unique** |  |
| Ensembl | Ensembl Transcript ID | 451,959 | 412,034 | 451,959 | 412,034 | 412,326 / 412,039 |
|  | **Ensembl Transcript ID version** | 451,959 | 412,034 | 451,959 | 412,034 |  |
|  | Ensembl Gene ID | 451,959 | 86,402 | 451,959 | 86,402 |  |
|  | RefSeq mRNA ID | 85,642 | 66,939 | 85,642 | 66,939 |  |
|  | RefSeq ncRNA ID | 35,946 | 20,199 | 35,946 | 20,199 |  |
|  | RefSeq MANE Select ID | 38,501 | 19,287 | 38,501 | 19,287 |  |
|  | Total Number of Rows | 451,959 | | 451,959 | |  |
| RefSeq | **Ensembl Transcript ID version** | 19,404 | 19,404 | 19,404 | 19,404 |  |
|  | RefSeq ID | 19,404 | 19,404 | 19,404 | 19,404 |  |
|  | Total Number of Rows | 19,404 | | 19,404 | |  |
| RNAcentral | RNAcentral ID | 66,789 | 62,925 | 66,789 | 62,925 |  |
|  | **Ensembl Transcript ID** | 66,789 | 66,789 | 66,789 | 66,789 |  |
|  | Total Number of Rows | 66,789 | | 66,789 | |  |

*represent this column contains multiple IDs in one row


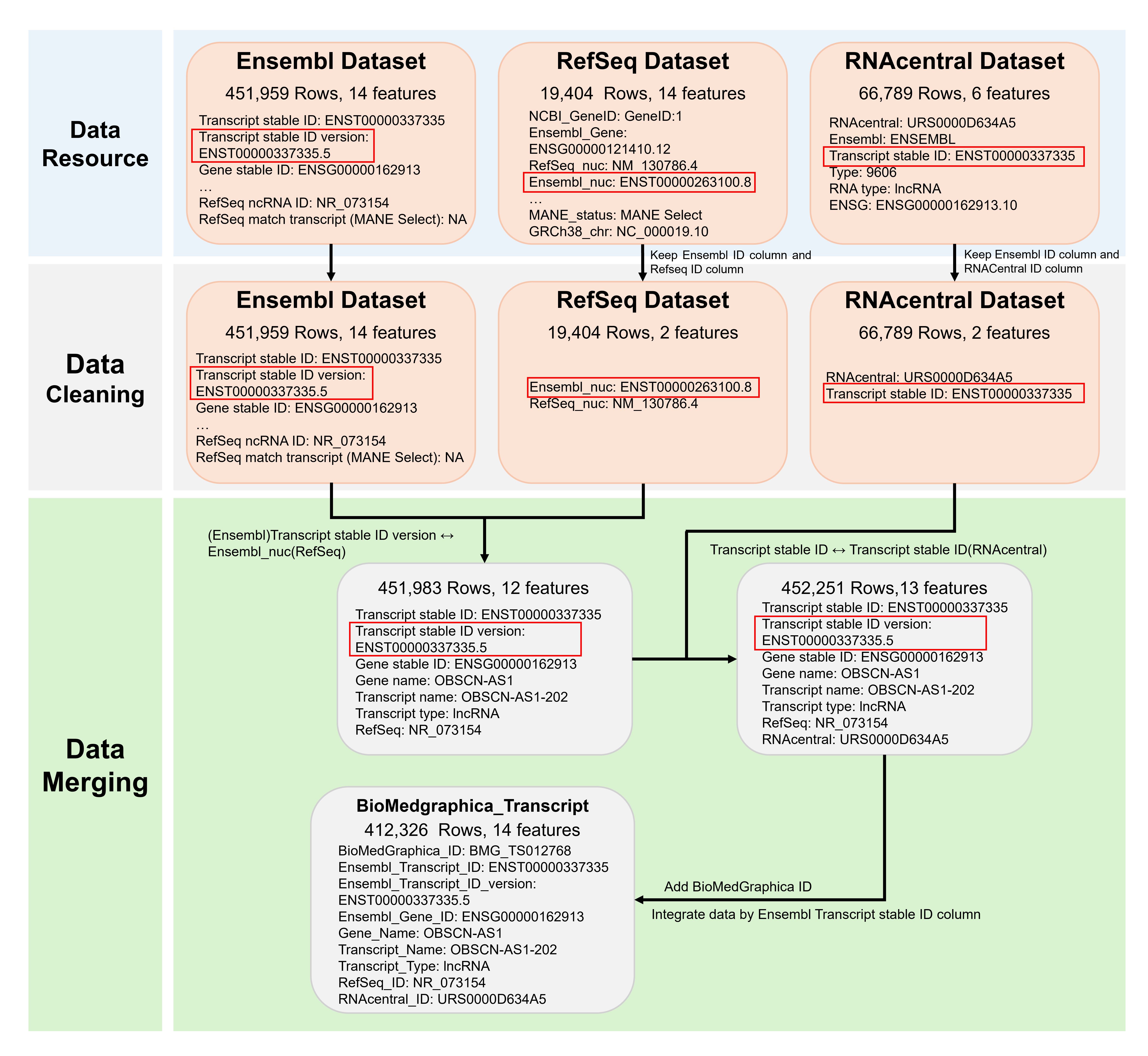


**Figure S2**. Details of Transcript Entity Merging Process

A detailed depiction of the integration process for BioMedGraphica transcript has been provided **in Figure S2**, using ENST00000337335.5 as an example. The "Data Resource" section shows the raw data sourced from databases used in transcript entity integration. Since the original RefSeq dataset lacked a corresponding RefSeq ID for ENST00000337335.5, an alternative transcript was selected as a supplementary example. The "Data Cleaning" section presents the cleaned data format prepared for integration. Columns highlighted in red boxes indicate the key matching fields used during the merging process. In the "Data Merging" section, gray boxes illustrate the data format after each step of database integration. The integration process for transcript entities employs an outer join approach: first, the Ensembl and RefSeq databases are merged, followed by integration with the RNAcentral database. The Ensembl stable ID serves as the primary unit for final data unification, consolidating all entries with the same Ensembl stable ID.

**Protein Entity Merging** The integration process began by merging data from Ensembl and UniProt based on the Protein Stable ID Version. Subsequently, RefSeq data was incorporated by leveraging mapping relationships between RefSeq and the two databases. The Ensembl Protein ID Version was established as the minimal unit of data granularity for protein entities (refer to **Figure S3** in the supplementary section for the merging workflow and **Table S5** for detailed results). Bolded entries in the table highlight the columns used for cross-database merging, where the IDs are uniquely assigned. Protein descriptions were retrieved from the UniProt database using the UniProt API. By mapping UniProt IDs to corresponding proteins in BioMedGraphica, descriptive information was successfully provided for BioMedGraphica proteins.

**Table S5**. Protein Entity Information

| Database | | | Raw Data | | After Data Cleaning | | Total Number of BioMedGraphica ID / BioMedGraphica Connected ID |
| --- | --- | --- | --- | --- | --- | --- | --- |
|  |  |  | Total Number of Rows | Unique | Total Number of Rows | Unique |  |
| Ensembl | | Ensembl Protein ID | 157,628 | 123,845 | 123,845 | 123,845 | 173,978 / 121,419 |
|  |  | **Ensembl Protein ID version** | 157,628 | 123,845 | 123,845 | 123,845 |  |
|  |  | UniProt ID | 77,235 | 19,350 | 50,516 | 19,345* |  |
|  |  | RefSeq ID | 80,200 | 66,793 | 47,583 | 42,935* |  |
|  |  | Total Number of Rows | 157,628 | | 123,845 | |  |
| UniProt | | UniProt ID | 20,417 | 20,417 | 51,853 | 20,417 |  |
|  |  | **Ensembl Protein ID version** | 19,329 | 19,329* | 50,765 | 50,765 |  |
|  |  | Total Number of Rows | 20,417 | | 51,853 | |  |
| RefSeq | RefSeq-Uniprot | RefSeq ID | 376,561 | 142,506 | 99,776 | 82,031* |  |
|  |  | **UniProt ID** | 376,561 | 117,805 | 99,776 | 99,776 |  |
|  |  | Total Number of Rows | 376,561 | | 99,776 | |  |
|  | RefSeq-Ensembl | RefSeq ID | 47,223 | 47,183 | 47,223 | 47,183 |  |
|  |  | **Ensembl Protein ID version** | 47,223 | 47,223 | 47,223 | 47,223 |  |
|  |  | Total Number of Rows | 68,932 | | 47,223 | |  |

*represent this column contains multiple IDs in one row


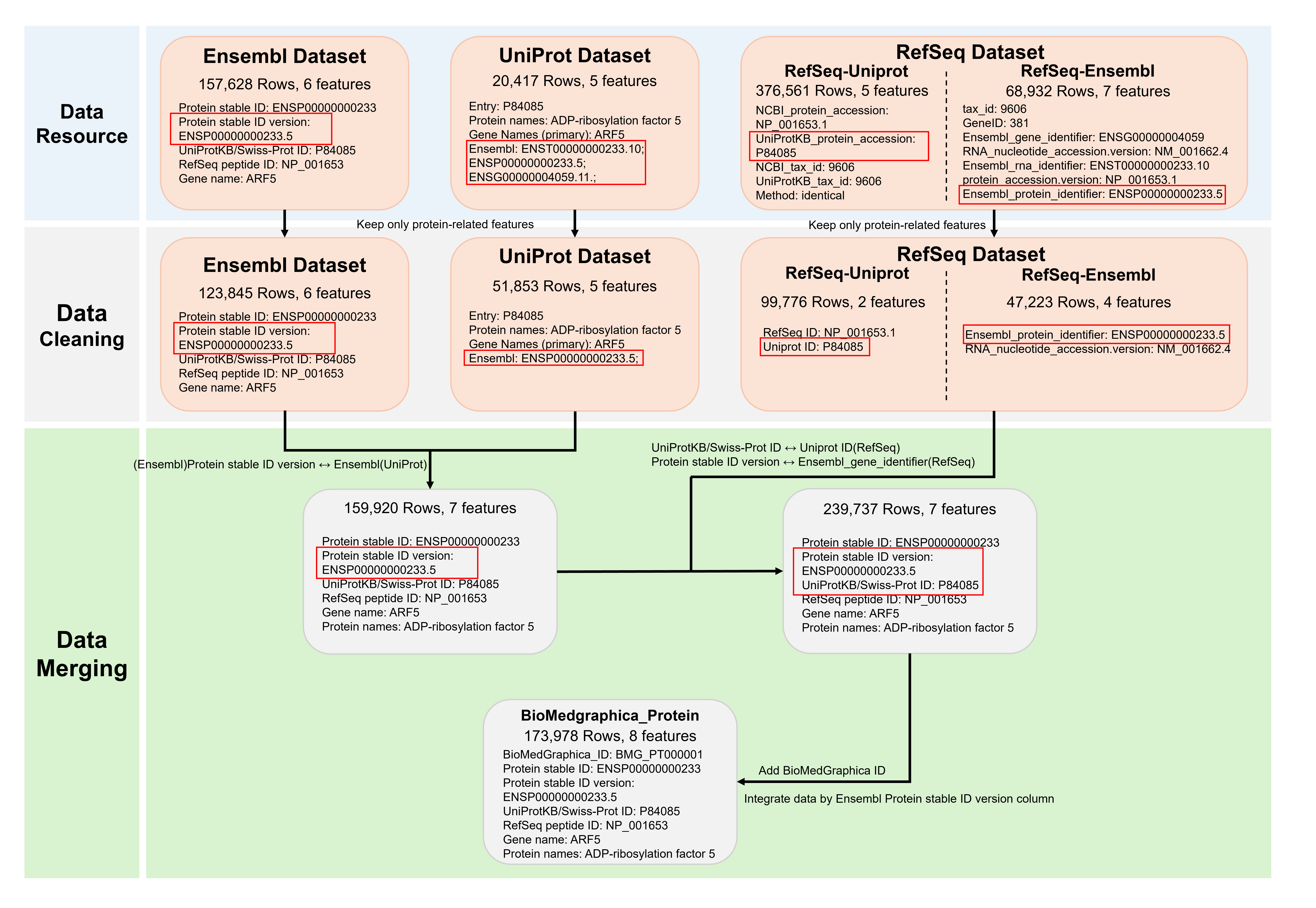


**Figure S3**. Details of Protein Entity Merging Process

**Figure S3** illustrates the integration process for BioMedGraphica Protein, using ENSP00000000233.5 as a representative example. The "Data Resource" section outlines the raw datasets obtained from various databases utilized in protein entity integration. The "Data Cleaning" section highlights the standardized format of the data after preparation for integration. Key matching columns, marked in red boxes, were used to align data across sources. The "Data Merging" section visualizes the transformation of data formats through successive integration steps, represented by gray boxes. The integration process employs an outer join methodology, starting with the merging of Ensembl and UniProt databases. This combined dataset is then integrated with RefSeq. The final step uses the Ensembl stable ID version as the primary key to unify entries, ensuring that all records associated with the same Ensembl stable ID version are consolidated.

**Pathway Entities Integration** The data integration process began with Pathway Ontology (PO) as the foundational framework, merging datasets from PO, KEGG, and Reactome. Missing data was subsequently addressed through equivalent mapping relationships between KEGG and Reactome, as provided by ComPath. Finally, human pathway data from WikiPathway was integrated using equivalent mappings between KEGG and WikiPathway also facilitated by ComPath. Bolded columns in the table represent the fields used for merging with other databases, where the IDs in these columns are uniquely assigned (refer to **Figure S4** in the supplementary section for the detailed integration workflow and **Table S6** for results).

**Table S6**. Pathway Entity Information

| **Database** | | **Raw Data** | | **After Data Cleaning** | | Total Number of BioMedGraphica ID / BioMedGraphica Connected ID |
| --- | --- | --- | --- | --- | --- | --- |
|  |  | **Total Number of Rows** | **Unique** | **Total Number of Rows** | **Unique** |  |
| Pathway Ontology | PO ID | 2,677 | 2,677 | 2,677 | 2,677 | 6,793 / 1,930 |
|  | **KEGG ID** | 237 | 211 | 201 | 179 |  |
|  | Reactome ID | 327 | 321 | 327 | 321 |  |
|  | Total Number of Rows | 2677 | | 2677 | |  |
| KEGG | **KEGG ID** | 365 | 365 | 365 | 365 |  |
|  | Total Number of Rows | 365 | | 365 | |  |
| Reactome | **Reactome ID** | 2,751 | 2,751 | 2,751 | 2,751 |  |
|  | Total Number of Rows | 2751 | | 2751 | |  |
| ComPath | **KEGG ID** | 953 | 859 | 113 | 89 |  |
|  | **Reactome ID** | 1,274 | 1,055 | 58 | 57 |  |
|  | **WikiPathway ID** | 940 | 724 | 55 | 55 |  |
|  | Total Number of Rows | 1,592 | | 113 | |  |
| WikiPathways | **WikiPathways ID** | 1,534 | 1,534 | 1,534 | 1,534 |  |
|  | Total Number of Rows | 1,534 | | 1,534 | |  |

*represent this column contains multiple IDs in one row

Values separated by semicolons indicate data from multiple files


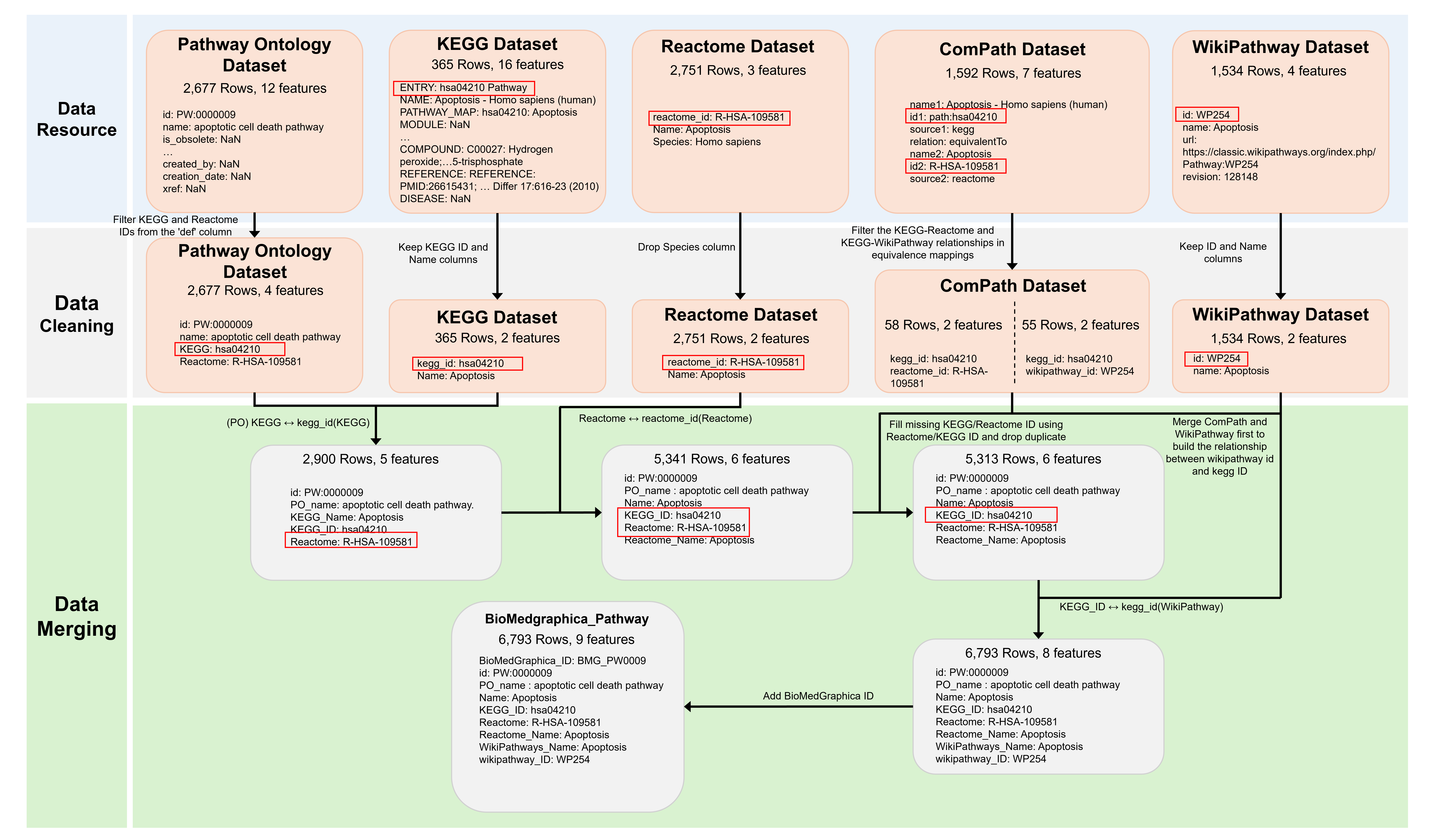


**Figure S4**. Details of Pathway Entity Merging Process

**Figure S4** provides a detailed illustration of the integration process for BioMedGraphica Pathway, using PW:0000009 as an example. The "Data Resource" section represents the raw data from the databases used in the integration of the pathway entity. The "Data Cleaning" section displays the format of the cleaned data prepared for integration. The data highlighted in the red boxes indicates the key matching columns used for merging. In the "Data Merging" section, the gray boxes show the format of the data after each step of database integration. The pathway entity integration process follows an outer join method. First, the Pathway Ontology and KEGG databases are merged, followed by the integration of Reactome data into the combined dataset, and subsequently ComPath and WikiPathway are integrated in sequence.

**Metabolite Entities Integration** The integration of the two databases utilized the ChEBI ID as the primary linking key. Subsequently, entries with identical HMDB IDs were consolidated, establishing the HMDB ID as the smallest unit of data granularity. Columns highlighted in bold within the table denote those used for database merging, ensuring the uniqueness of IDs in these columns (see **Figure S5** in the supplementary section for details on the merging process and **Table S7** for the results).

**Table S7**. Metabolite Entity Information

| **Database** | | **Raw Data** | | **After Data Cleaning** | | Total Number of BioMedGraphica ID / BioMedGraphica Connected ID |
| --- | --- | --- | --- | --- | --- | --- |
|  |  | **Total Number of Rows** | **Unique** | **Total Number of Rows** | **Unique** |  |
| HMDB | HMDB ID | 217,920 | 217,920 | 217,920 | 217,920 | 218,335 / 62,364 |
|  | CAS | 15,672 | 15,647 | 15,672 | 15,647 |  |
|  | ChemSpider ID | 31,269 | 31,015 | 31,269 | 31,015 |  |
|  | PubChem CID | 104,230 | 103,682 | 104,230 | 103,682 |  |
|  | **ChEBI ID** | 13,701 | 13,562 | 13,701 | 13,562 |  |
|  | PDB ID | 522 | 520 | 522 | 520 |  |
|  | KEGG ID | 6,814 | 5,908 | 6,814 | 5,908 |  |
|  | Total Number of Rows | 217,920 | | 217,920 | |  |
| ChEBI | **ChEBI ID** | 5,757; 392,733 | 5,757; 161,983 | 2,934 | 2,934 |  |
|  | CAS Number | NA; 29,147 | NA; 28,976 | 1,700 | 1,674* |  |
|  | HMDB ID | NA; 19,689 | NA; 19,274 | 2,203 | 2,171* |  |
|  | Total Number of Rows | 5,757; 392,733 | | 2,934 | |  |

*represent this column contains multiple IDs in one row

Values separated by semicolons indicate data from multiple files


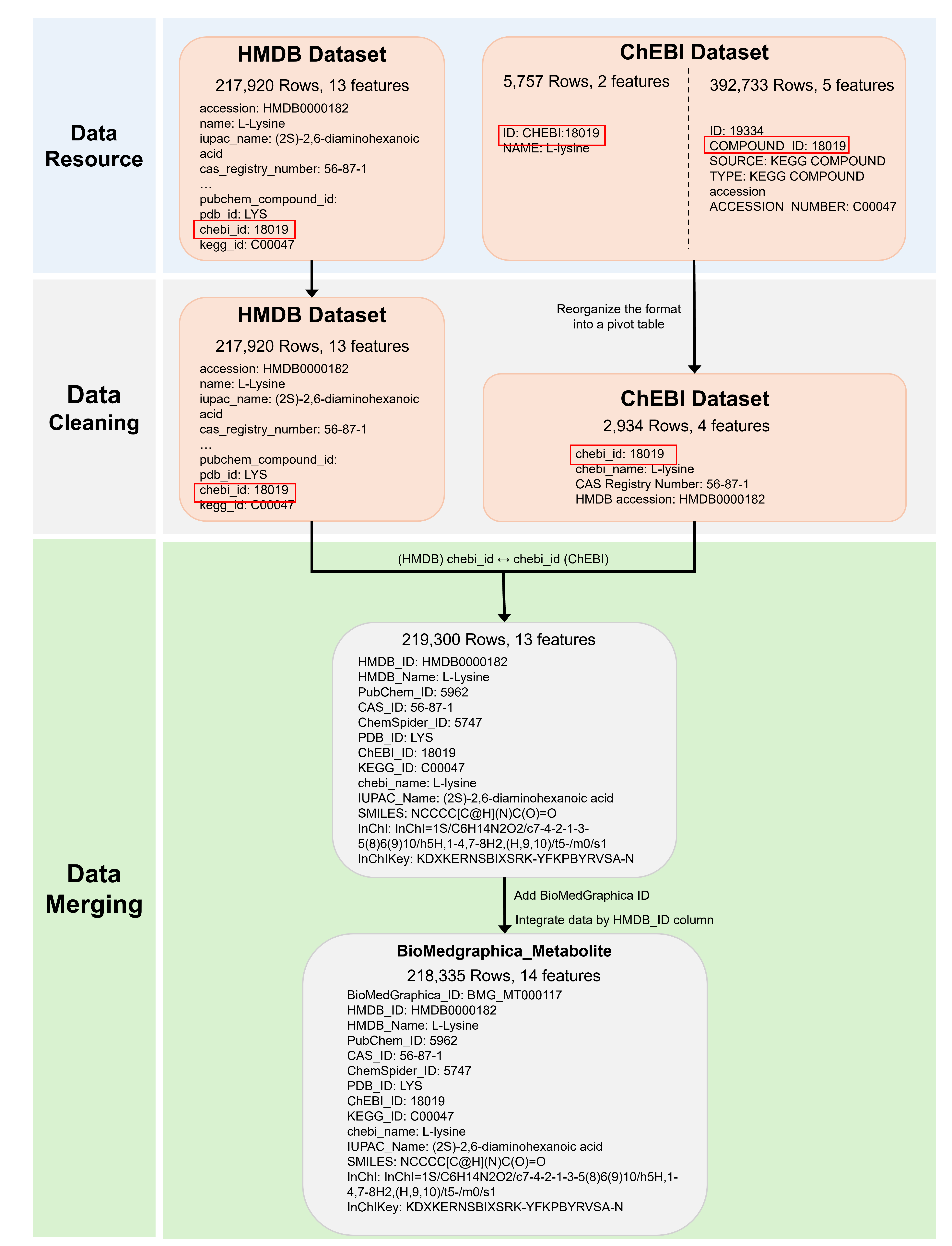


**Figure S5**. Details of Metabolite Entity Merging Process

**Figure S5** provides a detailed illustration of the integration process for BioMedGraphica Metabolite, using ChEBI: 18019 as an example. The "Data Resource" section represents the raw data from the databases used in the integration of the metabolite entity. The "Data Cleaning" section displays the format of the cleaned data prepared for integration. The data highlighted in the red boxes indicates the key matching columns used for merging. In the "Data Merging" section, the gray boxes show the data format after each step of database integration. The metabolite entity integration process follows an outer join method, merging data from the HMDB and ChEBI databases. Finally, the HMDB ID is used as the minimal unit for data unification, consolidating all entries with the same HMDB ID.

**Microbiota Entities Integration** The NCBI Taxon ID was employed as the standard key for harmonizing data across all microbiota datasets. This identifier was chosen due to its widespread presence in the included databases, enabling data merging. Columns highlighted in bold within the accompanying table indicate those used for cross-database integration, ensuring the uniqueness of IDs in these fields. For a detailed explanation of the integration methodology, refer to **Figure S6** in the supplementary section, with comprehensive results presented in **Table S8**.

**Table S8**. Microbiota Entities Information

| **Database** | | **Raw Data** | | **After Data Cleaning** | | Total Number of BioMedGraphica ID / BioMedGraphica Connected ID |
| --- | --- | --- | --- | --- | --- | --- |
|  |  | **Total Number of Rows** | **Unique** | **Total Number of Rows** | **Unique** |  |
| NCBI | **NCBI ID** | 2,631,459 | 2,631,459 | 538,194 | 538,194 | 621,882 / 1,119 |
|  | Total Number of Rows | 2,631,459 | | 538,194 | |  |
| SILVA | SILVA ID | 227,318; 2,224,690 | 157,873; 2,152,602 | 272,419 | 2,214,227* |  |
|  | **NCBI ID** | 227,318; 2,224,690 | 51,697; 267,817 | 272,419 | 272,419 |  |
|  | Total Number of Rows | 227,318; 2,224,690 | | 272,419 | |  |
| Greengene | Greengene ID | 1,144,866 | 1,144,866 | 92,684 | 1,144,866* |  |
|  | **NCBI ID** | 1,144,866 | 92,684 | 92,684 | 92,684 |  |
|  | RNAcentral ID | 1,144,866 | 1,004,892 | 92,684 | 1,004,892* |  |
|  | Total Number of Rows | 1,144,866 | | 92,684 | |  |
| RDP | RDP ID | 10,302 | 10,302 | 2,487 | 10,302* |  |
|  | **NCBI ID** | 10,302 | 2,487 | 2,487 | 2,487 |  |
|  | RNAcentral ID | 10,302 | 4,779 | 2,487 | 4,779* |  |
|  | Total Number of Rows | 10,302 | | 2,487 | |  |
| GTDB | GTDB ID | 12,477; 584,382 | 12,477; 584,382 | 92,444 | 596,859* |  |
|  | **NCBI ID** | 12,477; 584,382 | 2,768; 89,701 | 92,444 | 92,444 |  |
|  | Total Number of Rows | 12,477; 584,382 | | 92,444 | |  |

*represent this column contains multiple IDs in one row

Values separated by semicolons indicate data from multiple files


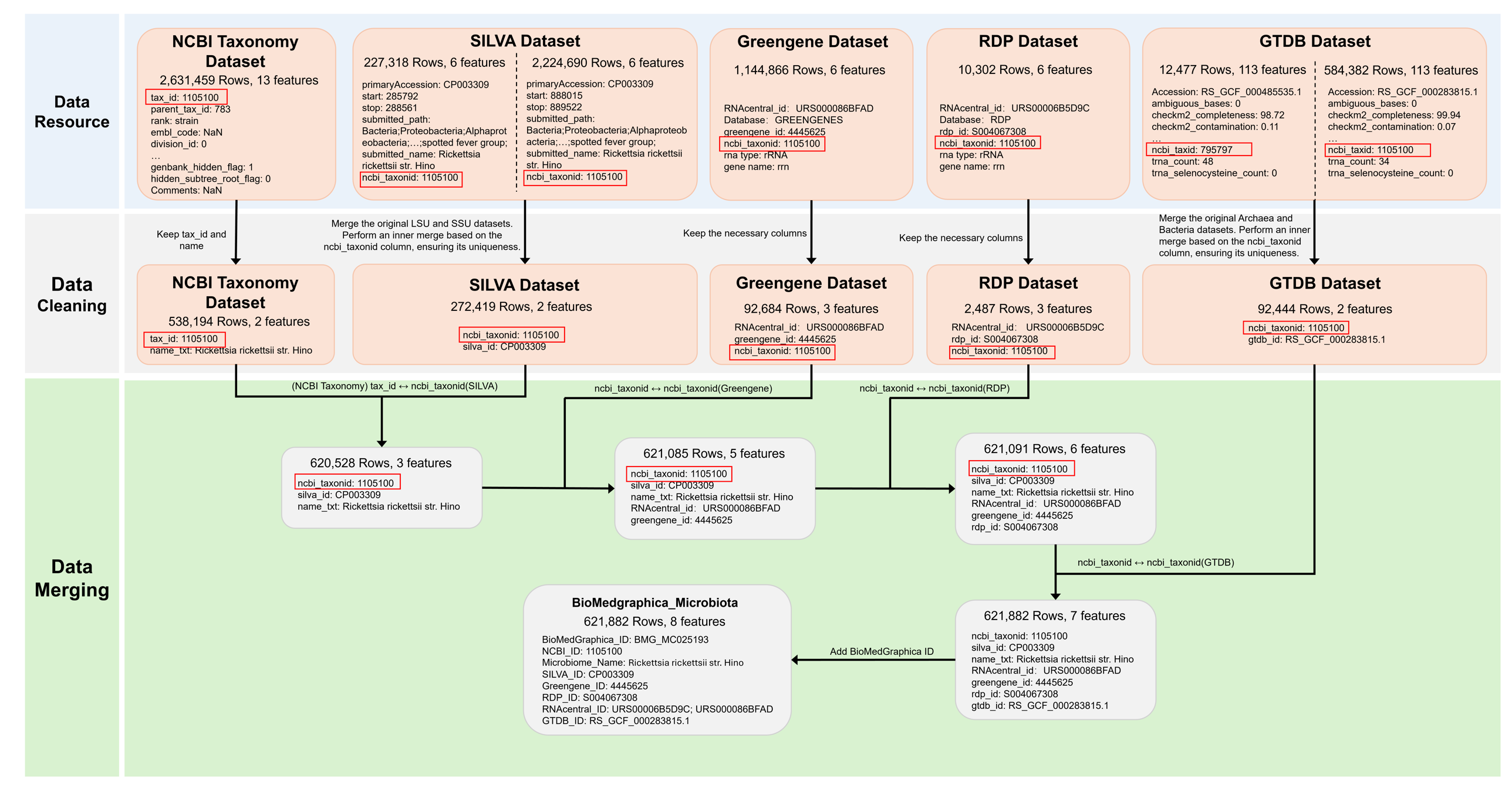


**Figure S6**. Details of Microbiota Entity Merging Process

**Figure S6** provides a detailed illustration of the integration process for BioMedGraphica Microbiota, using NCBI Taxon ID: 1105100 as an example. The "Data Resource" section represents the raw data from the databases used in the integration of the microbiota entity. The "Data Cleaning" section shows the format of the cleaned data prepared for integration. The data highlighted in the red boxes indicates the key matching columns used for merging. In the "Data Merging" section, the gray boxes display the data format after each step of database integration. The microbiota entity integration process follows an outer join strategy, first merging data from the NCBI Taxonomy and SILVA databases, followed by integration with Greengenes, RDP, and GTDB in sequence. Finally, the NCBI Taxon ID is used as the primary unit for data unification, consolidating all entries with the same NCBI Taxon ID.

**Exposure Entity Integration** The data integration for this entity was based on the CTD database. The construction of the exposure entity primarily depends on the Exposure-Study and Exposure-Event associations available in the Comparative Toxicogenomics Database (CTD). By extracting and integrating records based on the ExposureStressorID (corresponding to MeSH identifiers) from both datasets, an initial version of the exposure entity was assembled. Subsequently, each MeSH ID was annotated with its corresponding CSA RN, resulting in the finalized version of the entity. (check **Figure S7** in supplementary section for merging process and results in **Table S9**).

**Table S9**. Exposure Entities Information

| **Database** | | **Raw Data** | | **After Data Cleaning** | | Total Number of BioMedGraphica ID / BioMedGraphica Connected ID |
| --- | --- | --- | --- | --- | --- | --- |
|  |  | **Total Number of Rows** | **Unique** | **Total Number of Rows** | **Unique** |  |
| CTD | MeSH ID | 3,539; 224,304; 179,336 | 1,523; 1,159; 179,336 | 1,159 | 1,159 | 1,159 / 1,037 |
|  | CAS ID | NA; NA; 179,336 | NA; NA; 56,642 | 748 | 748 |  |
|  | Total Number of Rows | 3,539; 224,304; 179,336 | | 1,159 | |  |

*represent this column contains multiple IDs in one row

Values separated by semicolons indicate data from multiple files


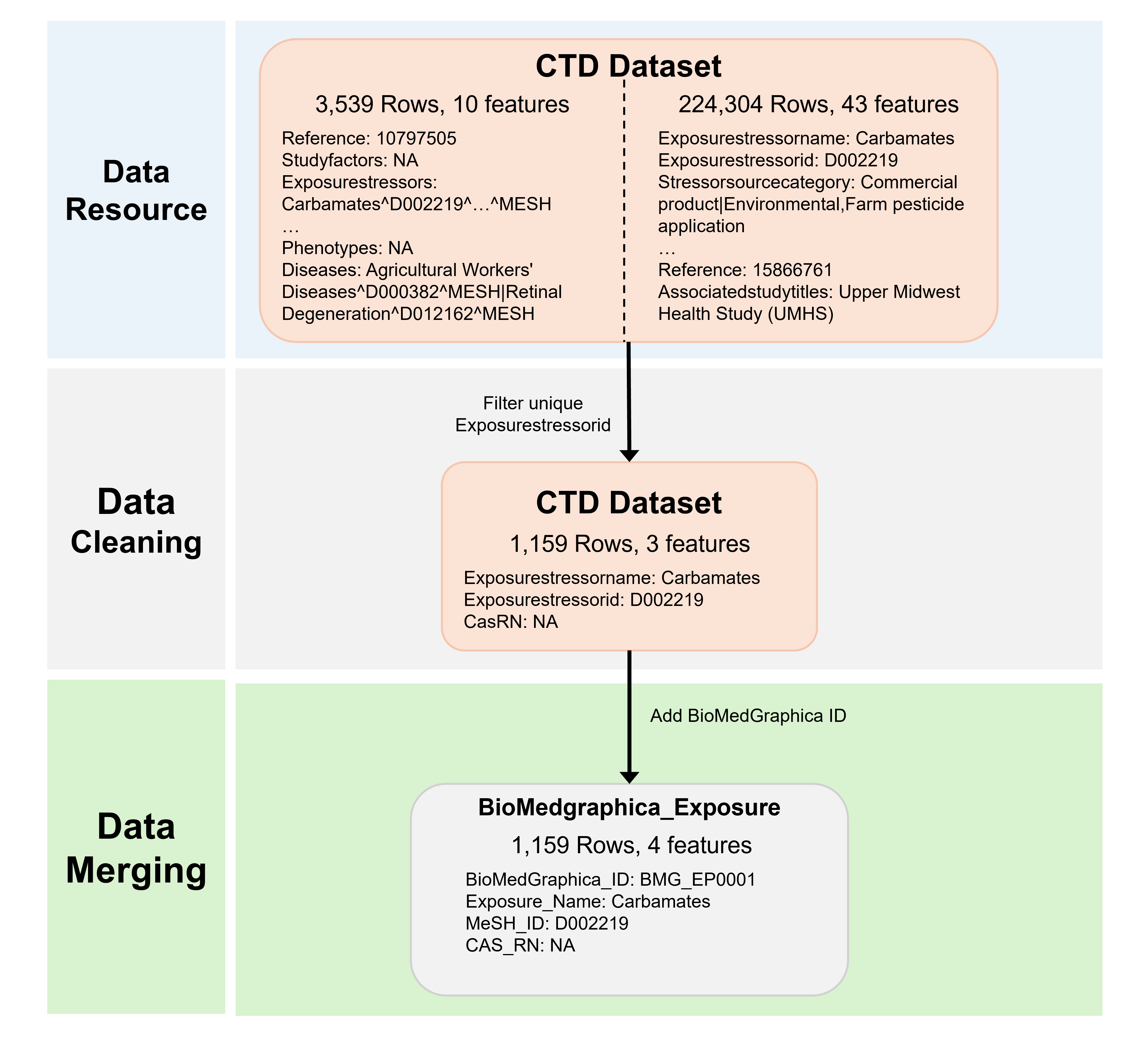


**Figure S7**. Details of Exposure Entity Merging Process

**Figure S7** uses MeSH ID D002219 as an example to illustrate the integration process for BioMedGraphica Exposure. The "Data Resource" section displays the raw data from databases used for the exposure entity. The "Data Cleaning" section shows the cleaned data format prepared for integration. The data highlighted in the red boxes indicates the key matching columns used for merging. In the "Data Merging" section, the gray boxes display the data format after each step of database integration.

**Phenotype Entity Merging** The integration of phenotype entities was performed using data from two primary sources: the Human Phenotype Ontology (HPO) and the Unified Medical Language System (UMLS). From the HPO dataset, we retained the HPO identifiers along with their cross-references to UMLS concepts; similarly, UMLS data were processed to extract corresponding mappings. The datasets were then merged using a left join based on the HPO identifiers, with HPO serving as the primary source. To ensure data consistency and avoid duplication, we further validated the uniqueness of each HPO ID in the resulting entity set. Columns highlighted in bold within the table denote those used for database merging, ensuring the uniqueness of IDs in these columns. (see **Figure S8** in the supplementary section for details on the merging workflow and **Table S10** for results).

**Table S10**. Phenotype entities database description

| **Database** | | **Raw Data** | | **After Data Cleaning** | | Total Number of BioMedGraphica ID / BioMedGraphica Connected ID |
| --- | --- | --- | --- | --- | --- | --- |
|  |  | **Total Number of Rows** | **Unique** | **Total Number of Rows** | **Unique** |  |
| HPO | **HPO ID** | 19,533 | 19,533 | 19,532 | 19,532 | 19,532 / 19,078 |
|  | UMLS ID | 19,532 | 12,786 | 19,532 | 12,786* |  |
|  | Total Number of Rows | 19,533 | | 19,532 | |  |
| UMLS | UMLS ID | 14,036,386 | 3,211,875 | 14,914 | 15,958* |  |
|  | **HPO ID** | 40,197 | 17,664 | 14,914 | 14,914 |  |
|  | Total Number of Rows | 14,036,386 | | 14,914 | |  |

*represent this column contains multiple IDs in one row


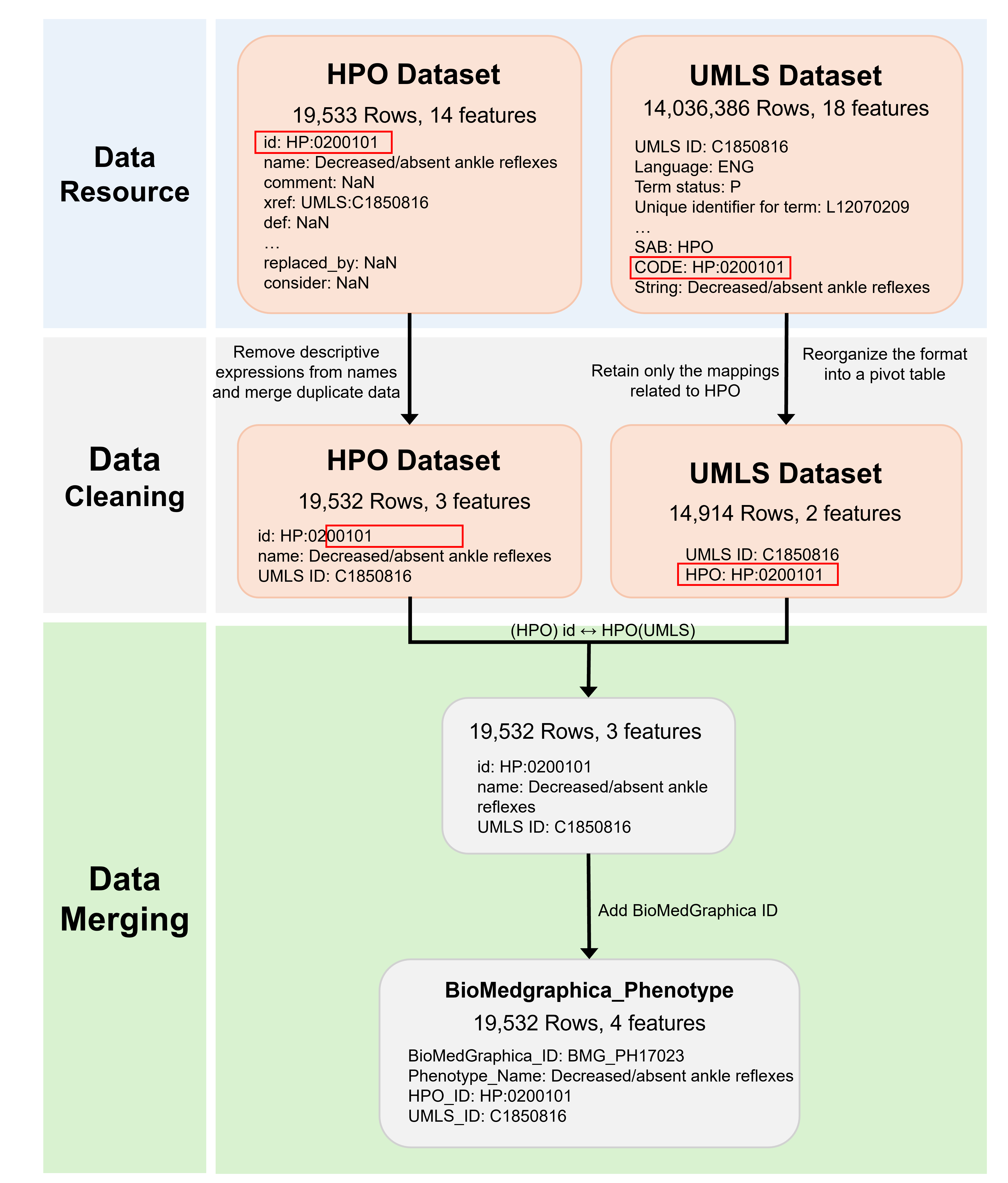


**Figure S8**. Details of Phenotype Entity Merging Process

**Figure S8** provides a detailed illustration of the integration process for BioMedGraphica Phenotype, using HP: 0200101 as an example. The "Data Resource" section represents the raw data from the databases used in the integration of the phenotype entity. The "Data Cleaning" section displays the format of the cleaned data prepared for integration. For HPO, descriptive terms in the original names were removed. The data highlighted in the red boxes indicates the key matching columns used for merging. In the "Data Merging" section, the gray boxes show the format of the data after each step of database integration. The phenotype entity integration process follows an outer join approach, merging data from the HPO and UMLS databases.

**Disease Entity Integration** The integration of disease entities began with the alignment of datasets from UMLS and MeSH. This was followed by the incorporation of mappings to ICD-10 and ICD-11, enabling further consolidation of disease codes. Using relationships provided by the Disease Ontology, identifiers from UMLS, MeSH, and ICD-10 were mapped to corresponding Disease Ontology (DO) terms to enrich the dataset with DO IDs. Missing UMLS data were supplemented via cross-references to SNOMED-CT. Finally, SNOMED-CT data were integrated to provide descriptive labels (names) for all previously collected SNOMED-CT identifiers; both SNOMED-CT IDs and their associated names were retained. Additionally, Mondo Disease Ontology data were merged using its mappings to UMLS and MeSH. Throughout the integration process, the UMLS ID served as the primary unit of granularity, ensuring unique identification across the dataset. Bolded columns in the accompanying table indicate fields used as primary keys during data merging; all identifiers in these fields are uniquely assigned. (refer to **Figure S9** in the supplementary section for a detailed workflow and **Table S11** for results).

**Table S11**. Disease Entity Information

| **Database** | | **Raw Data** | | **After Data Cleaning** | | Total Number of BioMedGraphica ID / BioMedGraphica Connected ID |
| --- | --- | --- | --- | --- | --- | --- |
|  |  | **Total Number of Rows** | **Unique** | **Total Number of Rows** | **Unique** |  |
| UMLS | UMLS ID | 16,704,679 | 3,426,808 | 69,361 | 69,361 | 118,814 / 22,429 |
|  | ICD10 | 166,556 | 97899 | 12,839 | 11,237* |  |
|  | **MeSH ID** | 1,028,724 | 354,901 | 10,174 | 7,826 |  |
|  | OMIM ID | 198,617 | 108,931 | 6,181 | 5,954* |  |
|  | SNOMEDCT ID | 1,618,997 | 523,051 | 44,458 | 44,316* |  |
|  | Total Number of Rows | 16,704,679 | | 69,361 | |  |
| MeSH | **MeSH ID** | 5,056 | 5,056 | 5,056 | 5,056 |  |
|  | Total Number of Rows | 5,056 | | 5,056 | |  |
| SnomedCT | **SnomedCT ID** | 1,677,259 | 1,677,259 | 527,311 | 527,311 |  |
|  | SnomedCT Name | 1,677,259 | 1,677,259 | 527,311 | 527,311 |  |
|  | Total Number of Rows | 1,677,259 | | 527,311 | |  |
| ICD11 | **ICD11 ID** | 34,663 | 34,663 | 34,663 | 34,663 |  |
|  | Total Number of Rows | 36,044 | | 34,663 | |  |
| ICD10 | **ICD10 ID** | 12,597 | 12,597 | 10,077 | 10,077 |  |
|  | ICD11 ID | 12,301 | 6,710 | 10,077 | 5,876* |  |
|  | Total Number of Rows | 12,597 | | 10,077 | |  |
| Disease Ontology | DO ID | 38,212 | 11,062 | 11,062 | 11,062 |  |
|  | **UMLS ID** | 7,000 | 6,955 | 6,390 | 6,955* |  |
|  | MeSH ID | 4,042 | 3,679 | 3,965 | 3,679* |  |
|  | ICD10 ID | 3,655 | 2,438 | 3,529 | 2,438* |  |
|  | OMIM ID | 6,094 | 6,066 | 5,589 | 6,066* |  |
|  | Total Number of Rows | 38,212 | | 11,062 | |  |
| Mondo | Mondo ID | 134,286 | 26,896 | 26,896 | 26,896 |  |
|  | **UMLS ID** | 21,203 | 21,203 | 21,202 | 21,202 |  |
|  | MeSH ID | 8,361 | 8,190 | 8,235 | 8,188* |  |
|  | OMIM ID | 9,952 | 9,952 | 9,858 | 9,837* |  |
|  | Total Number of Rows | 134,286 | | 26,896 | |  |

*represent this column contains multiple IDs in one row


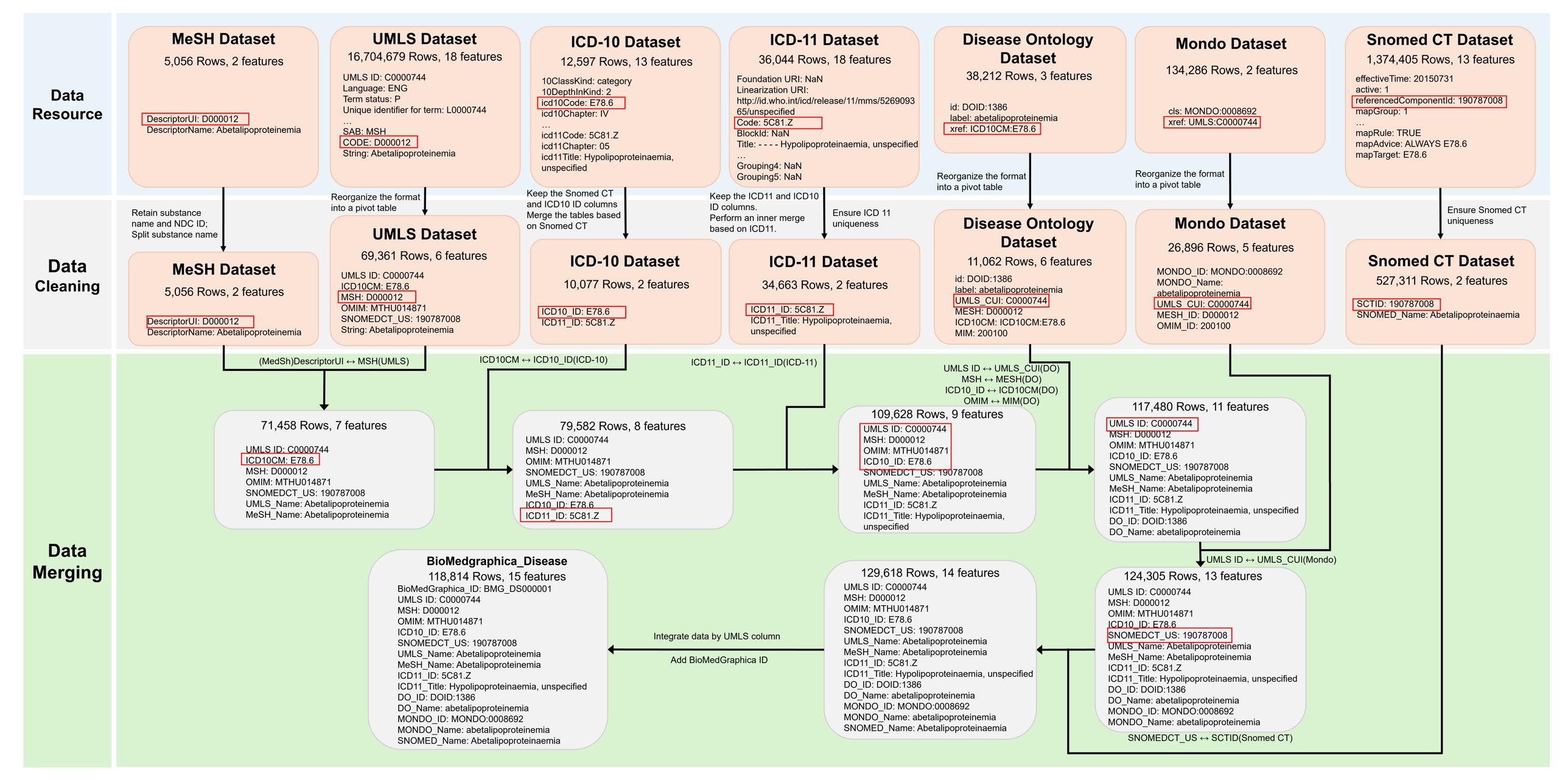


**Figure S9**. Details of Disease Entity Merging Process

**Figure S9** provides a detailed illustration of the integration process for BioMedGraphica Disease, using C0000744 as an example. The "Data Resource" section represents the raw data from the databases used in the integration of the disease entity. The "Data Cleaning" section shows the format of the cleaned data prepared for integration. The data highlighted in the red boxes indicates the key matching columns used for merging. In the "Data Merging" section, the gray boxes display the data format after each step of database integration. The disease entity integration process follows an outer join methodology. Initially, MeSH and UMLS databases were merged to construct the foundational dataset. This was followed by the sequential integration of ICD-10, ICD-11, Disease Ontology (DO), and Mondo, each contributing additional mappings and identifiers. In the final step, SNOMED CT data were incorporated to supplement the dataset with SNOMED CT identifiers and their corresponding concept names. Finally, the UMLS ID serves as the primary identifier for data unification, consolidating all entries with the same UMLS ID.

**Drug Entity Merging** The integration process commenced by merging NDC and UNII datasets using the SUBSTANCENAME as the key identifier. PubChem data was then incorporated through its mapping with PubChem CIDs. DrugBank data was integrated next, utilizing mappings between DrugBank IDs, CAS numbers, and SIDs. Finally, any missing data within the same row was supplemented using synonyms from both PubChem and DrugBank. After integration, internal deduplication was performed using PubChem CIDs and CAS RNs in sequence to ensure consistency. The CAS number was designated as the minimal unit of data granularity for this entity. Bolded entries in the table indicate the columns used for merging across databases, where IDs in these columns are uniquely assigned (refer to **Figure S10** in the supplementary section for details on the merging process and **Table S12** for the results).

**Table S12**. Drug Entity Information

| **Database** | | **Raw Data** | | **After Data Cleaning** | | Total Number of BioMedGraphica ID / BioMedGraphica Connected ID |
| --- | --- | --- | --- | --- | --- | --- |
|  |  | **Total Number of Rows** | **Unique** | **Total Number of Rows** | **Unique** |  |
| NDC | **UNII Name** | 105,815 | 9,189 | 8,125 | 7,311 | 273,386 / 20,918 |
|  | NDC ID | 107,980 | 107,980 | 8,125 | 104,850* |  |
|  | Total Number of Rows | 107,980 | | 8,125 | |  |
| UNII | UNII ID | 152,870; 159,376 | 152,870; 159,376 | 162,587 | 159,390 |  |
|  | **UNII Name** | 152,870; 159,376 | 152,870; 159,376 | 162,587 | 159,388 |  |
|  | PubChem CID | 112,013; 115,334 | 110,750; 115,266 | 121,638 | 120,449 |  |
|  | PubChem SID | 152,727; NA | 152,727; NA | 155,900 | 152,727 |  |
|  | CAS Number | NA; 120,704 | NA; 119,248 | 123,851 | 119,248 |  |
|  | Total Number of Rows | 152,870; 159,376 | | 162,587 | |  |
| PubChem | **PubChem CID** | 20,875; 115,017 | 20875; 115,017 | 123,357 | 123,357 |  |
|  | Total Number of Rows | 20,875; 115,017 | | 123,357 | |  |
| DrugBank | DrugBank ID | 17,430 | 17,430 | 17,430 | 17,430 |  |
|  | **CAS Number** | 10,767 | 10,734 | 10,767 | 10,734 |  |
|  | PubChem CID | 8,724 | 8,724 | 8,724 | 8,724 |  |
|  | **PubChem SID** | 10,450 | 10,450 | 10,450 | 10,450 |  |
|  | Total Number of Rows | 17,430 | | 17,430 | |  |

*represent this column contains multiple IDs in one row

Values separated by semicolons indicate data from multiple files


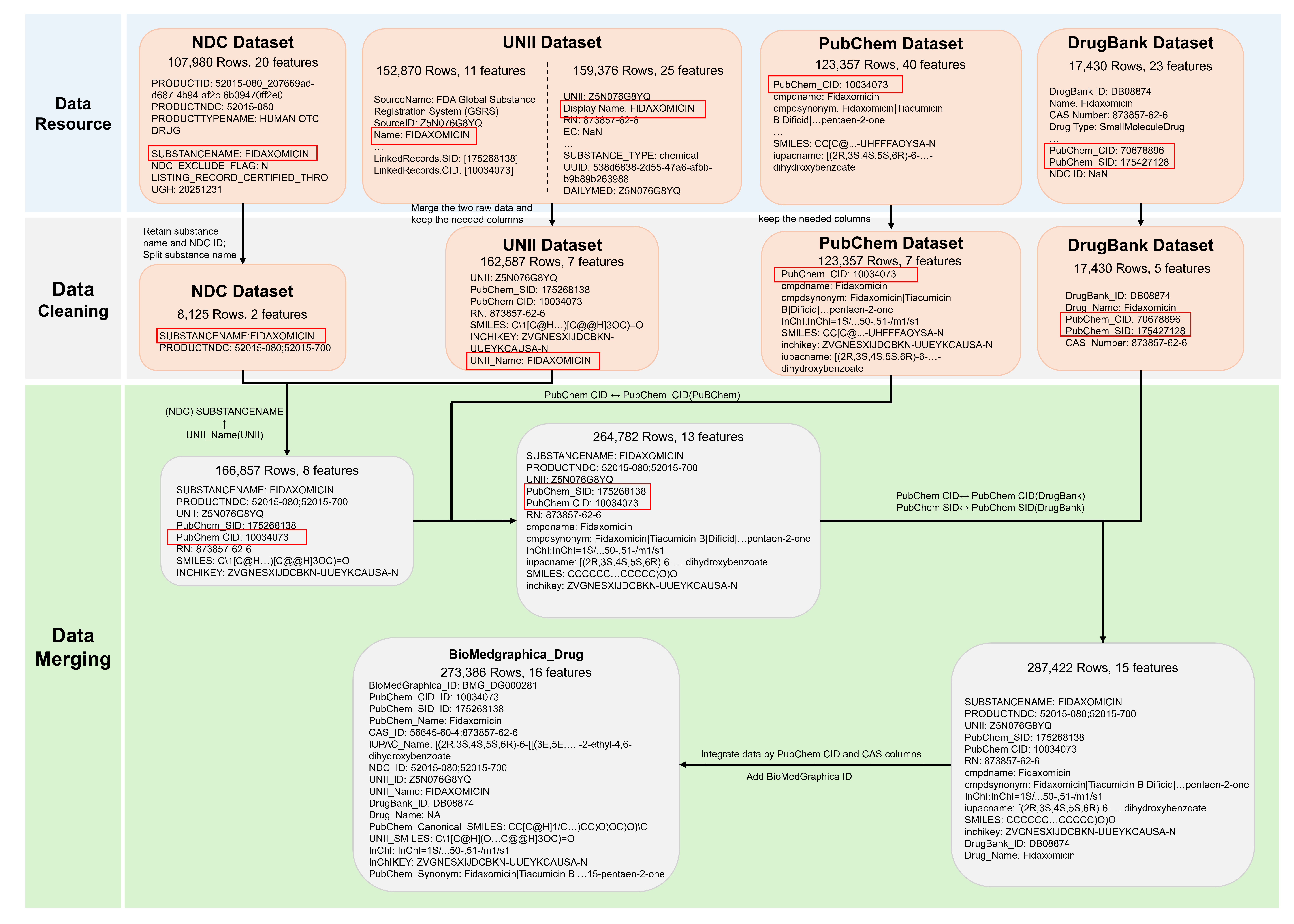


**Figure S10.** Details of Drug Entity Merging Process

**Figure S10** provides a detailed illustration of the integration process for BioMedGraphica Drug, using PubChem CID: 10034073 as an example. The "Data Resource" section represents the raw data from the databases used in the integration of the drug entity. The "Data Cleaning" section displays the format of the cleaned data prepared for integration. The data highlighted in the red boxes indicate the key matching columns used for merging. In the "Data Merging" section, the gray boxes show the format of the data after each database integration step. The drug entity integration process follows an outer join approach. First, the NDC and UNII databases are merged, followed by integrating the combined data with the PubChem database, and then DrugBank. Finally, the CAS number is used as the primary identifier for data unification, ensuring all entries with the same CAS number are consolidated.

**Section C. Matching Algorithm**

**C.1 Configurations of soft-matching algorithm**

For semantic soft matching between user-provided feature names and candidate entity aliases, we used the pre-trained BioBERT model (dmis-lab/biobert-base-cased-v1.2). Both query feature names and candidate aliases were encoded using the final hidden layer representations. Token embeddings were aggregated via mean pooling over non-padding tokens to obtain fixed-length sentence embeddings, followed by L2 normalization. Similarity between query embeddings and alias embeddings was computed using cosine similarity. In practice, this was implemented as inner-product search over normalized vectors using FAISS (IndexFlatIP) for efficient retrieval. Offline alias embeddings were precomputed with a batch size of 256 to improve runtime efficiency. The maximum sequence length was set to 64 tokens for candidate aliases and 128 tokens for query feature names. These values were selected to balance computational efficiency and input coverage, as most biomedical entity names and aliases are relatively short. For each query, top-ranked alias hits were aggregated at the entity level, and the top-k candidate entities were ranked based on aggregated similarity scores to construct the final mapping dictionary.

**C.2 Detailed performances of matching algorithms**

The overall performance of entity matching is reported in **Table S13**, and detailed results across all entity types are provided in Tables **S14-S23**, where the *Overall Matching Rate* is calculated as the total number of matched entities divided by the total number of original entities. Details of the collected datasets are presented in **Table S24**.

**Table S13.** Detailed of Matching Algorithm Performance on Promoter/Gene Entity Across Platforms

| **Entity** | **Hetionet** | **SPOKE** | **PrimeKG** | **BioMedGraphica** |
| --- | --- | --- | --- | --- |
| Promoter/Gene | 29.03% | 31.04% | 43.5% | 98.14% |
| Transcript | 37.46% | 39.83% | 52.42% | 94.06% |
| Protein | 83.55% | 98.2% | 87.82% | 92.47% |
| Pathway | 64.24% | 8.58% | 48.16% | 47.51% |
| Metabolite | N/A | N/A | N/A | 58.19% |
| Microbiota | N/A | 48.61% | N/A | 48.15% |
| Exposure | N/A | N/A | 54.55% | 100% |
| Phenotype | 29.63% | 70.37% | 55.56% | 92.59% |
| Disease | 26.09% | 73.91% | 52.17% | 91.30% |
| Drug | 84.62% | 96.15% | 96.15% | 96.15% |

**Table S14.** Detailed of Matching Algorithm Performance on Promoter/Gene Entity Across Platforms

| **File Name** | **Hetionet** | **SPOKE** | **PrimeKG** | **BioMedGraphica** |
| --- | --- | --- | --- | --- |
| APOLLO-LUAD.segment_cnv_ascat-ngs_ensg_id.json | 28.83% | 30.81% | 43.32% | 98.14% |
| TCGA-GBM.masked_cnv_DNAcopy_ensg_id.json | 29.21% | 31.24% | 43.59% | 98.14% |
| TCGA-LAML.allele_cnv_ascat3_ensg_id.json | 29.06% | 31.08% | 43.61% | 98.13% |
| Overall Matching Rate (95% CI) | 29.03% (95% CI 28.82%–29.24%) | 31.04% (95% CI 30.83%–31.25%) | 43.50% (95% CI 43.28%–43.73%) | 98.14% (95% CI 98.07%–98.20%) |

**Table S15.** Detailed of Matching Algorithm Performance on Transcript Entity Across Platforms

| **File Name** | **Hetionet** | **SPOKE** | **PrimeKG** | **BioMedGraphica** |
| --- | --- | --- | --- | --- |
| APOLLO-LUAD.star_counts_ensg_id.json | 29.76% | 31.81% | 44.56% | 97.96% |
| GSE164073_raw_counts_GRCh38.p13_NCBI_ensg_id.json | 66.68% | 70.91% | 83.65% | 99.44% |
| ROSMAP_RNAseq_FPKM_gene_ensg_id.json | 31.71% | 33.53% | 45.88% | 87.24% |
| Overall Matching Rate (95% CI) | 37.46% (95% CI 37.21%–37.71%) | 39.83% (95% CI 39.57%–40.08%) | 52.42% (95% CI 52.16%–52.68%) | 94.06% (95% CI 93.94%–94.18%) |

**Table S16.** Detailed of Matching Algorithm Performance on Protein Entity Across Platforms

| **File Name** | **Hetionet** | **SPOKE** | **PrimeKG** | **BioMedGraphica** |
| --- | --- | --- | --- | --- |
| A1.MaxQuant-LFQ intensity-4698x383TMTchannels-no_batch_correction_uniprot_id.json | 90.14% | 98.98% | 95.05% | 96.93% |
| A1.PD-RAW_normalized_abundance-11672x500TMTchannels-no_batch_correction_uniprot_id.json | 79.28% | 97.68% | 83.60% | 89.85% |
| dicad_proteomics_july2020_uniprot_id.json | 98.37% | 100.00% | 99.83% | 100.00% |
| Overall Matching Rate (95% CI) | 83.55% (95% CI 82.98%–84.10%) | 98.20% (95% CI 97.98%–98.39%) | 87.82% (95% CI 87.32%–88.31%) | 92.47% (95% CI 92.06%–92.86%) |

**Table S17.** Detailed of Matching Algorithm Performance on Pathway Entity Across Platforms

| **File Name** | **Hetionet** | **SPOKE** | **PrimeKG** | **BioMedGraphica** |
| --- | --- | --- | --- | --- |
| UCEC_PanCan33_ssGSEA_1387GeneSets_NonZero_  sample_level_Z_reactome_name. json | 64.24% | 8.58% | 48.16% | 47.51% |
| Overall Matching Rate (95% CI) | 64.24% (95% CI 61.68%–66.72) | 8.58% (95% CI 7.22%–10.17%) | 48.16% (95% CI 45.54%–50.79%) | 47.51% (95% CI 44.90%–50.14%) |

**Table S18.** Detailed of Matching Algorithm Performance on Metabolite Entity Across Platforms

| **File Name** | **Hetionet** | **SPOKE** | **PrimeKG** | **BioMedGraphica** |
| --- | --- | --- | --- | --- |
| m_MTBLS13855_LC-MS_positive_reverse-phase_metabolite_profiling_v2_maf_hmdb_name.json | N/A | N/A | N/A | 55.07% |
| m_MTBLS13878_LC-MS_positive_reverse-phase_metabolite_profiling_v2_maf_hmdb_name.json | N/A | N/A | N/A | 58.65% |
| m_MTBLS13881_LC-MS_positive_reverse-phase_metabolite_profiling_v2_maf_hmdb_name.json | N/A | N/A | N/A | 69.05% |
| Overall Matching Rate (95% CI) | N/A | N/A | N/A | 58.19% (95% CI 55.36%–60.96%) |

**Table S19.** Detailed of Matching Algorithm Performance on Microbiota Entity Across Platforms

| **File Name** | **Hetionet** | **SPOKE** | **PrimeKG** | **BioMedGraphica** |
| --- | --- | --- | --- | --- |
| ERR133619_SFF_otu_taxonomy_name.json | N/A | 41.03% | N/A | 40.17% |
| ERR208341_FASTQ_otu_taxonomy_name.json | N/A | 100.00% | N/A | 100.00% |
| ERR599089_MERGED_FASTQ_  otu_taxonomy_name.json | N/A | 55.32% | N/A | 55.32% |
| Overall Matching Rate (95% CI) | N/A | 48.61% (95% CI 42.03%–55.24%) | N/A | 48.15% (95% CI 41.58%–54.79%) |

**Table S20.** Detailed of Matching Algorithm Performance on Exposure Entity Across Platforms

| **File Name** | **Hetionet** | **SPOKE** | **PrimeKG** | **BioMedGraphica** |
| --- | --- | --- | --- | --- |
| ep1.json | N/A | N/A | 42.86% | 100.00% |
| ep2.json | N/A | N/A | 62.50% | 100.00% |
| ep3.json | N/A | N/A | 57.14% | 100.00% |
| Overall Matching Rate (95% CI) | N/A | N/A | 54.55% (95% CI 34.66%–73.08%) | 100.00% (95% CI 85.13%–100.00%) |

**Table S21.** Detailed of Matching Algorithm Performance on Phenotype Entity Across Platforms

| **File Name** | **Hetionet** | **SPOKE** | **PrimeKG** | **BioMedGraphica** |
| --- | --- | --- | --- | --- |
| ph1.json | 33.33% | 66.67% | 44.44% | 88.89% |
| ph2.json | 33.33% | 77.78% | 66.67% | 88.89% |
| ph3.json | 22.22% | 66.67% | 55.56% | 100.00% |
| Overall Matching Rate (95% CI) | 29.63% (95% CI 15.85%–48.48%) | 70.37% (95% CI 51.52%–84.15%) | 55.56% (95% CI 37.31%–72.41%) | 92.59% (95% CI 76.63%–97.94%) |

**Table S22.** Detailed of Matching Algorithm Performance on Disease Entity Across Platforms

| **File Name** | **Hetionet** | **SPOKE** | **PrimeKG** | **BioMedGraphica** |
| --- | --- | --- | --- | --- |
| ds1.json | 0.00% | 66.67% | 44.44% | 88.89% |
| ds2.json | 33.33% | 66.67% | 33.33% | 100.00% |
| ds3.json | 50.00% | 87.50% | 75.00% | 87.50% |
| Overall Matching Rate (95% CI) | 26.09% (95% CI 12.55%–46.47%) | 73.91 (95% CI 53.53%–87.45%) | 52.17% (95% CI 32.96%–70.76%) | 91.30% (95% CI 73.20%–97.58%) |

**Table S23.** Detailed of Matching Algorithm Performance on Drug Entity Across Platforms

| **File Name** | **Hetionet** | **SPOKE** | **PrimeKG** | **BioMedGraphica** |
| --- | --- | --- | --- | --- |
| dg1.json | 87.50% | 100.00% | 100.00% | 100.00% |
| dg2.json | 88.89% | 88.89% | 88.89% | 88.89% |
| dg3.json | 77.78% | 100.00% | 100.00% | 100.00% |
| Overall Matching Rate (95% CI) | 84.62% (95% CI 66.47%–93.85%) | 96.15% (95% CI 81.11%–99.32%) | 96.15% (95% CI 81.11%–99.32%) | 96.15% (95% CI 81.11%–99.32%) |

**Table S24.** Details of Collected Datasets

| **Entity** | **Source** | **Dataset** | **ID Type** | **Download Link** | **Renamed Filename** | **Notes** |
| --- | --- | --- | --- | --- | --- | --- |
| Promoter/ Gene | GDC Xena Hub | GDC APOLLO-LUAD | Ensembl Gene ID | https://gdc-hub.s3.us-east-1.amazonaws.com/download/APOLLO-LUAD.segment_cnv_ascat-ngs.tsv.gz | APOLLO-LUAD.segment_cnv_ascat-ngs_ensg_id.json | Converted from chromosome coordinates (chrom, start, end) to Ensembl Gene ID using pyensembl |
|  |  | GDC TCGA Glioblastoma (GBM) |  | https://gdc-hub.s3.us-east-1.amazonaws.com/download/TCGA-GBM.masked_cnv_DNAcopy.tsv.gz | TCGA-GBM.masked_cnv_DNAcopy_ensg_id.json |  |
|  |  | GDC TCGA Acute Myeloid Leukemia (LAML) |  | https://gdc-hub.s3.us-east-1.amazonaws.com/download/TCGA-LAML.allele_cnv_ascat3.tsv.gz | TCGA-LAML.allele_cnv_ascat3_ensg_id.json |  |
| Transcript | GDC Xena Hub | GDC APOLLO-LUAD | Ensembl Gene ID | https://gdc-hub.s3.us-east-1.amazonaws.com/download/APOLLO-LUAD.star_counts.tsv.gz | APOLLO-LUAD.star_counts_ensg_id.json |  |
|  | NCBI Gene Expression Omnibus | GSE164073 |  | https://www.ncbi.nlm.nih.gov/geo/  download/?type=rnaseq_counts&acc  =GSE164073&format=file&file  =GSE164073_raw_counts  _GRCh38.p13_NCBI.tsv.gz | GSE164073_raw_counts_GRCh38.p13_NCBI_ensg_id.json | Gene identifier conversion from NCBI Gene ID to Ensembl Gene ID was performed using the human genome annotation table (Human.GRCh38.p13.annot.tsv). |
|  | AD Knowledge Portal | ROSMAP |  | https://www.synapse.org/Synapse:syn3505720 | ROSMAP_RNAseq_FPKM_gene_ensg_id.json |  |
| Protein | AD Knowledge Portal | UPP | Uniprot ID | https://www.synapse.org/Synapse:syn21437077 | A1.MaxQuant-LFQ intensity-4698x383TMTchannels-no_batch_correction_uniprot_id.json |  |
|  |  | ROSMAP |  | https://www.synapse.org/Synapse:syn21266452 | A1.PD-RAW_normalized_abundance-11672x500TMTchannels-no_batch_correction_uniprot_id.json |  |
|  |  | DiCAD |  | https://www.synapse.org/Synapse:syn25178485 | dicad_proteomics_july2020_uniprot_id.json |  |
| Pathway | GDC Xena Hub | UCEC | Reactome Name | https://tcga-xena-hub.s3.us-east-1.amazonaws.com/download  /PanCan33_ssGSEA_1387GeneSets_  NonZero_sample_level_Z%2FUCEC_PanCan33_ssGSEA_1387GeneSets_NonZero_sample_level_Z.txt.gz | UCEC_PanCan33_ssGSEA_1387GeneSets_NonZero_sample_level_Z_reactome_name.json |  |
| Metabolite | MetaboLights | MTBLS13855 | HMDB Name | https://www.ebi.ac.uk/metabolights  /editor/MTBLS13855/metabolites | m_MTBLS13855_LC-MS_positive_reverse-phase_metabolite_profiling_v2_maf_hmdb_name.json |  |
|  |  | MTBLS13878 |  | https://www.ebi.ac.uk/metabolights  /editor/MTBLS13878/metabolites | m_MTBLS13878_LC-MS_positive_reverse-phase_metabolite_profiling_v2_maf_hmdb_name.json |  |
|  |  | MTBLS13881 |  | https://www.ebi.ac.uk/metabolights  /editor/MTBLS13881/metabolites | m_MTBLS13881_LC-MS_positive_reverse-phase_metabolite_profiling_v2_maf_hmdb_name.json |  |
| Microbiota | MGnify | MGYS00000505 | Taxonomy Name | https://www.ebi.ac.uk/metagenomics/api/v1/analyses/MGYA00008638/file/ERR133619_SFF_otu.tsv | ERR133619_SFF_otu_taxonomy_name.json |  |
|  |  | MGYS00000318 |  | https://www.ebi.ac.uk/metagenomics/api/v1/analyses/MGYA00000559/file/ERR208341_FASTQ_otu.tsv | ERR208341_FASTQ_otu_taxonomy_name.json |  |
|  |  | MGYS00000410 |  | https://www.ebi.ac.uk/metagenomics/api/v1/analyses/MGYA00005112/file/ERR599089_MERGED_FASTQ_otu.tsv | ERR599089_MERGED_FASTQ_otu_taxonomy_name.json |  |
| Exposure | LLM-generated | Air pollutant exposure | Exposure Name | N/A | ep1.json | Entity data were generated using ChatGPT 5.2 Thinking, and the source files are available at:<https://drive.google.com/file/d/1ATd3IUzTnAkx80pcb2kBF7DK-5cMSzdY/view?usp=sharing> |
|  |  | Environmental toxicant exposure |  |  | ep2.json |  |
|  |  | Pesticide and chemical exposure |  |  | ep3.json |  |
| Phenotype |  | Neurological phenotype | Phenotype Name |  | ph1.json |  |
|  |  | Cardiopulmonary phenotype |  |  | ph2.json |  |
|  |  | Multisystem phenotype |  |  | ph3.json |  |
| Disease |  | Oncologic disease | Disease Name |  | ds1.json |  |
|  |  | Cardiometabolic disease |  |  | ds2.json |  |
|  |  | Immune and respiratory disease |  |  | ds3.json |  |
| Drug |  | Cardiometabolic therapeutic drugs | Drug Name |  | dg1.json |  |
|  |  | Anti-infective drugs |  |  | dg2.json |  |
|  |  | Oncology and neuropsychiatric drugs |  |  | dg3.json |  |

**Section D. BioMedGraphica Web Application**

**D.1 Case study of using BioMedGraphica platform**

**
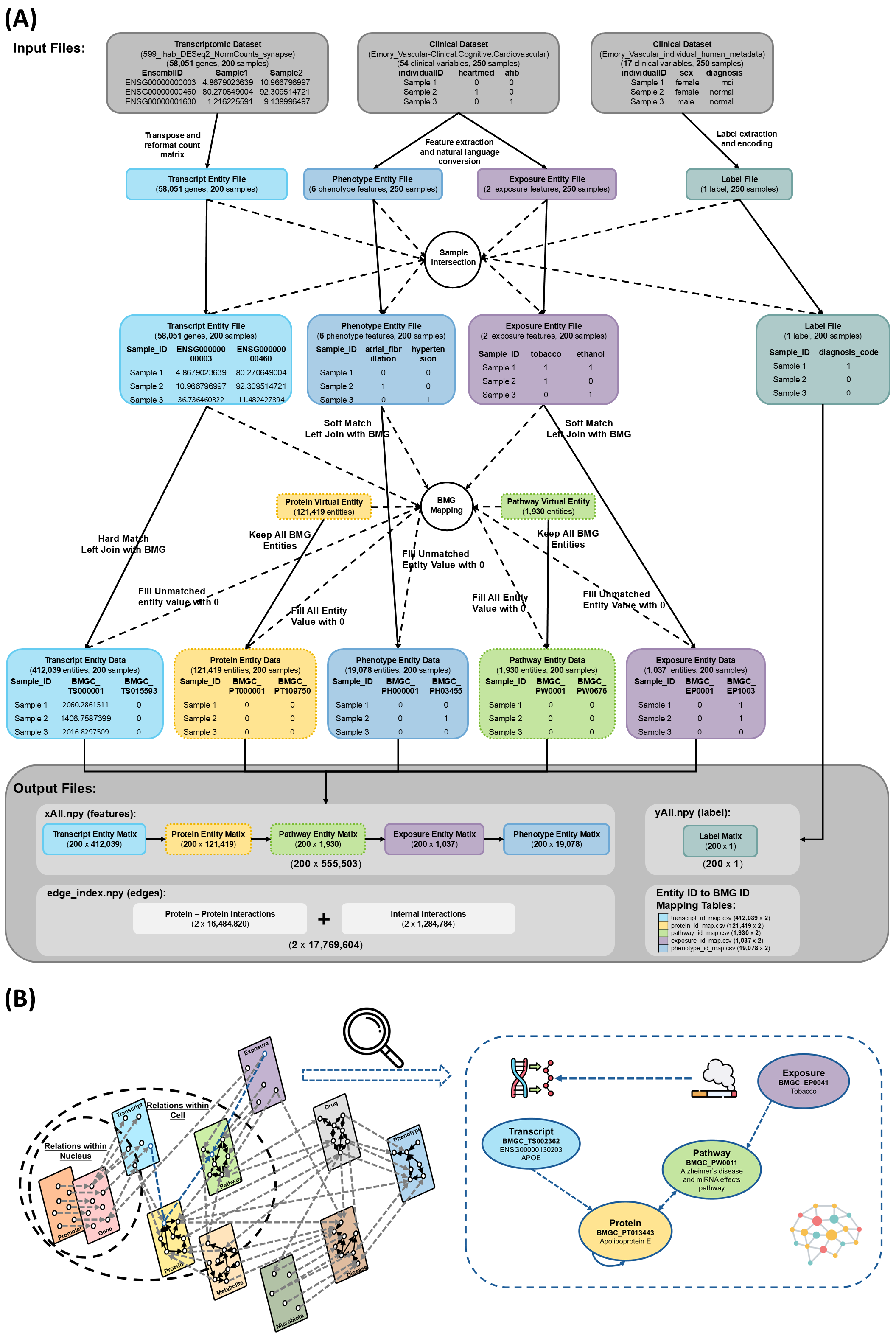
**

**Figure S11. TNG construction and representative biological connectivity in the Emory_Vascular case study. (A)** Overview of the BioMedGraphica pipeline for constructing a textual-numeric-graph (TNG) from transcriptomic, clinical, and diagnostic-label inputs. Transcript entities are generated from normalized transcript count data, Phenotype and Exposure entities are generated from subject-level clinical variables, and diagnosis information is encoded as graph labels. Protein and Pathway virtual entities derived from prior biological knowledge are introduced to restore biologically meaningful connectivity. The resulting outputs include entity matrices, labels, graph edges, and entity-to-BioMedGraphica ID mappings. **(B)** A representative connectivity example in the generated TNG, where the transcript node BMGC_TS002362 (ENSG00000130203, APOE) is linked to the exposure node BMGC_EP0041 (Tobacco) through the protein node BMGC_PT013443 (Apolipoprotein E) and the pathway node BMGC_PW0011 (Alzheimer’s disease and miRNA effects pathway). This example demonstrates the interpretability of BioMedGraphica for linking molecular features and clinically relevant variables in prodromal Alzheimer’s disease.

**Figure S11** provides an overview of the TNG construction workflow and a representative connectivity example in the Emory_Vascular case study, illustrating how transcriptomic, clinical, and diagnostic-label data are transformed into graph inputs, mapped to BioMedGraphica entities, and integrated with virtual nodes to restore biologically meaningful connectivity.

**D.2 Details of usage for BioMedGraphica web application**

**
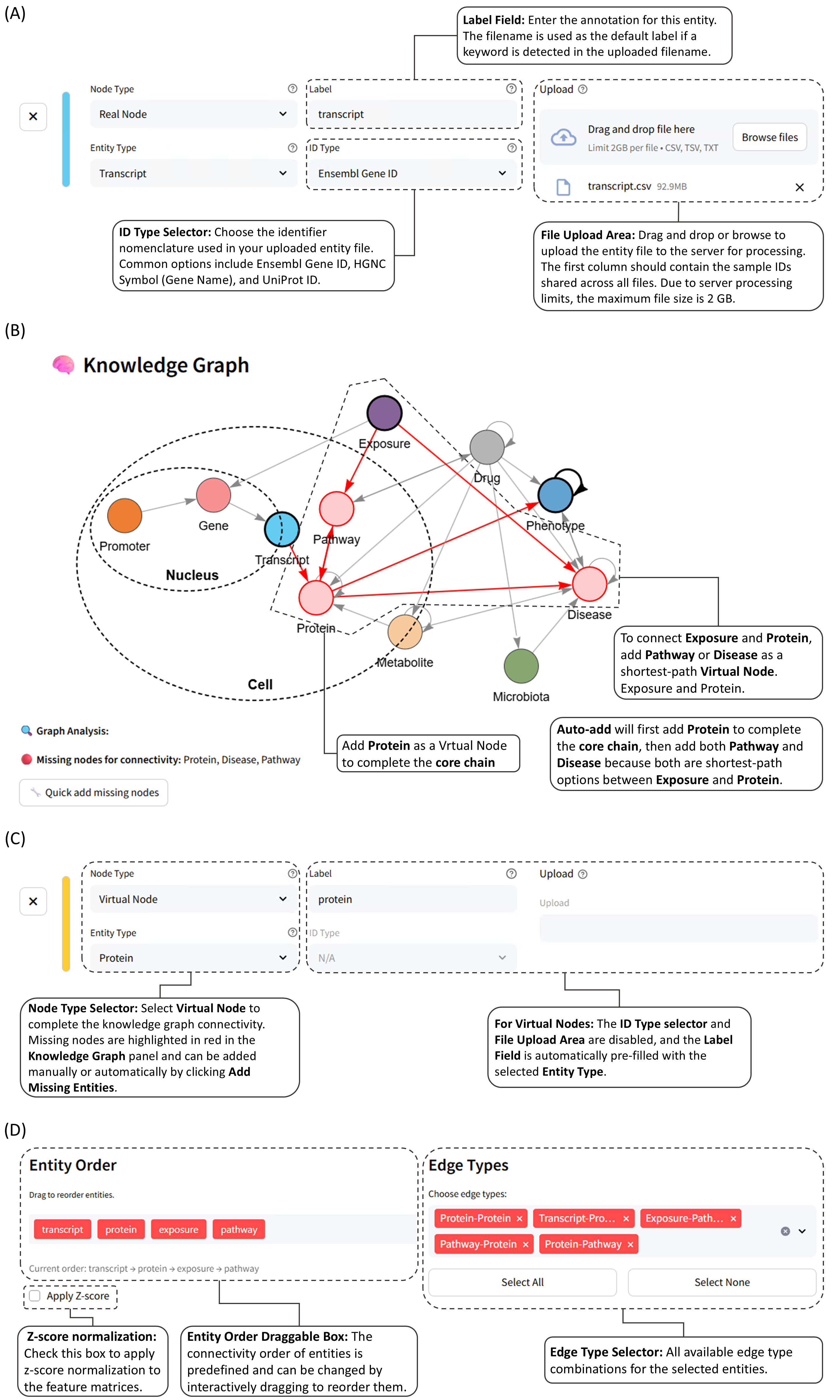
**

**Figure S12. Functional overview of major components in the web application interface.**
**(A)** *Real node upload panel.* Users specify the node category, entity class, display label, and identifier schema prior to uploading the entity file, where the first column contains shared sample identifiers. **(B)** *Knowledge graph visualization with missing-entity diagnostics.* Entity types required to maintain graph connectivity are flagged in red, enabling users to supplement them manually or via the quick-add utility. **(C)** *Virtual node upload panel.* For virtual nodes, the identifier selector and file upload module are disabled, and the display label is automatically populated according to the chosen entity class. **(D)** *Configuration panel.* This module supports entity ordering, selection of valid edge-type combinations, and optional z-score normalization of feature matrices.

**Figure S12** provides a functional overview of the major modules in the BioMedGraphica web interface, illustrating how users upload real and virtual nodes, diagnose missing entities to ensure graph connectivity, and configure entity ordering, edge selection, and feature normalization.

**References**

Amberger, Joanna, et al. (2009), ‘McKusick’s Online Mendelian Inheritance in Man (OMIM®)’, *Nucleic Acids Research*, 37/SUPPL. 1, https://doi.org/10.1093/nar/gkn665.

Bodenreider, Olivier (2004), ‘The Unified Medical Language System (UMLS): Integrating Biomedical Terminology’, *Nucleic Acids Research*, 32/suppl_1: D267–70.

Cole, James R., et al. (2014), ‘Ribosomal Database Project: Data and Tools for High Throughput RRNA Analysis’, *Nucleic Acids Research*, 42/D1: D633–42.

Davis, Allan Peter, et al. (2021), ‘Comparative Toxicogenomics Database (CTD): Update 2021’, *Nucleic Acids Research*, 49/D1: D1138–43, https://doi.org/10.1093/nar/gkaa891.

Degtyarenko, Kirill, et al. (2008), ‘ChEBI: A Database and Ontology for Chemical Entities of Biological Interest’, *Nucleic Acids Research*, 36/SUPPL. 1, https://doi.org/10.1093/nar/gkm791.

DeSantis, Todd Z., et al. (2006), ‘Greengenes, a Chimera-Checked 16S RRNA Gene Database and Workbench Compatible with ARB’, *Applied and Environmental Microbiology*, 72/7: 5069–72.

Doestzada, Marwah, et al. (2018), ‘Pharmacomicrobiomics: A Novel Route towards Personalized Medicine?’, *Protein & Cell*, 9/5: 432–45.

Domingo-Fernández, Daniel, et al. (2018), ‘ComPath: An Ecosystem for Exploring, Analyzing, and Curating Mappings across Pathway Databases’, *NPJ Systems Biology and Applications*, 4/1: 43.

Donnelly, Kevin (2006), ‘SNOMED-CT: The Advanced Terminology and Coding System for EHealth’, *Studies in Health Technology and Informatics*, 121: 279.

Fabregat, Antonio, et al. (2018), ‘The Reactome Pathway Knowledgebase’, *Nucleic Acids Research*, 46/D1: D649–55.

Gilson, Michael K., et al. (2016), ‘BindingDB in 2015: A Public Database for Medicinal Chemistry, Computational Chemistry and Systems Pharmacology’, *Nucleic Acids Research*, 44/D1: D1045–53.

Howe, Kevin L., et al. (2021), ‘Ensembl 2021’, *Nucleic Acids Research*, 49/D1: D884–91, https://doi.org/10.1093/nar/gkaa942.

Janssens, Yorick, et al. (2018), ‘Disbiome Database: Linking the Microbiome to Disease’, *BMC Microbiology*, 18: 1–6.

Kanehisa, M. G. S., and Susumu Goto (2000), ‘KEGG: Kyoto Encyclopedia of Genes and Genomes’, *Nucleic Acids Research*, 28: 27–30, https://doi.org/10.1093/nar/28.1.27.

Kelder, Thomas, et al. (2012), ‘WikiPathways: Building Research Communities on Biological Pathways’, *Nucleic Acids Research*, 40/D1: D1301–7.

Knox, Craig, et al. (2024), ‘DrugBank 6.0: The DrugBank Knowledgebase for 2024’, *Nucleic Acids Research*, 52/D1: D1265–75, https://doi.org/10.1093/nar/gkad976.

Köhler, Sebastian, et al. (2021), ‘The Human Phenotype Ontology in 2021’, *Nucleic Acids Research*, 49/D1: D1207–17.

Kuhn, Michael, et al. (2010), ‘A Side Effect Resource to Capture Phenotypic Effects of Drugs’, *Molecular Systems Biology*, 6/1: 343.

Lipscomb, Carolyn E. (2000), ‘Medical Subject Headings (MeSH)’, *Bulletin of the Medical Library Association*, 88/3: 265.

Moretti, Sébastien, et al. (2021), ‘MetaNetX/MNXref: Unified Namespace for Metabolites and Biochemical Reactions in the Context of Metabolic Models’, *Nucleic Acids Research*, 49/D1: D570–4.

O’Leary, Nuala A., et al. (2016), ‘Reference Sequence (RefSeq) Database at NCBI: Current Status, Taxonomic Expansion, and Functional Annotation’, *Nucleic Acids Research*, 44/D1: D733–45, https://doi.org/10.1093/nar/gkv1189.

Organization, World Health (2004), *International Statistical Classification of Diseases and Related Health Problems: Alphabetical Index*, vol. 3 (n.p.).

Organization, World Health (2018), ‘International Classification of Diseases for Mortality and Morbidity Statistics (11th Revision)’, preprint.

Oughtred, Rose, et al. (2019), ‘The BioGRID Interaction Database: 2019 Update’, *Nucleic Acids Research*, 47/D1: D529–41.

Parks, Donovan H., et al. (2018), ‘A Standardized Bacterial Taxonomy Based on Genome Phylogeny Substantially Revises the Tree of Life’, *Nature Biotechnology*, 36/10: 996–1004.

Petri, Victoria, et al. (2014), ‘The Pathway Ontology–Updates and Applications’, *Journal of Biomedical Semantics*, 5: 1–12.

Piñero, Janet, et al. (2016), ‘DisGeNET: A Comprehensive Platform Integrating Information on Human Disease-Associated Genes and Variants’, *Nucleic Acids Research* gkw943.

Pletscher-Frankild, Sune, et al. (2015), ‘DISEASES: Text Mining and Data Integration of Disease-Gene Associations’, *Methods*, 74: 83–9, https://doi.org/10.1016/j.ymeth.2014.11.020.

Povey, S., et al. (2001), ‘The HUGO Gene Nomenclature Committee (HGNC)’, in *Human Genetics*, no. 6, preprint, 109.678–80, https://doi.org/10.1007/s00439-001-0615-0.

Quast, Christian, et al. (2012), ‘The SILVA Ribosomal RNA Gene Database Project: Improved Data Processing and Web-Based Tools’, *Nucleic Acids Research*, 41/D1: D590–6.

Schoch, Conrad L., et al. (2020), ‘NCBI Taxonomy: A Comprehensive Update on Curation, Resources and Tools’, in *Database*, vol. 2020, preprint, Oxford University Press, https://doi.org/10.1093/database/baaa062.

Schriml, Lynn Marie, et al. (2012), ‘Disease Ontology: A Backbone for Disease Semantic Integration’, *Nucleic Acids Research*, 40/D1, https://doi.org/10.1093/nar/gkr972.

Sun, Ya-Zhou, et al. (2018), ‘MDAD: A Special Resource for Microbe-Drug Associations’, *Frontiers in Cellular and Infection Microbiology*, 8: 424.

Sweeney, Blake A., et al. (2019), ‘RNAcentral: A Hub of Information for Non-Coding RNA Sequences’, *Nucleic Acids Research*, 47/D1: D221–9, https://doi.org/10.1093/nar/gky1034.

Szklarczyk, Damian, et al. (2019), ‘STRING V11: Protein–Protein Association Networks with Increased Coverage, Supporting Functional Discovery in Genome-Wide Experimental Datasets’, *Nucleic Acids Research*, 47/D1: D607–13.

Tribble, Dennis A. (2024), ‘The National Drug Code Explained’, *American Journal of Health-System Pharmacy* zxae274.

Ursu, Oleg, et al. (2016), ‘DrugCentral: Online Drug Compendium’, *Nucleic Acids Research* gkw993.

Vasilevsky, Nicole, et al. (2020), ‘Mondo Disease Ontology: Harmonizing Disease Concepts across the World’, *CEUR Workshop Proceedings, CEUR-WS*, 2807.

Wang, Yanli, et al. (2009), ‘PubChem: A Public Information System for Analyzing Bioactivities of Small Molecules’, in *Nucleic Acids Research*, vol. 37, no. SUPPL. 2, preprint, https://doi.org/10.1093/nar/gkp456.

Weisgerber, David W. (1997), ‘Chemical Abstracts Service Chemical Registry System: History, Scope, and Impacts’, *Journal of the American Society for Information Science*, 48/4: 349–60.

Wishart, David S., et al. (2022), ‘HMDB 5.0: The Human Metabolome Database for 2022’, *Nucleic Acids Research*, 50/D1: D622–31.

Wu, Cathy H., et al. (2006), ‘The Universal Protein Resource (UniProt): An Expanding Universe of Protein Information.’, *Nucleic Acids Research*, 34/Database issue, https://doi.org/10.1093/nar/gkj161.
